# Ocellatuperoxides A–F, Uncommon Anti-Tumoral *γ*-Pyrone Peroxides from a Photosynthetic Mollusk *Placobranchus ocellatus*

**DOI:** 10.3390/md20100590

**Published:** 2022-09-21

**Authors:** Song-Wei Li, Qihao Wu, Heng Xu, Li-Gong Yao, Cheng Luo, Hong Wang, Hao Zhang, Xu-Wen Li, Yue-Wei Guo

**Affiliations:** 1 State Key Laboratory of Drug Research, Shanghai Institute of Materia Medica, Chinese Academy of Sciences, Shanghai 201203, China; 2 Collaborative Innovation Center of Yangtze River Delta Region Green Pharmaceuticals, College of Pharmaceutical Science, Zhejiang University of Technology, Hangzhou 310014, China; 3 Shandong Laboratory of Yantai Drug Discovery, Bohai Rim Advanced Research Institute for Drug Discovery, Yantai 264117, China; 4 Open Studio for Druggability Research of Marine Natural Products, Qingdao National Laboratory for Marine Science and Technology, 1 Wenhai Road, Aoshanwei, Jimo, Qingdao 266237, China

**Keywords:** photosynthetic mollusk, *Placobranchus ocellatus*, *γ*-pyrone polypropionate, ocellatuperoxides, anti-tumoral activity

## Abstract

Six new pairs of *γ*-pyrone polypropionate enantiomers with an unusual peroxyl bridge at the side chain, namely (±)-ocellatuperoxides A–F (**1**–**6**), were isolated and characterized from the South China Sea photosynthetic mollusk *Placobranchus ocellatus*. Extensive spectroscopic analysis, single crystal X-ray diffraction analysis, ECD- (electronic circular dichroism) comparison, and TDDFT (time-dependent density functional theory) ECD computation were used to determine the structures and absolute configurations of new compounds. In a cell viability assay, several compounds showed considerable anti-tumoral effects on human non-small cell lung cancer cells A549 with Gefitinib (7.4 μM) and Erlotinib (2.1 μM) as positive controls. Further RNA-sequencing analysis and gene expression evaluation indicated that the anti-tumoral activity of the most effective compound **3** was associated with the regulation of several important genes, such as FGFR1 and HDAC5.

## 1. Introduction

Marine sacoglossan mollusks are small shell-less and colorful animals. The loss of the shell forces mollusks to produce small molecules in order to defend against their enemies [1]. Pyrone polypropionates, one of the defensive metabolites with complex and diverse structures, have been found in many species of the order sacoglossa [2,3]. Those metabolites can be found in the mantle or the mucus of the animals and be toxic to the predators [4]. Moreover, poly-propionates are likely functioning as natural sunscreens against harmful sun radiation and oxidative damages due to the presence of the polyunsaturated side chain [5,6,7,8]. Intriguingly, pyrone polypropionates have been widely reported to exhibit promising in vitro growth-inhibitory activity against human cancer cell lines [9,10,11,12].

**Figure 1:**
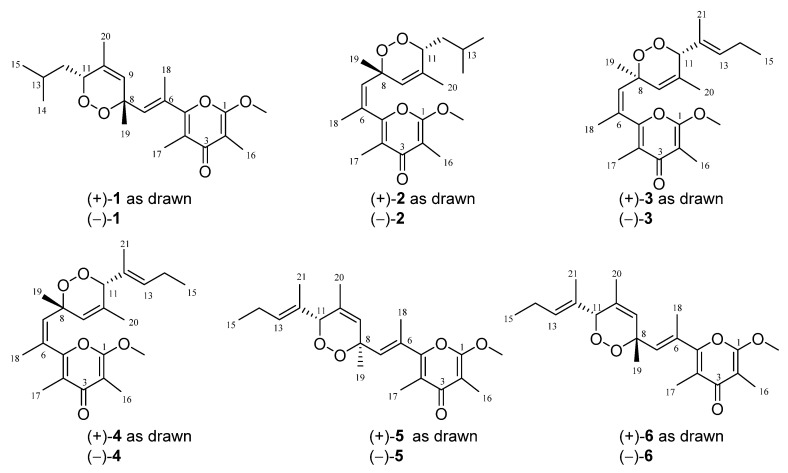
The structures of compounds (±)-**1**–**6**.

In the course of our ongoing research project with the purpose of discovering bioactive secondary metabolites from marine mollusks in the South China Sea, we have recently reported a series of racemic non-*γ*-pyrone polyketides with novel skeletons, ocellatusones A−D, isolated from the photosynthetic mollusk *Placobranchus ocellatus* (phylum Mollusca, class Gastropoda, subclass Opisthobranchia, order Sacoglossa) collected off Ximao Island, Hainan Province, China [13]. With the aim of expanding structural diversity and pharmacological application of the *γ*-pyrone polypropionates, chemistry-driven and bioassay-directed isolation of the title animals led to the discovery of six uncommon *γ*-pyrone-type polypropionates, ocellatuperoxides A–F (**1**–**6**), which have been further proved and separated by chiral HPLC (high performance liquid chromatography) to be six pairs of enantiomers [(±)-**1**–**6**] (Figure 1). Herein, we report the isolation, structural elucidation, and biological activity evaluation of these new isolates.

## 2. Results and Discussion

### 2.1. Structural Elucidation of Ocellatuperoxides A–F (***1**–**6***)

**Figure 2:**
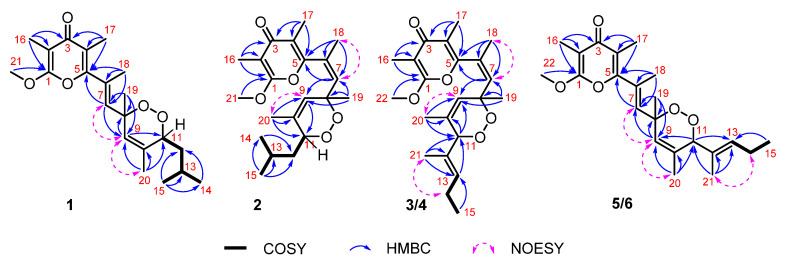
^1^H^−1^H COSY, key HMBC and NOESY correlations of compounds **1**–**6**.

The frozen bodies of *P. ocellatus* (500 specimens, 55.0 g, dry weight), collected off shallow water of Ximao Island, Hainan Province, China, were extracted. The bioactive extract gave compounds (±)-**1** (1.1 mg), (±)-**2** (1.5 mg) (±)-**3** (3.4 mg), (±)-**4** (2.3 mg), (±)-**5** (2.0 mg), and (±)-**6** (1.7 mg), respectively. Subsequently, the racemic isolates **1**–**6** were successfully separated by chiral HPLC to yield six equimolar pairs of optically pure enantiomers. The NMR (nuclear magnetic resonance) data and structural features of **1**–**6** were reminiscent of those of the previously reported polypropionates isolated from the Mediterranean sacoglossan mollusk *Placida dendritica,* namely placidenes [14], with differences on the substitution pattern of the unsaturated side chain.

(±)-Ocellatuperoxide A (**1**) was isolated as colorless crystals, and its molecular formula was determined to be C_21_H_30_O_5_ by the ion peak at *m/z* 363.2176 [M + H]^+^ (calcd. for C_21_H_31_O_5_, 363.2166) in the HR (high resolution)-ESIMS, indicating seven degrees of unsaturation. The IR spectrum (KBr, *ν* = 1655 cm^−1^) indicated the presence of an unsaturated carbonyl group. The ^13^C NMR (Table 1) and HSQC (heteronuclear single quantum coherence) spectra disclosed 21 carbon signals, including eight methyls, one sp^3^ methylene, two sp^3^ methines, one sp^3^ quaternary carbon, two sp^2^ methines, and seven sp^2^ quaternary carbons. The typical tetrasubstituted *γ*-pyrone moiety in **1** was readily identified by the characteristic NMR data of a ketone carbonyl (*δ*_C_ 181.6, qC), two tetrasubstituted double bonds (*δ*_C_ 99.6, qC, *δ*_C_ 162.0, qC; *δ*_C_ 118.1, qC, 158.5, qC), two methyls (*δ*_C_ 7.0, C-16; *δ*_C_ 11.9, C-17), and one methoxyl (*δ*_C_ 55.4, OMe), which was supported by UV spectrum (MeOH, *λ*_max_ = 260 nm, log*ε* = 3.7). In addition, ^1^H and ^13^C spectroscopic data of **1** showed typical signals from two trisubstituted double bonds (*δ*_C_ 129.0, qC, *δ*_C_ 138.9, CH; *δ*_C_ 125.5, CH, 134.5, qC). The above data accounted for six out of seven degrees of unsaturation, suggesting that **1** has a bicyclic structure. The remaining signals corresponding to two doublet methyls (*δ*_H_/*δ*_C_ 0.93, d, *J* = 6.6 Hz/23.9; *δ*_H_/*δ*_C_ 0.94, d, *J* = 6.6 Hz/21.9), three singlet methyls (*δ*_H_/*δ*_C_ 2.05, s/16.0; *δ*_H_/*δ*_C_ 1.45, s/24.7; *δ*_H_/*δ*_C_ 1.73, s/18.5), one methylene (*δ*_H_/*δ*_C_ 1.54, m; 1.42, m/39.5), one oxygenated quaternary carbon (*δ*_C_ 79.8, C-8), and one oxygenated methine (*δ*_H_/*δ*_C_ 4.45, d, *J* = 9.4 Hz/79.3, C-11) suggested the presence of an endoperoxide bridge between C-8 and C-11 to form a 1,2-dioxane ring. The COSY (homonuclear chemical shift correlation spectroscopy) correlations designated only one proton-proton spin system extending from H-11 to H_3_-15. Finally, a detail analysis of the HMBC (heteronuclear multiple bond coherence) correlations from H_3_-15 (*δ*_H_ 0.93) to C-12 (*δ*_C_ 39.5), C-13 (*δ*_C_ 24.7), and C-14 (*δ*_C_ 21.9); H_3_-16 (*δ*_H_ 1.86) to C-1 (*δ*_C_ 162.0), C-2 (*δ*_C_ 99.6), and C-3 (*δ*_C_ 181.6); H_3_-17 (*δ*_H_ 1.98) to C-3, C-4 (*δ*_C_ 118.1), and C-5 (*δ*_C_ 158.5); H_3_-18 (*δ*_H_ 2.05) to C-5, C-6 (*δ*_C_ 129.0), and C-7 (*δ*_C_ 138.9); H_3_-19 (*δ*_H_ 1.45) to C-7, C-8 (*δ*_C_ 79.8), and C-9 (*δ*_C_ 125.5); H_3_-20 (*δ*_H_ 1.73) to C-9, C-10 (*δ*_C_ 134.5), and C-11 (*δ*_C_ 79.3); -OMe (*δ*_H_ 3.95) to C-1 via two and three bonds, established the planar structure of **1** (Figure 2).

As for the stereochemistry, the trisubstituted olefins of *Δ*^6,7^ and *Δ*^9,10^ were determined to be *E* and *Z* geometries by the clear NOE (nuclear overhauser effect) correlations of H-7 (*δ*_H_ 5.80, s)/H-9 (*δ*_H_ 5.67, s), H_3_-20 (*δ*_H_ 1.73, s)/H-9, and the lack of NOE relationship between H_3_-18 (*δ*_H_ 2.05, s) and H-7, respectively.

**Figure 3:**
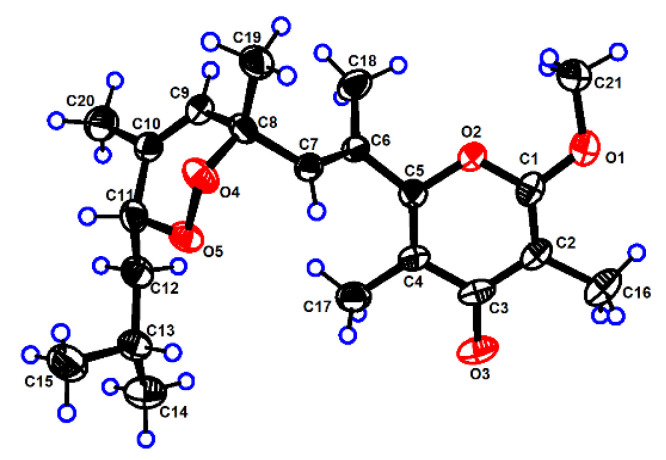
The ORTEP drawing of (±)-ocellatuperoxide A (**1**).

**Figure 4:**
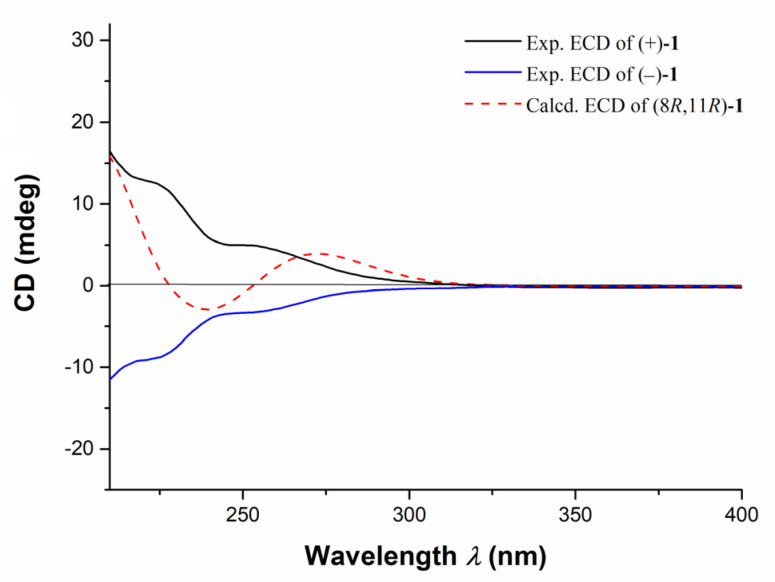
Assignments of the ACs of (±)-**1** by the comparison of TDDFT-ECD calculated spectrum and the experimental spectrum. Experimental ECD curves of (+)-**1** (black line), (−)-**1** (blue line), and calculated ECD spectrum of (8*R*,11*R*)-**1** (red dash line).

**Figure 5:**
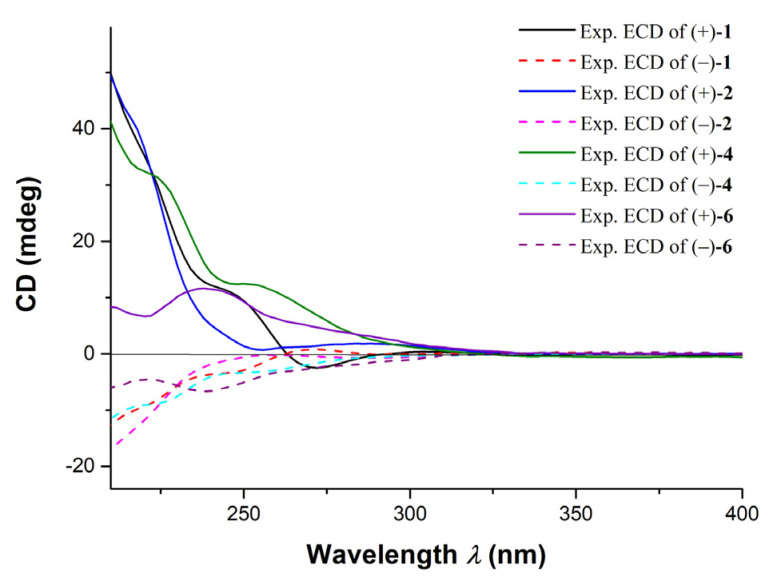
The comparison of experimental ECD spectra of compounds (±)-**1**, **2**, **4** and **6**.

The structure of **1** was further confirmed by the X-ray diffraction analysis using Mo K*α* radiation (*λ* = 0.71073) after recrystallization from methanol (Figure 3, CCDC deposition number 2,073,048). The crystals of **1** had a P-1 space group, indicating its racemic nature, as further confirmed by chiral HPLC. Analysis of the X-ray data also unambiguously determined the relative configurations of **1** as 8R*,11R*. To determine the absolute configurations (ACs) for (±)-**1**, the TDDFT-ECD calculation [15] was performed. The results indicated that the calculated ECD spectrum of (8*R*,11*R*)-**1** was in a good agreement with the experimental ECD curve of (+)-**1**, which was completely opposite to the experimental ECD spectrum of (−)-**1** (Figure 4). Consequently, the ACs of (+)-**1** and (−)-**1** were determined to be 8*R*,11*R* and 8*S*,11*S*, respectively.

(±)-Ocellatuperoxide B (**2**) had the same molecular formula as **1** (C_21_H_30_O_5_), which was determined by HR-ESIMS (*m/z* 363.2169 [M + H]^+^, calcd. for C_21_H_31_O_5_, 363.2166). Comparison of ^1^H and ^13^C chemical shifts of **1** and **2** (Table 1) followed by a detailed analysis of its 2D NMR data revealed the structure of **2** to be almost identical to that of **1**, differing from each other only at the C-18 position (*δ*_H_ 1.96, s; *δ*_C_ 23.8 in **2**; *δ*_H_ 2.05, s; *δ*_C_ 16.0 in **1**). It strongly suggested that **1** and **2** were isomers with different geometry of *Δ*^6,7^. This assumption was supported by the clear NOE correlations between H_3_-18 and H-7 (*δ*_H_ 5.83, s) in **2**, which indicated that the geometry of the *Δ*^6,7^ in **2** was *Z*. Moreover, the trisubstituted olefin of *Δ*^9,10^ was also determined to be *Z* geometry by the clear NOE correlations of H_3_-20 (*δ*_H_ 1.54, s) with H-9 (*δ*_H_ 5.27, s) (Figure 2). The almost identical NMR chemical shifts of **2** with those of compound **1** strongly suggested the same relative configuration (8R*,11R*) for **2** as that of **1**. Finally, the experimental ECD spectra of (±)-**2** showed the similar curves with that of (±)-**1** (Figure 5), respectively, deduced the ACs of (±)-**2** as shown in Figure 1.

The ^13^C NMR data (Table 2) and HSQC experiments of the other four pairs of compounds, (±)-ocellatuperoxides C–F (**3**–**6**), all disclosed 22 carbon resonances: eight methyls, one sp^3^ methylene, one sp^3^ methine, one sp^3^ quaternary carbon, three sp^2^ methines, and eight sp^2^ quaternary carbons. Further analysis of their 1D/2D NMR data with those co-occurring compounds **1** and **2** revealed the structures of **3**–**6** to be similar to that of **1** and **2** with a different side chain at C-11. Compounds **3**–**6** possess the 1-methyl-1-butenyl group, while **1** and **2** bear an isobutyl group on the side chain. Similarly, new compounds **3**–**6** only differed from each other by the geometry of the olefins at *Δ*^6,7^ and the RCs on the unsaturated side chain.

The molecular formula of (±)-ocellatuperoxide C (**3**) was determined to be C_22_H_30_O_5_ by HR-ESIMS (*m/z* 375.2176 [M + H]^+^, calcd. for C_22_H_31_O_5_, 375.2166), 12 mass units more than that of **1** and **2**, indicating eight degrees of unsaturation. Its IR spectrum exhibited strong absorptions at 1649 cm^−1^, consistent with the presence of the unsaturated carbonyl group. The typical tetrasubstituted *γ*-pyrone moiety in **3** was readily identified by the characteristic NMR data of a ketone carbonyl (*δ*_C_ 180.9, qC), two tetrasubstituted double bonds (*δ*_C_ 100.6, qC, *δ*_C_ 162.5, qC; *δ*_C_ 119.2, qC, 155.4, qC), two methyls (*δ*_C_ 7.0, C-16; *δ*_C_ 11.0, C-17), and one methoxyl (*δ*_C_ 55.7, C-22). The connections of Me-16 to C-2, Me-17 to C-4, and OMe-22 to C-1, were determined by the HMBC correlations from H_3_-16 to C-1/C-2/C-3, from H_3_-17 to C-3/C-4/C-5, and from H_3_-22 to C-1. Therefore, the side chain should connect to the C-5 position of the *γ*-pyrone. The remaining carbon signals revealed that the side chain contained three trisubstituted double bonds (*δ*_H_ 5.77 s, *δ*_C_ 136.2, CH/*δ*_C_ 130.4, qC; *δ*_H_ 5.38 s, *δ*_C_ 125.3, CH/*δ*_C_ 132.3, qC; *δ*_H_ 5.38 m, *δ*_C_ 135.6, CH; *δ*_C_ 130.4, qC), two oxygenated carbons, one methylene, and three methyls. The connection of these groups was established by the ^1^H-^1^H COSY and HMBC correlations, as shown in Figure 2. Finally, bearing in mind the remaining one degree of unsaturation and the two unassigned two oxygen atoms of **3**, the presence of an endoperoxide bridge between C-8 and C-11 was recognized, forming a 1,2-dioxane ring. Thus, the planar structure of **3** was completely determined. The geometries of trisubstituted olefins (*Δ*^6,7^, *Δ*^9,10^, *Δ*^12,13^) in **3** were determined to be *Z*, *Z* and *E* by the observation of clear NOE correlations of H_3_-18 with H-7, H_3_-20 with H-9, and H_3_-21 with H_2_-14, respectively (Figure 2). The RC of compound **3** was tentatively assigned to be 8R*,11S* based on the lack of NOE correlation between H_3_-19 (*δ*_H_ 1.31, s) and H-11 (*δ*_H_ 4.33, s).

**Figure 6:**
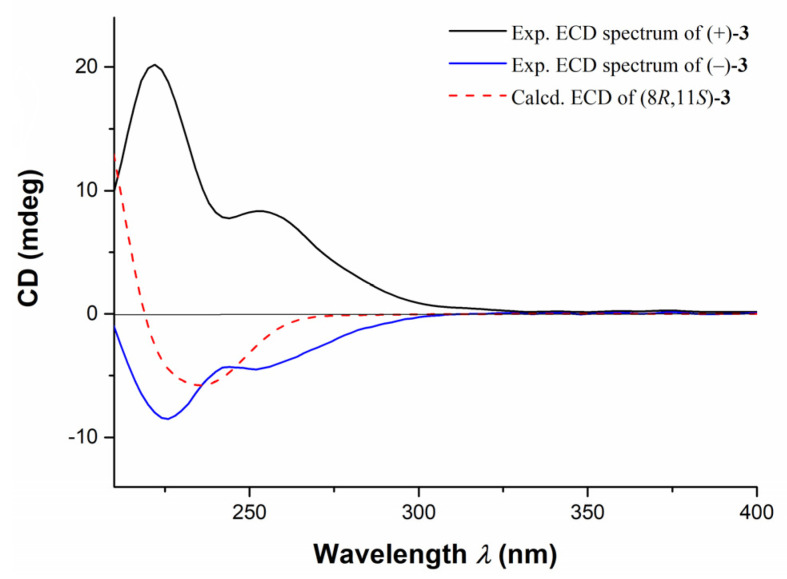
The comparison of experimental ECD spectra of compounds (±)-**3** and the calculated ECD curve of (8*R*, 11*S*)-**3**.

Further TDDFT-ECD calculations of (±)-**3** were performed. As shown in Figure 6, the calculated ECD spectrum of (8*R*,11*S*)-**3** was in good agreement with experimental ECD curve of (−)-**3**, which was opposite to the experimental ECD curve of (+)-**3**. Consequently, the structures and ACs of (±)-**3** were determined as depicted.

The molecular formula of (±)-ocellatuperoxide D (**4**) was determined to be C_22_H_30_O_5_ by HR-ESIMS (*m/z* 375.2176 [M + H]^+^, calcd. for C_22_H_31_O_5_, 375.2166), the same as that of **3**, requiring eight degrees of unsaturation. The ^1^H and ^13^C NMR data of **4** were closely similar to those of compound **3**, with the main difference on the chemical shifts of carbons near the C-8 and C-11, suggesting that they could be epimers of C-8 or C-11. Thus, the relative configurations of compound **4** were tentatively determined as (8R*,11R*,6*Z*,9*Z*,12*E*).

The ACs of (±)-**4** were determined by the comparison of ECD spectra of (±)-**4** and (±)-**2**, respectively (Figure 5). Consequently, the structures and ACs of (±)-**4** were determined as shown in Figure 1.

The molecular formula of (±)-ocellatuperoxide E (**5**) was determined to be C_22_H_30_O_5_ by HR-ESIMS (*m/z* 375.2171 [M + H]^+^, calcd. for C_22_H_31_O_5_, 375.2166), the same as that of **3** and **4**, indicating eight degrees of unsaturation. The IR and UV spectra of **5** closely resembled those of **4**, suggesting similar functionalities in the molecule. Similarly, the typical tetrasubstituted *γ*-pyrone moiety in **5** was also immediately identified by the characteristic NMR data. A comparison of ^1^H and ^13^C chemical shifts of **5** and **3** (Table 2) followed by a detailed analysis of its 2D NMR data revealed the structure of **5** to be almost identical to that of **3**, differing from each other mainly at the C-18 position (*δ*_H_ 1.96, s; *δ*_C_ 23.7 in **3**; *δ*_H_ 2.08, s; *δ*_C_ 15.9 in **5**). It strongly suggested that **3** and **5** were isomers with different geometry of *Δ*^6,7^. This assumption of *E* geometry was supported by the chemical shift of C-18 (<20 ppm) and the lack of NOE correlation of H_3_-18 and H-7 in **5**. In addition, the trisubstituted olefins of *Δ*^9,10^ and *Δ*^12,13^ were determined to be *Z* and *E* geometries by the clear NOE correlations of H_3_-20 (*δ*_H_ 1.63, s)/H-9 (*δ*_H_ 5.85, s) and of H_3_-21 (*δ*_H_ 1.45, s)/H-14 (*δ*_H_ 2.06, m), respectively (Figure 2). Furthermore, the lack of NOE correlation between H_3_-19 (*δ*_H_ 1.38, s) and H-11 (*δ*_H_ 4.85, s), suggested the RC of compound **5** was 8R*,11S*.

**Figure 7:**
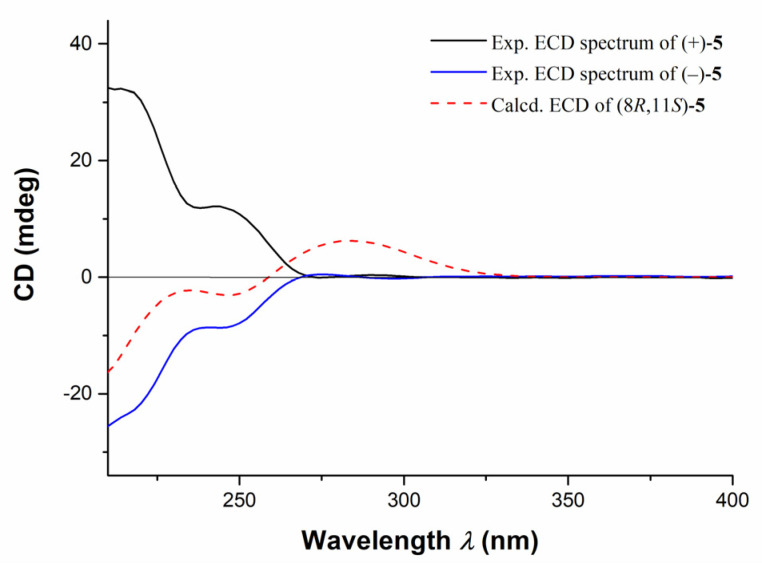
The comparison of experimental ECD spectra of compounds (±)-**5** and the calculated ECD curve of (8*R*, 11*S*)-**5**.

Further TDDFT-ECD calculations of (±)-**5** were also performed, and the results indicated that the calculated ECD spectrum of (8*R*,11*S*)-**5** were consistent with the experimental ECD curve of (−)-**5**, while the calculated ECD spectrum of (8*R*,11*S*)-**5** were completely opposite to that of (+)-**5** (Figure 7). Accordingly, the structures and ACs of (±)-**5** were elucidated as depicted.

(±)-ocellatuperoxide F (**6**), which was isolated as a colorless oil, gave the molecular formula C_22_H_30_O_5_ on the basis of the HR-ESIMS ion peak at *m/z* 375.2165 [M + H]^+^ (calcd. for C_22_H_31_O_5_, 375.2166), requiring eight degrees of unsaturation. The IR, UV and NMR data of **6** resembled to those of **5**, with the only difference appearing at the chemical shifts at carbon atoms near the C-8 and C-11 in compounds **5** and **6**, which revealed that they should also be epimers of C-8 or C-11. Thus, the planar structure of **6** was determined as shown in Figure 1, which was further confirmed by 2D NMR analysis, including ^1^H-^1^H COSY and HMBC correlations (Figure 2). The geometries of the olefins *Δ*^7,8^ *Δ*^9,10^ and *Δ*^12,13^ in **6** were determined to be *E*, *Z* and *E*, respectively, by NOESY experiment and the comparison of the chemical shift of C-18 with those of **3**–**5**. Consequently, the relative configurations of compound **6** were tentatively determined as (8R*,11R*,6*E*,9*Z*,12*E*).

The ACs of (±)-**6** were determined by the comparison of ECD spectra of (±)-**6** and (±)-**1**, respectively (Figure 5). Finally, the structures and ACs of (±)-**6** were confirmed and are shown in Figure 1.

### 2.2. Bioactivity Test of Ocellatuperoxides A–F (***1**–**6***)

In a bioassay, we first screened racemic mixtures **1**–**6** for cytotoxic effects against leukemia NB4 cells, non-small cell lung cancer (NSCLC) A549 cells, and hepatocarcinoma Hep-G2 cells. Isolates **3**–**6** were found to possess cytotoxic effects with IC_50_ values in the 10 μM range (Table 3). Among them, **3** displayed the broadest anti-tumoral activity against NB4, A549, and HepG2 cells, with IC_50_ values of 11.1, 7.8, and 8.7 μM, respectively. The preliminary structure-activity relationship study revealed that the terminal isobutyl group is not good for the activity, since **1** and **2** are inactive, whereas the different activities of **3**–**6** suggested that the stereochemistry also influenced the activity.

**Figure 8:**
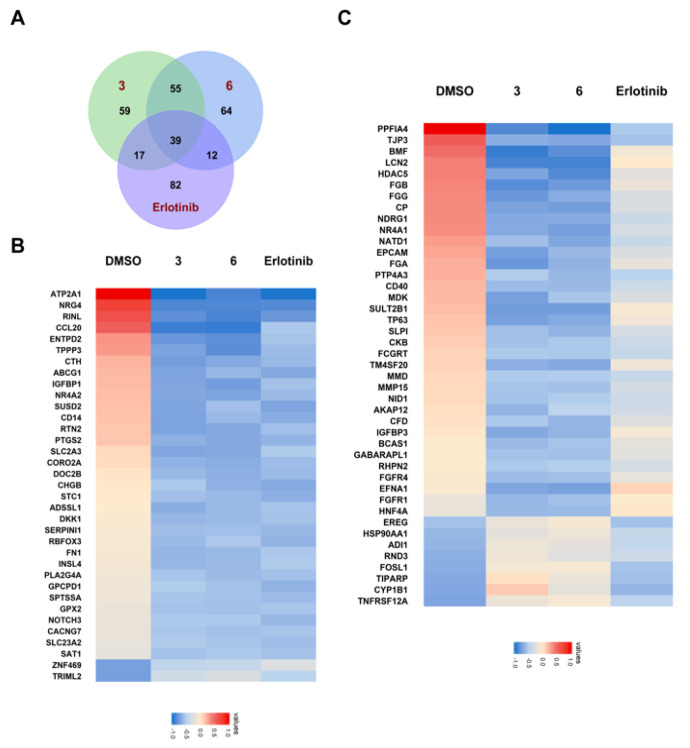
Heatmaps of differentially expressed genes revealed by RNA-Seq data. (**A**) The number of shared and unique differentially expressed genes for compounds **3** and **6**, as compared to Erlotinib. (**B**) Heatmap for the differentially expressed genes shared by the three treatment groups of compounds **3**, **6**, and Erlotinib. (**C**) Heatmap for the differentially expressed genes that are unique in the treatment groups of compounds **3** and **6**, as compared to the treatment group of Erlotinib.

Since compounds **3** and **6** showed satisfactory inhibition against the proliferation of A549, we wanted to characterize the genomic impact of them against cancer cells. The RNA-sequencing (RNA-Seq) data of **3** and **6** were then collected and analyzed (Figure 8). The number of differentially expressed genes were 170, 170 and 150 for compound **3**, **6** and Erlotinib, respectively. In addition, dozens of cancer-related differentially expressed genes were identified for **3** and **6**.

**Figure 9:**
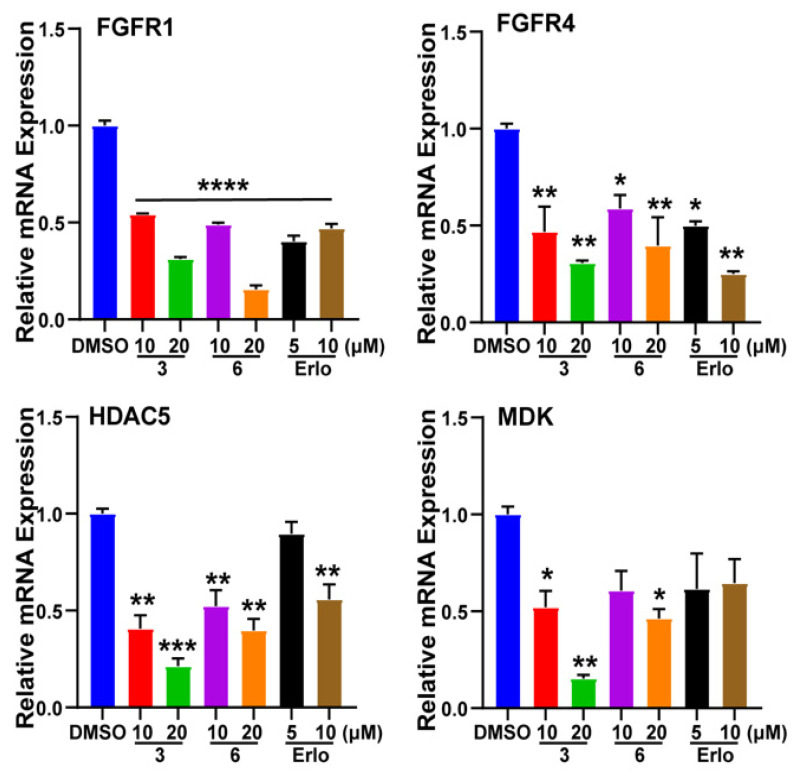
Gene expression level validation of RNA-Sequencing data. “Erlo” means Erlotinib. * *p* < 0.05; ** *p* < 0.01; *** *p* < 0.001, **** *p* < 0.0001; one-way ANOVA. Error bars, mean ± SEM.

Among these cancer-related genes of RNA-Sequencing data, the genetic knock down of FGFRs [16], HDAC5 [17], and MDK [18] showed potential in the inhibition of lung cancer proliferation and migration, and inhibitors of proteins encoded by these genes were considered as therapeutic agents for lung cancer treatment. The expression levels of the above genes were validated using the quantitative PCR (qPCR, polymerase chain reaction) method. Compound **3** and **6** showed significant effects in the inhibition of these genes (Figure 9). The results indicated that the anti-tumor effects of compounds **3** and **6** were associated with the regulation of cell proliferation and migration. Moreover, the non-small cell lung cancer cell line A549 was shown to be the most sensitive to those endoperoxides, suggesting that the efficacy of these compounds was mainly mediated via the down-regulation of related genes instead of undifferentiated cytotoxicity.

Since these compounds were further found to be racemic, we wanted to further evaluate the enantiomers of **3**–**6** with erlotinib as the positive control. However, due to the scarcity of the purified enantiomers, only (±)-**3** was evaluated on the A549 cell line. Intriguingly, the result indicated that only (–)-**3** (IC_50_ = 8.7 ± 2.4 μM) was responsible for the activity, whereas (+)-**3** (IC_50_ > 100 μM) was inactive, which indicated that the stereochemistry had great influence on the antitumoral activity.

## 3. Materials and Methods

### 3.1. General Experimental Procedures

Melting points were measured on an X-4 digital micro-melting point apparatus. The IR spectrum was recorded on a Nicolet iS50 spectrometer (Thermo Fisher Scientific, Madison, WI, USA). UV spectroscopic spectra were recorded in chromatographic grade CH_3_OH on a Varian Cary 300 UV-Vis spectrophotometer (Varian, Palo Alto, CA, USA), and peak wavelengths are reported in nm. Optical rotations were measured on a PerkinElmer 241MC polarimeter (PerkinElmer, MA, USA). ^1^H and ^13^C NMR spectra were acquired on Bruker AVANCE III 400, 500 and 600 spectrometers (Bruker, Bremen, Germany). Chemical shifts were reported with the residual CHCl_3_ (*δ*_H_ 7.26 ppm) as the internal standard for ^1^H NMR spectrometry, and CDCl_3_ (*δ*_C_ 77.2 ppm) for ^13^C NMR spectrometry. The HRESIMS spectra were recorded on an Agilent G6250 Q-TOF (Agilent, Santa Clara, CA, USA). Commercial silica gel (Qingdao Haiyang Chemical Co., Ltd., Qingdao, China, 200–300 mesh, 300–400 mesh) was used for column chromatography, and precoated silica gel GF254 plates (Sinopharm Chemical Reagent Co., Shanghai, China) were used for analytical TLC (thin layer chromatography). Sephadex LH-20 (Pharmacia Fine Chemical Co., Ltd., Piscataway, NJ, USA) was also used for column chromatography. Reversed-phase (RP) HPLC was performed on an Agilent 1260 series liquid chromatograph equipped with a DAD G1315D detector at 210 and 254 nm (Agilent, Santa Clara, CA, USA). An Agilent semi-preparative XDB-C18 column (5 μm, 250 × 9.4 mm) was employed for the purification. Chiral HPLC separation was undertaken on the same system equipped with CHIRALPAK IB N-3 column (Chiral Technologies, Inc., West Chester, PA, USA). All solvents used for column chromatography and HPLC were of analytical grade (Shanghai Chemical Reagents Co., Ltd., Shanghai, China) and chromatographic grade (Dikma Technologies Inc., Beijing, China), respectively.

### 3.2. Biological Material

The mollusk *Placobranchus ocellatus* (500 specimens) was collected off the shallow water area, Ximao Island, Hainan Province, China, in 2017. The voucher specimen (No. 17XD-12) is available for inspection at the Shanghai Institute of Materia Medica, CAS.

### 3.3. Extraction and Isolation

The frozen animals (55.0 g, dry weight) were directly extracted exhaustively with MeOH-CH_2_Cl_2_ (1:1) in sonicate at room temperature (6 × 500 mL). The organic extract was evaporated to give a brown residue, and the residue was then partitioned between H_2_O and Et_2_O. The upper layer was concentrated under reduced pressure to give a brown crude extract of 1.5 g. The resulting residue was separated into seven fractions (A–G) by gradient Silica-gel column chromatography. The resulting fractions were then fractionated into sub-fractions by a Sephadex LH-20. The sub-fraction D1 was purified by Silica-gel open column again and then semi-preparative HPLC (70% MeCN/30% H_2_O, 3.0 mL/min), yielding compounds **1**–**6** (**1**, 1.1 mg, *t*_R_ = 6.2 min; **2**, 1.5 mg, *t*_R_ = 6.6 min; **3**, 3.4 mg, *t*_R_ = 6.9 min; **4**, 2.3 mg, *t*_R_ = 7.6 min; **5**, 2.0 mg, *t*_R_ = 8.0 min; **6**, 1.7 mg, *t*_R_ = 8.4 min).

Due to the racemic nature of compounds **1**–**6**, further chiral HPLC separations were applied to get the optically pure compounds. An enantiomer analysis of (±)-ocellatuperoxides A−F (**1**–**6**) was performed on an Agilent 1260 series liquid chromatography system with a CHIRALPAK IB N-3 column, eluted with water/MeOH as mobile phase, flow rate 1.0 mL/min, UV detector set as 210 and 254 nm. About 1.0–2.0 mg of compounds **1**–**6** were dissolved in methanol (each 2.0 mL), respectively. The injection volume was 20 μL. Compounds (±)-**1** were isolated by CHIRALPAK IB N-3 column eluted with 53% water/47% MeOH: {(+)-**1**, 0.5 mg, *t*_R_ = 20.9 min, ${\left[\alpha \right]}_{D}^{19}$ +50 (*c* 0.02, CHCl_3_); (−)-**1**, 0.5 mg, *t*_R_ = 21.4 min, ${\left[\alpha \right]}_{D}^{19}$ −38 (*c* 0.02, CHCl_3_)}; Compounds (±)-**2** were isolated by CHIRALPAK IB N-3 column eluted with 50% water/50% MeOH: {(+)-**2**, 0.6 mg, *t*_R_ = 15.4 min, ${\left[\alpha \right]}_{D}^{19}$ +65 (*c* 0.06, CHCl_3_); (−)-**2**, 0.7 mg, *t*_R_ = 15.8 min, ${\left[\alpha \right]}_{D}^{19}$ −57 (*c* 0.07, CHCl_3_)}; Compounds (±)-**3** were isolated by CHIRALPAK IB N-3 column eluted with 40% water/60% MeOH: {(+)-**3**, 1.0 mg, *t*_R_ = 24.1 min, ${\left[\alpha \right]}_{D}^{19}$ +22 (*c* 0.1, CHCl_3_); (−)-**3**, 1.0 mg, *t*_R_ = 24.7 min, ${\left[\alpha \right]}_{D}^{19}$ −17 (*c* 0.1, CHCl_3_)}; Compounds (±)-**4** were isolated by CHIRALPAK IB N-3 column eluted with 55% water/45% MeOH: {(+)-**4**, 0.5 mg, *t*_R_ = 38.7 min, ${\left[\alpha \right]}_{D}^{19}$ +18 (*c* 0.05, CHCl_3_); (−)-**4**, 0.7 mg, *t*_R_ = 39.7 min, ${\left[\alpha \right]}_{D}^{20}$ −19 (*c* 0.07, CHCl_3_)}; Compounds (±)-**5** were isolated by CHIRALPAK IB N-3 column eluted with 20% water/80% MeOH: {(+)-**5**, 0.8 mg, *t*_R_ = 14.2 min, ${\left[\alpha \right]}_{D}^{19}$ +51 (*c* 0.08, CHCl_3_); (−)-**5**, 0.9 mg, *t*_R_ = 16.0 min, ${\left[\alpha \right]}_{D}^{19}$ −57 (*c* 0.09, CHCl_3_)}; Compounds (±)-**6** were isolated by CHIRALPAK IB N-3 column eluted with 20% water/80% MeOH: {(+)-**6**, 0.5 mg, *t*_R_ = 16.2 min, ${\left[\alpha \right]}_{D}^{19}$ +10 (*c* 0.1, CHCl_3_); (−)-**6**, 0.5 mg, *t*_R_ = 16.9 min, ${\left[\alpha \right]}_{D}^{19}$ −20 (*c* 0.1, CHCl_3_)}, respectively.

### 3.4. Spectroscopic Data of Compounds

(±)-Ocellatuperoxide A (**1**): Colorless crystals, mp 128–129 °C; UV (MeOH): *λ*_max_ (log *ε*) 260 (3.7) nm; IR (KBr): *v* = 2955, 2921, 2851, 1655, 1616, 1592, 1465, 1160, 1049 cm^−1^; ^1^H and ^13^C NMR data see Table 1; HRESIMS *m/z* 363.2176 [M + H]^+^ (calcd. for C_21_H_31_O_5_, 363.2166).

(±)-Ocellatuperoxide B (**2**): Colorless oil; UV (MeOH): *λ*_max_ (log *ε*) 255 (3.7) nm; IR (KBr): *v* = 2962, 2928, 2873, 1659, 1614, 1593, 1462, 1172, 1043 cm^−1^; ^1^H and ^13^C NMR data see Table 1; HRESIMS *m/z* 363.2169 [M + H]^+^ (calcd. for C_21_H_31_O_5_, 363.2166).

(±)-Ocellatuperoxide C (**3**): Colorless oil; UV (MeOH): *λ*_max_ (log *ε*) 255 (3.6) nm; IR (KBr): *v* = 2963, 2923, 2872, 1649, 1597, 1380, 1312, 1162 cm^−1^; ^1^H and ^13^C NMR data see Table 2; HRESIMS *m/z* 375.2176 [M + H]^+^ (calcd. for C_22_H_31_O_5_, 375.2166).

(±)-Ocellatuperoxide D (**4**): Colorless oil; UV (MeOH): *λ*_max_ (log *ε*) 255 (3.6) nm; IR (KBr): *v* = 2965, 2920, 2858, 1650, 1599, 1388, 1072, cm^−1^; ^1^H and ^13^C NMR data see Table 2; HRESIMS *m/z* 375.2160 [M + H]^+^ (calcd. for C_22_H_31_O_5_, 375.2166).

(±)-Ocellatuperoxide E (**5**): Colorless oil; UV (MeOH): *λ*_max_ (log *ε*) 260 (3.6) nm; IR (KBr): *v* = 2956, 2926, 2855, 1657, 1598, 1377, 1166, 1047 cm^−1^; ^1^H and ^13^C NMR data see Table 2; HRESIMS *m/z* 375.2171 [M + H]^+^ (calcd. for C_22_H_31_O_5_, 375.2166).

(±)-Ocellatuperoxide F (**6**): Colorless oil; UV (MeOH): *λ*_max_ (log *ε*) 260 (3.6) nm; IR (KBr): *v* = 2957, 2924, 2855, 1655, 1596, 1463, 1377, 1166 cm^−1^; ^1^H and ^13^C NMR data see Table 2; HRESIMS *m/z* 375.2165 [M + H]^+^ (calcd. for C_22_H_31_O_5_, 375.2166).

### 3.5. X-ray Crystallographic Analysis for Ocellatuperoxide A (***1***)

C_21_H_30_O_5_, colorless crystals were obtained from Methanol at 4 °C. *M* = 362.45 g mol^−1^, *T* = 170 K, *λ* = 0.71073, Space group *P* -1, *a* = 8.2485 (7) Å, *b* = 9.1351 (8) Å, *c* = 13.5764 (12) Å, *α* = 88.507°, *β* = 89.533°, *γ* = 80.032°, *V* = 1007.20 (15) Å^3^, *Z* = 2, *D*_calcd_ = 1.195 Mg/m^3^, μ = 0.084 mm^−1^, F(000) = 392.0. The final *R*_1_ = 0.0686 (2033), w*R*_2_ = 0.1647 (4090). The X-ray measurements were made on a Bruker D8 Venture X-ray diffractometer with Mo K*a* radiation at 170 K. The structure was solved with the ShelXT structure solution program using intrinsic phasing and refined with the ShelXL refinement package using least squares minimization. These above crystal data were deposited in the Cambridge Crystallographic Data Centre (CCDC) and assigned the accession number of **1** (CCDC 2073048). Copies of these data can be obtained free of charge via ww.ccdc.cam.ac.uk/data request/cif or from the Cambridge Crystallographic Data Centre, 12 Union Road, Cambridge CB21EZ, UK. [Fax: (+44) 1223-336-033. E-mail: deposit@ccdc.cam.ac.uk.].

### 3.6. TDDFT-ECD Calculations

A conformational search was carried out by using the torsional sampling (MCMM) method and OPLS_2005 force field in the Macromodel 9.9.223 software with the conformational search in an energy window of 21 kJ/mol. The selected low energy conformers were further optimized at the B3LYP/6-311G(d,p) level of theory, all of which were subjected to TDDFT-ECD calculations at the mPW1PW91/6-31G** level by using the Gaussian 09 program. Finally, the SpecDis 1.62 software (Berlin, Germany) was used to obtain the calculated ECD spectrum and visualize the results.

### 3.7. Cell Viability Assay

All cell lines were purchased from ATCC. Adherent cell lines were cultured in DMEM high glucose medium (Shanghai BasalMedia Technologies Co., Ltd., Shanghai, China) supplemented with 10% fetal bovine serum (FBS) in a humidified incubator at 37 °C and 5% CO_2_, while suspension cell lines were cultured in RPMI 1640 medium. Cells were seeded in 96-well flat bottom plates. Adherent cells were seeded at the density of 3000 per well, while suspension cells were seeded at the density of 10,000 per well. The viability of cells was measured by CellTiter-Glo Luminescent Cell Viability Assay (Promega) 72 h after compounds treatment. Gefitinib and erlotinib were used to be the positive controls. Compound concentrations used for IC_50_ fitting were 100, 50, 25, 12.5, 6.25, 3.125 µM. All concentrations of the compounds were assayed in triplicates.

### 3.8. RNA-Seq Data Collection and Analysis

A549 cells were treated with compounds, total RNA was isolated and purified using DNaseI (Takara, Shiga, Japan) and Dynabeads Oligo (dT)25 (Life Technologies, Shanghai, China). Then purified RNA of 100 ng was used for cDNA library construction, using the NEBNext UltraTM RNA Library Prep Kit for Illumina (NEB). The sequencing data were collected on an Illumina HiSeq 2500 instrument using the double-end sequencing mode. Reads that passed vendor quality-filtering were processed using the Tophat2 software package, with the GRCh38/hg18 Ensembl transcript set. The Cufflinks software package was then used to assemble transcripts from each replicate. Finally, the transcriptome set for all the samples was assembled using Cuffmerge, and differentially expressed genes were identified using Cuffdiff. The heatmaps were plotted using the ggplot2 package of R. RNA-Seq raw data and processed expression files have been deposited to Gene Expression Omnibus (GEO) under accession GSE132500.

### 3.9. RNA Extraction and Quantitative RT-qPCR

A549 cells were seeded in the number of 1.5 × 10^5^ per well in 6-well plates (CORNING) overnight before compounds incubation. The cells were incubated with compounds for 72 h. Total RNA of each sample was extracted by total RNA Extraction reagent (cat. R401-01 Vazyme, Nanjing, China). The extracted RNA was reversed to cDNA according to the protocols of HiScript II Q Select RT SuperMix reagent. (cat. R232-01, Vazyme, China). The qPCR reaction was performed by using SYBR qPCR Master Mix (cat. Q711-03, Vazyme, China). Gene expression was amplified with a Quant Studio 6 Flex Real-Time PCR system (ABI). The expression of target genes was normalized with Gapdh Pad (version 5.0, GraphPad Software, San Diego, CA, USA) and calculated using the ΔΔCt method. The primer sequences are listed below.
**Primers****Forward****Reverse**FGFR1CCCGTAGCTCCATATTGGACATTTGCCATTTTTCAACCAGCGFGFR4GAGGGGCCGCCTAGAGATTCAGGACGATCATGGAGCCTHDAC5TCTTGTCGAAGTCAAAGGAGCGAGGGGAACTCTGGTCCAAAGMDKAGTCGCCTCTTAGCGGATGGCCGCCCTTCTTCACCTTATGAPDHGAAGGTCGGAGTCAACGGATCCTGGAAGATGGTGATGGG

## 4. Conclusions

In summary, further chemical investigation of the South China Sea sacoglossan *P. ocellatus* has resulted in the isolation and identification of six new *γ*-pyrone-type polypropionates ocellatuperoxides A–F (**1**–**6**), which share a rare endoperoxide ring. It is interesting to note that molecules **1** and **2** differed from molecules **3**–**6** on the side chain, suggesting that they have undergone the same peroxidation with two different biogenic precursors. The intriguing specific anti-tumoral activity of the compounds on A549 cells suggested that they could be further intensively studied towards new anti-non-small cell lung cancer drug leads. Moreover, the totally different activity of (+)-**3** and (–)-**3** vividly indicated the importance of absolute configuration on the influence of biological activities.

## Figures and Tables

**Table 1:** ^1^H (600 MHz) and ^13^C (150 MHz) NMR data of compounds **1** and **2** in CDCl_3_.

No.	1		2	
	*δ*_H_ (Mult., *J* in Hz)	*δ*_C_ Mult.	*δ*_H_ (Mult., *J* in Hz)	*δ*_C_ Mult.
1	-	162.0, s	-	163.0, s
2	-	99.6, s	-	100.4, s
3	-	181.6, s	-	180.7, s
4	-	118.1, s	-	118.7, s
5	-	158.5, s	-	156.2, s
6	-	129.0, s	-	128.3, s
7	5.80 (s)	138.9, d	5.83 (s)	137.2, d
8	-	79.8, s	-	79.4, s
9	5.67 (s)	125.5, d	5.27 (s)	124.2, d
10	-	134.5, s	-	134.5, s
11	4.45 (d, 9.4)	79.3, d	4.34 (br s)	79.2, d
12	1.54 (m) 1.30 (m)	39.5, t	1.30 (m) 1.30 (m)	39.4, t
13	1.45 (m)	24.7, d	1.76 (m)	24.8, d
14	0.94 (d, 6.6)	21.9, q	0.90 (d, 6.6)	21.6, q
15	0.93 (d, 6.6)	23.9, q	0.90 (d, 6.6)	23.8, q
16	1.86 (s)	7.0, q	1.91 (s)	7.3, q
17	1.98 (s)	11.9, q	1.89 (s)	11.3, q
18	2.05 (s)	16.0, q	1.96 (s)	23.8, q
19	1.45 (s)	24.7, q	1.26 (s)	24.8, q
20	1.73 (s)	18.5, q	1.54 (s)	18.3, q
21	3.95 (s)	55.4, q	3.96 (s)	55.9, q

**Table 2:** ^1^H (600 MHz) and ^13^C (150 MHz) NMR data of compounds **3**–**6** in CDCl_3_.

No.	3		4		5		6	
	*δ*_H_ (Mult., *J* in Hz)	*δ*_C_ Mult.	*δ*_H_ (Mult., *J* in Hz)	*δ*_C_ mult.	*δ*_H_ (Mult., *J* in Hz)	*δ*_C_ Mult.	*δ*_H_ (Mult., *J* in Hz)	*δ*_C_ Mult.
1	-	162.5, s	-	162.7, s	-	162.1, s	-	162.1, s
2	-	100.6, s	-	100.4, s	-	99.6, s	-	99.6, s
3	-	180.9, s	-	181.1, s	-	181.7, s	-	181.6, s
4	-	119.2, s	-	119.0, s	-	117.9, s	-	118.1, s
5	-	155.4, s	-	155.4, s	-	158.7, s	-	158.3, s
6	-	130.4, s	-	126.6, s	-	127.4, s	-	130.5, s
7	5.77 (s)	136.2, d	6.09 (s)	138.6, d	5.91 (s)	139.8, d	5.72 (s)	137.6, d
8	-	78.7, s	-	79.4, s	-	79.9, s	-	79.5, s
9	5.38 (s)	125.3, d	5.44 (s)	126.2, d	5.85 (s)	126.8, d	5.77 (s)	126.8, d
10	-	132.3, s	-	132.7, s	-	133.1, s	-	132.4, s
11	4.33 (s)	86.9, d	4.75 (s)	87.4, d	4.85 (s)	87.5, d	4.64 (s)	86.2, d
12	-	130.4, s	-	128.9, s	-	129.0, s	-	129.8, s
13	5.38 (ov)	135.6, d	5.60 (t, 7.2)	137.3, d	5.63 (t, 7.2)	137.4, d	5.54 (t, 7.4)	136.2, d
14	2.05 (m) 2.05 (m)	21.4, t	2.06 (m) 2.06 (m)	21.4, t	2.06 (m) 2.06 (m)	21.4, t	2.10 (m) 2.10 (m)	21.4, t
15	0.95 (t, 7.5)	13.9, q	0.97 (t, 7.5)	13.8, q	0.98 (t, 7.5)	13.8, q	0.99 (t, 7.5)	13.9, q
16	1.87 (s)	7.0, q	1.89 (s)	7.1, q	1.85 (s)	7.0, q	1.85 (s)	7.0, q
17	1.87 (s)	11.0, q	1.89 (s)	11.7, q	1.98 (s)	11.8, q	1.98 (s)	12.0, q
18	1.96 (s)	23.7, q	1.95 (s)	23.6, q	2.08 (s)	15.9, q	2.06 (s)	16.1, q
19	1.31 (s)	25.0, q	1.17 (s)	23.9, q	1.38 (s)	24.1, q	1.52 (s)	25.3, q
20	1.41 (s)	18.8, q	1.49 (s)	18.0, q	1.63 (s)	18.1, q	1.65 (s)	18.7, q
21	1.56 (s)	13.0, q	1.47 (s)	11.6, q	1.45 (s)	11.5, q	1.63 (s)	12.8, q
22	3.95 (s)	55.7, q	3.92 (s)	55.7, q	3.94 (s)	55.7, q	3.95 (s)	55.5, q

**Table 3:** Inhibitory activities of **3**–**6** against three human cancer cell lines.

No.	IC_50_ (μM) ^a^		
	NB4	A549	HepG2
3	11.1 ± 1.7	7.8 ± 0.8	8.7 ± 0.7
4	16.3 ± 1.0	18.4 ± 0.2	–
5	–	14.2 ± 0.3	–
6	–	11.7 ± 0.6	–
Gefitinib ^b^	8.4 ± 0.2	7.4 ± 0.8	8.2 ± 0.1
Erlotinib ^b^	17.2 ± 3.8	2.1 ± 0.2	6.9 ± 2.2

^a^ The other compounds showed no obvious activities with IC_50_ values over 100 μM.; ^b^ Positive control.

## Data Availability

Data are contained within the article or Supplementary Material.

## Supplementary Materials

The following are available online at https://www.mdpi.com/article/10.3390/md20100590/s1, Bioassays for compounds **1**–**6**; X-ray crystallographic analysis of **1**; Chiral HPLC analysis of (±)-**1**–**6**; Specific optical rotations of (±)-**1**–**6**; 1D and 2D NMR, HRMS, UV, and IR spectra of **1**–**6**; TDDFT-ECD calculations of compounds **1**, **3** and **5**.
