# Supplementary material for: Ocellatuperoxides A–F, Uncommon Anti-Tumoral γ-Pyrone Peroxides from a Photosynthetic Mollusk Placobranchus ocellatus
Source: Mar Drugs. 2022 Sep 21;20(10):590. doi: 10.3390/md20100590 (PMC9605225; doi:10.3390/md20100590)
Supplement: Supplementary file 1 [file marinedrugs-20-00590-s001.zip › marinedrugs-1909523-supplementary.pdf]

Supplemental Information

Ocellatuperoxides A−F, Uncommon Anti-tumoral γ-Pyrone Peroxides from a Photosynthetic Mollusk *Placobranchus ocellatus*

Song-Wei Li[1,2,†], Qihao Wu[1,†], Heng Xu[1], Li-Gong Yao[1,3,4], Cheng Luo[1,4], Hong Wang[2], Hao Zhang[1,*], Xu-Wen Li[1,3,4,*], and Yue-Wei Guo[1,2,3,4,*]

[1] State Key Laboratory of Drug Research, Shanghai Institute of Materia Medica, Chinese Academy of Sciences, Shanghai 201203, China; songweili93@zjut.edu.cn (S.-W.L.); qihao.wu@yale.edu (Q.W.); idaheng@simm.ac.cn (H.X.); yaoligong@simm.ac.cn (L.-G.Y.); cluo@simm.ac.cn (C.L.)

[2] Collaborative Innovation Center of Yangtze River Delta Region Green Pharmaceuticals, College of Pharmaceutical Science, Zhejiang University of Technology, Hangzhou 310014, China; hongw@zjut.edu.cn (H.W.)

[3] Shandong Laboratory of Yantai Drug Discovery, Bohai Rim Advanced Research Institute for Drug Discovery, Yantai，Shandong 264117, China

[4] Open Studio for Druggability Research of Marine Natural Products, Qingdao National Laboratory for Marine Science and Technology, 1 Wenhai Road, Aoshanwei, Jimo, Qingdao 266237, China

* Correspondence: hao_cadd@simm.ac.cn (H.Z.); xwli@simm.ac.cn (X.-W.L.); ywguo@simm.ac.cn (Y.-W.G.); Tel.: +86-21-50805813 (Y.-W.G.)

[†] These authors contributed equally to this work

Contents

1. Original data for ocellatuperoxides A−F (**1**−**6**) 3

1.1 Bioassays for ocellatuperoxides A–F (**1**–**6**) 3

1.2 X-ray crystallographic analysis for ocellatuperoxide A (**1**) 4

1.3 Chiral HPLC analysis chromatography of ocellatuperoxides A–F (**1**–**6**) 9

1.4 Specific optical rotations of ocellatuperoxides A–F (**1**–**6**) 11

1.5 NMR, HR-ESI-MS, IR, and UV spectra of ocellatuperoxide A (**1**) 15

1.6 NMR, HR-ESI-MS, IR, and UV spectra of ocellatuperoxide B (**2**) 24

1.7 NMR, HR-ESI-MS, IR, and UV spectra of ocellatuperoxide C (**3**) 33

1.8 NMR, HR-ESI-MS, IR, and UV spectra of ocellatuperoxide D (**4**) 42

1.9 NMR, HR-ESI-MS, IR, and UV spectra of ocellatuperoxide E (**5**) 51

1.10 NMR, HR-ESI-MS, IR, and UV spectra of ocellatuperoxide F (**6**) 60

2. Computational Section 69

2.1 Computational details 69

2.3 Cartesian Coordinates, Relative Energies, and Boltzmann populations of all the calculated Low-energy conformers 70

1. Original data for ocellatuperoxides A–F (1–6)

1.1 Bioassays for ocellatuperoxides A–F (1–6)

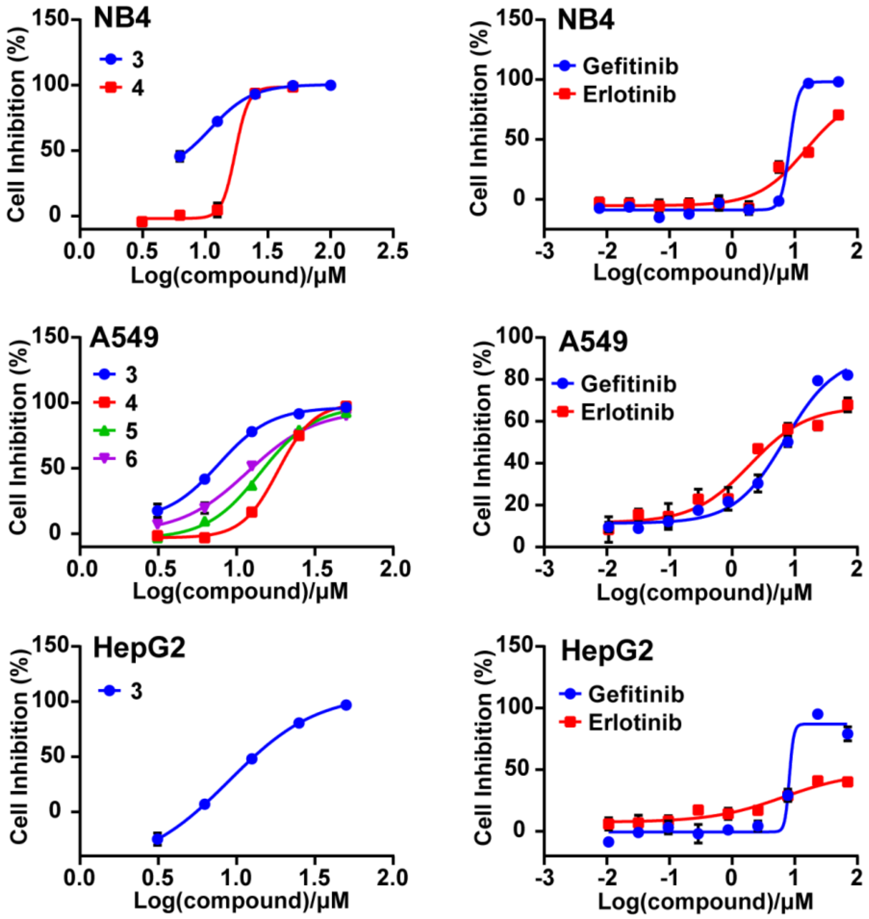

Figure S1. Racemic mixtures **3**–**6** for cytotoxic effects against leukemia NB4 cells, non-small cell lung cancer (NSCLC) A549 cells, and hepatocarcinoma Hep-G2 cells.

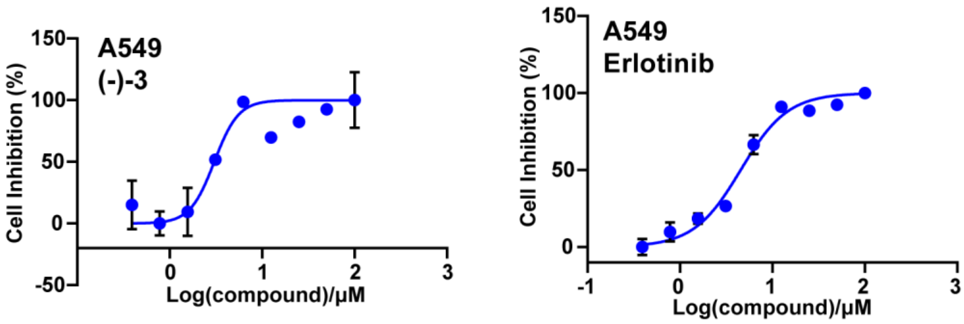

Figure S2. Purified enantiomer (−)-**3** for cytotoxic effects against non-small cell lung cancer (NSCLC) A549 cells.

1.2 X-ray crystallographic analysis for ocellatuperoxide A (1)

Table S1. X-ray crystallographic data for **1**

Identification code	0922_0m
Empirical formula	$C_{21}H_{30}O_5$
Formula weight	362.45
Temperature/K	170.0
Crystal system	triclinic
Space group	P-1
a/Å	8.2485(7)
b/Å	9.1351(8)
c/Å	13.5764(12)
α/°	88.507(3)
β/°	89.533(3)
γ/ °	80.032(2)
Volume/Å^3	1007.20(15)
Z	2
ρ_{calc}g/cm^3	1.195
μ/mm^{-1}	0.084
F(000)	392.0
Crystal size/mm^3	0.12 × 0.08 × 0.05
Radiation	MoKα (λ = 0.71073)
2Θ range for data collection/°	4.528 to 52.784
Index ranges	$-10 \le h \le 10$, $-11 \le k \le 11$, $-16 \le l \le 16$
Reflections collected	11528
Independent reflections	4090 [R_{int} = 0.0833, R_{sigma} = 0.1134]
Data/restraints/parameters	4090/0/243
Goodness-of-fit on F^2	1.043
Final R indexes [I>=2σ (I)]	R_1 = 0.0686, wR_2 = 0.1227
Final R indexes [all data]	R_1 = 0.1658, wR_2 = 0.1647
Largest diff. peak/hole / e Å^{-3}	0.21/-0.26

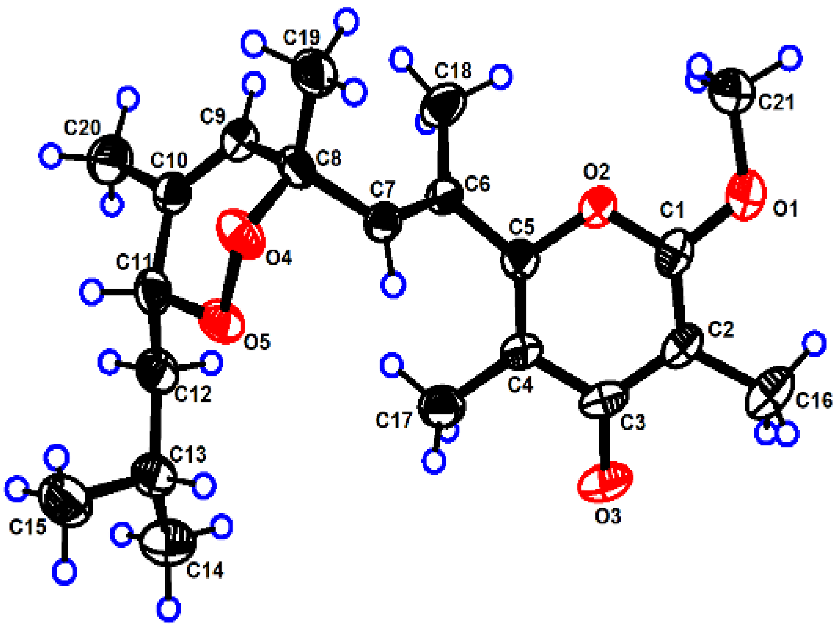

Figure S3. X-ray Crystallographic structure of compound **1**

Table S2. The detailed tables containing 1D NMR data and 2D correlations of compounds **1−6.**

**No.**	**1**				
	***δ*H (mult., *J* in Hz)**	***δ*C mult.**	**^{1}H-^{1}H COSY**	**HMBC (H→C)**	**NOESY**
1	-	162.0, s	-	-	-
2	-	99.6, s	-	-	-
3	-	181.6, s	-	-	-
4	-	118.1, s	-	-	-
5	-	158.5, s	-	-	-
6	-	129.0, s	-	-	-
7	5.80 (s)	138.9, d	18	5, 8, 9, 18	9, 19
8	-	79.8, s	-	-	-
9	5.67 (s)	125.5, d	20	8, 11, 20	7, 18, 19, 20
10	-	134.5, s	-	-	-
11	4.45 (d, 9.4)	79.3, d	12	No detected	12, 13, 14, 15, 20
12	1.54 (m)<br>1.30 (m)	39.5, t	11, 13		11, 13, 14, 15
13	1.45 (m)	24.7, d	12, 14, 15		12, 14, 15
14	0.94 (d, 6.6)	21.9, q	13	12, 13, 15	12, 13, 15
15	0.93 (d, 6.6)	23.9, q	13	12, 13, 15	12, 13, 14
16	1.86 (s)	7.0, q	-	1, 2, 3	
17	1.98 (s)	11.9, q	-	3, 4, 5	
18	2.05 (s)	16.0, q	-	5, 6, 7	
19	1.45 (s)	24.7, q	-	7, 8, 9	7, 9
20	1.73 (s)	18.5, q	-	9, 10, 11	9, 11
21	3.95 (s)	55.4, q	-	1	

**No.**	**2**				
	***δ*H (mult., *J* in Hz)**	***δ*C mult.**	**^{1}H-^{1}H COSY**	**HMBC (H→C)**	**NOESY**
1	-	163.0, s	-	-	-

2	-	100.4, s	-	-	-
3	-	180.7, s	-	-	-
4	-	118.7, s	-	-	-
5	-	156.2, s	-	-	-
6	-	128.3, s	-	-	-
7	5.83 (s)	137.2, d	18	5, 9, 18	18, 19
8	-	79.4, s	-	-	-
9	5.27 (s)	124.2, d	20	11, 19, 20	19, 20
10	-	134.5, s	-	-	-
11	4.34 (br s)	79.2, d	12	No detected	12, 14, 15, 20
12	1.30 (m)<br>1.30 (m)	39.4, t	11, 13		11, 13, 14, 15, 20
13	1.76 (m)	24.8, d	12, 14, 15		12, 14, 15
14	0.90 (d, 6.6)	21.6, q	13	12, 13, 15	11, 12, 13, 15
15	0.90 (d, 6.6)	23.8, q	13	12, 13, 15	11, 12, 13, 14
16	1.91 (s)	7.3, q	-	1, 2, 3	
17	1.89 (s)	11.3, q	-	3, 4, 5	
18	1.96 (s)	23.8, q	-	5, 6, 7	7
19	1.26 (s)	24.8, q	-	7, 8, 9	7, 9
20	1.54 (s)	18.3, q	-	9, 10, 11	9, 11
21	3.96 (s)	55.9, q	-	1	

**No.**	**3**				
	**δ_H (mult., J in Hz)**	**δ_C mult.**	**^{1}H-^{1}H COSY**	**HMBC (H→C)**	**NOESY**
1	-	162.5, s	-	-	-
2	-	100.6, s	-	-	-
3	-	180.9, s	-	-	-
4	-	119.2, s	-	-	-
5	-	155.4, s	-	-	-
6	-	130.4, s	-	-	-
7	5.77 (s)	136.2, d	18	5, 9, 18	18, 19
8	-	78.7, s	-	-	-
9	5.38 (s)	125.3, d	20	7, 11, 20	20
10	-	132.3, s	-	-	-
11	4.33 (s)	86.9, d		9, 10, 13, 21	13, 20, 21
12	-	130.4, s	-	-	-
13	5.38 (ov)	135.6, d	14	11, 15, 21	11, 14
14	2.05 (m)<br>2.05 (m)	21.4, t	13, 15	12, 13, 15	15, 21
15	0.95 (t, 7.5)	13.9, q	14	13, 14	14
16	1.87 (s)	7.0, q	-	1, 2, 3	
17	1.87 (s)	11.0, q	-	3, 4, 5	
18	1.96 (s)	23.7, q	-	5, 6, 7	7
19	1.31 (s)	25.0, q	-	7, 8, 9	7, 9
20	1.41 (s)	18.8, q	-	9, 10, 11	9, 11
21	1.56 (s)	13.0, q	-	11, 12, 13	14
22	3.95 (s)	55.7, q	-	1	

**No.**	**4**				
	**δ_H (mult., J in Hz)**	**δ_C mult.**	**^{1}H-^{1}H COSY**	**HMBC (H→C)**	**NOESY**
1	-	162.7, s	-	-	-
2	-	100.4, s	-	-	-
3	-	181.1, s	-	-	-
4	-	119.0, s	-	-	-
5	-	155.4, s	-	-	-
6	-	126.6, s	-	-	-
7	6.09 (s)	138.6, d	18	5, 9, 18, 19	18, 19
8	-	79.4, s	-	-	-
9	5.44 (s)	126.2, d	20	7, 11, 19, 20	19, 20
10	-	132.7, s	-	-	-
11	4.75 (s)	87.4, d		9, 10, 13, 20, 21	13, 20
12	-	128.9, s	-	-	-

13	5.60 (t, 7.2)	137.3, d	14	11, 21	11, 14, 15
14	2.06 (m)<br>2.06 (m)	21.4, t	13, 15		13, 15, 21
15	0.97 (t, 7.5)	13.8, q	14	13, 14	13, 14
16	1.89 (s)	7.1, q	-	1, 2, 3	
17	1.89 (s)	11.7, q	-	3, 4, 5	
18	1.95 (s)	23.6, q	-	5, 6, 7	7
19	1.17 (s)	23.9, q	-	7, 8, 9	7, 9
20	1.49 (s)	18.0, q	-	9, 10, 11	9, 11
21	1.47 (s)	11.6, q	-	11, 12, 13	14
22	3.92 (s)	55.7, q	-	1	

**No.**	**5**				
	**δ_H (mult., *J* in Hz)**	**δ_C mult.**	**^{1}H-^{1}H COSY**	**HMBC (H→C)**	**NOESY**
1	-	162.1, s	-	-	-
2	-	99.6, s	-	-	-
3	-	181.7, s	-	-	-
4	-	117.9, s	-	-	-
5	-	158.7, s	-	-	-
6	-	127.4, s	-	-	-
7	5.91 (s)	139.8, d	18	5, 18	9, 19
8	-	79.9, s	-	-	-
9	5.85 (s)	126.8, d	20	8, 11, 20	7, 19, 20
10	-	133.1, s	-	-	-
11	4.85 (s)	87.5, d		13	13
12	-	129.0, s	-	-	-
13	5.63 (t, 7.2)	137.4, d	14	11, 21	11, 14, 15
14	2.06 (m)<br>2.06 (m)	21.4, t	13, 15	12, 13, 15	13, 15, 21
15	0.98 (t, 7.5)	13.8, q	14	13, 14	13, 14
16	1.85 (s)	7.0, q	-	1, 2, 3	
17	1.98 (s)	11.8, q	-	3, 4, 5	
18	2.08 (s)	15.9, q	-	5, 6, 7	9
19	1.38 (s)	24.1, q	-	7, 8, 9	9
20	1.63 (s)	18.1, q	-	9, 10, 11	9
21	1.45 (s)	11.5, q	-	11, 12, 13	14
22	3.94 (s)	55.7, q	-	1	

**No.**	**6**				
	**δ_H (mult., *J* in Hz)**	**δ_C mult.**	**^{1}H-^{1}H COSY**	**HMBC (H→C)**	**NOESY**
1	-	162.1, s	-	-	-
2	-	99.6, s	-	-	-
3	-	181.6, s	-	-	-
4	-	118.1, s	-	-	-
5	-	158.3, s	-	-	-
6	-	130.5, s	-	-	-
7	5.72 (s)	137.6, d	18	5, 8, 18	9, 17, 19
8	-	79.5, s	-	-	-
9	5.77 (s)	126.8, d	20	8, 11, 20	7, 18, 19
10	-	132.4, s	-	-	-
11	4.64 (s)	86.2, d		No detected	13, 20
12	-	129.8, s	-	-	-
13	5.54 (t, 7.4)	136.2, d	14	14	11, 14, 15
14	2.10 (m)<br>2.10 (m)	21.4, t	13, 15	13, 15	13, 15, 21
15	0.99 (t, 7.5)	13.9, q	14	13	13, 14
16	1.85 (s)	7.0, q	-	1, 2, 3	
17	1.98 (s)	12.0, q	-	3, 4, 5	7
18	2.06 (s)	16.1, q	-	5, 6, 7	
19	1.52 (s)	25.3, q	-	7, 8, 9	7, 9
20	1.65 (s)	18.7, q	-	9, 10, 11	9
21	1.63 (s)	12.8, q	-	11, 12, 13	14

22	3.95 (s)	55.5, q	-	1

1.3 Chiral HPLC analysis chromatography of ocellatuperoxides A–F (1–6)

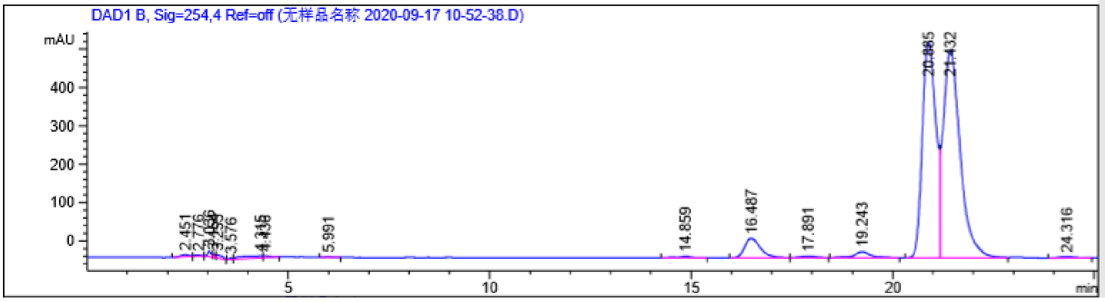

Figure S4. Chiral HPLC analysis chromatography of compounds (±)-**1**. Isocratic elution method with 53% water/47% MeOH, 1.0 mL/min. (+)-**1**, t_R= 20.9 min, (–)-**1**, t_R= 21.4 min.

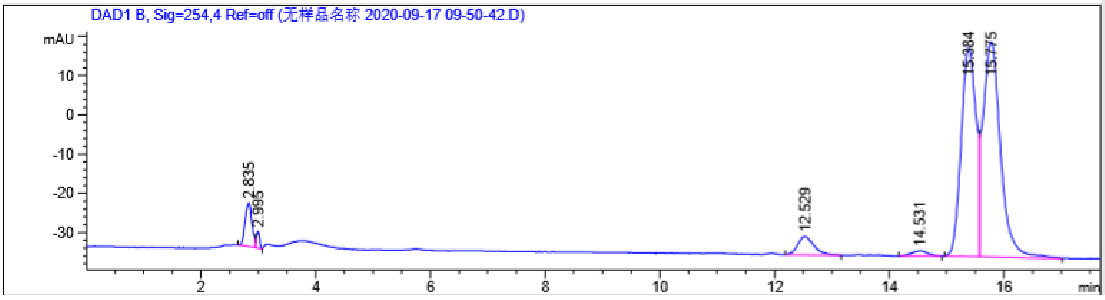

Figure S5. Chiral HPLC analysis chromatography of compounds (±)-**2**. Isocratic elution method with 50% water/50% MeOH, 1.0 mL/min. (+)-**2**, t_R= 15.4 min, (–)-**2**, t_R= 15.8 min.

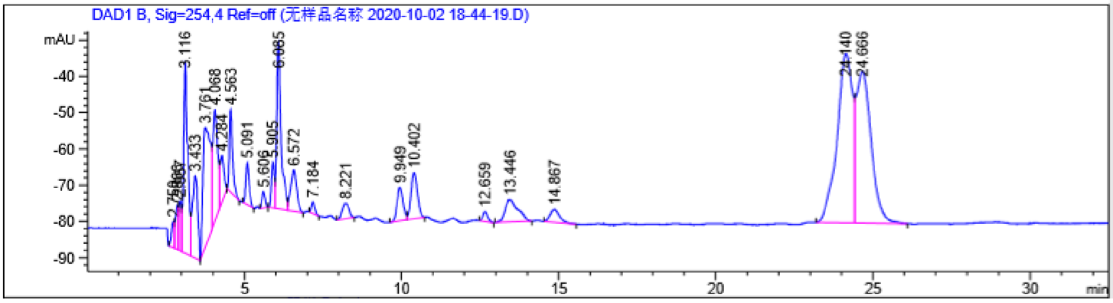

Figure S6. Chiral HPLC analysis chromatography of compounds (±)-**3**. Isocratic elution method with 40% water/60% MeOH, 1.0 mL/min. (+)-**3**, t_R= 24.1 min, (–)-**3**, t_R= 24.7 min.

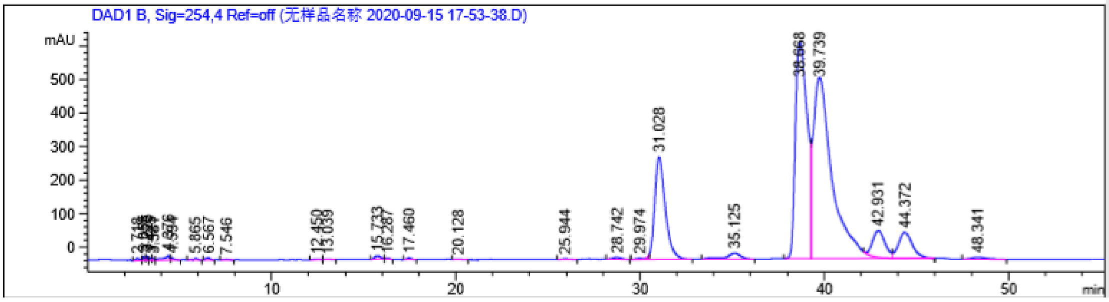

Figure S7. Chiral HPLC analysis chromatography of compounds (±)-**4**. Isocratic elution method with 55% water/45% MeOH, 1.0 mL/min. (+)-**4**, t_R= 38.7 min, (–)-**4**, t_R= 39.7 min.

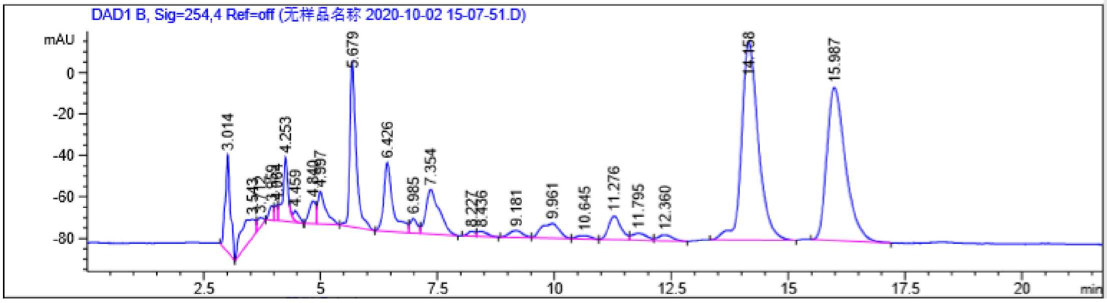

Figure S8. Chiral HPLC analysis chromatography of compounds (±)-**5**. Isocratic elution method with 20% water/80% MeOH, 1.0 mL/min. (+)-**5**, t_R= 14.2 min, (–)-**5**, t_R= 16.0 min.

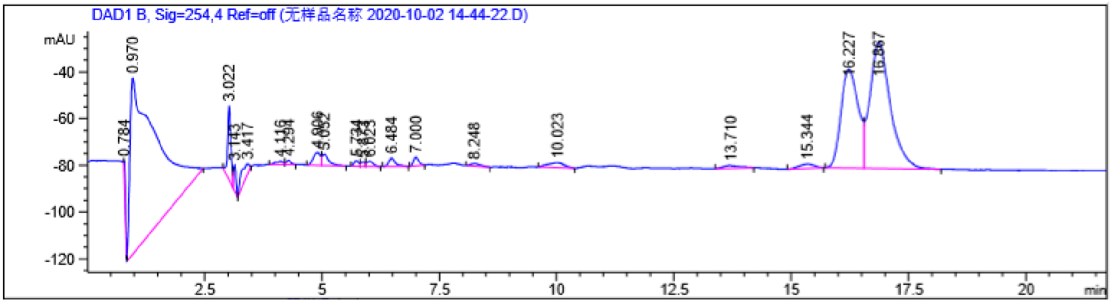

Figure S9. Chiral HPLC analysis chromatography of compounds (±)-**6**. Isocratic elution method with 20% water/80% MeOH, 1.0 mL/min. (+)-**6**, t_R= 16.2 min, (–)-**6**, t_R= 16.9 min.

1.4 Specific optical rotations of ocellatuperoxides A–F (1–6)

LotID : 001-chiral-A
Set Temperature : OFF
Temp Corr : OFF

n	Average	Std.Dev.	Maximum	Minimum
6	50.000	5.7735	60.000	40.000

S.No	Sample ID	Time	Result	Scale	OR ° Arc	WLG	Lg.mm	Conc.	Temp.	Comment
1	001-chiral-A	09:18:21 AM	40.000	SR	0.008	589	100.00	0.020	18.4	
2	001-chiral-A	09:18:35 AM	50.000	SR	0.010	589	100.00	0.020	18.4	
3	001-chiral-A	09:18:41 AM	50.000	SR	0.010	589	100.00	0.020	18.4	
4	001-chiral-A	09:18:47 AM	50.000	SR	0.010	589	100.00	0.020	18.4	
5	001-chiral-A	09:18:54 AM	50.000	SR	0.010	589	100.00	0.020	18.4	
6	001-chiral-A	09:19:00 AM	60.000	SR	0.012	589	100.00	0.020	18.4	

Figure S10. Specific optical rotation of (+)-**1**.

LotID : 001-chiral-B
Set Temperature : OFF
Temp Corr : OFF

n	Average	Std.Dev.	Maximum	Minimum
6	-37.500	3.8188	-35.000	-45.000

S.No	Sample ID	Time	Result	Scale	OR ° Arc	WLG	Lg.mm	Conc.	Temp.	Comment
1	001-chiral-B	09:24:46 AM	-45.000	SR	-0.009	589	100.00	0.020	18.5	
2	001-chiral-B	09:24:52 AM	-40.000	SR	-0.008	589	100.00	0.020	18.5	
3	001-chiral-B	09:24:58 AM	-35.000	SR	-0.007	589	100.00	0.020	18.5	
4	001-chiral-B	09:25:05 AM	-35.000	SR	-0.007	589	100.00	0.020	18.5	
5	001-chiral-B	09:25:11 AM	-35.000	SR	-0.007	589	100.00	0.020	18.5	
6	001-chiral-B	09:25:16 AM	-35.000	SR	-0.007	589	100.00	0.020	18.5	

Figure S11. Specific optical rotation of (–)-**1**.

LotID : 002-chiral-A
Set Temperature : OFF
Temp Corr : OFF

n	Average	Std.Dev.	Maximum	Minimum
6	64.722	0.6213	65.000	63.333

S.No	Sample ID	Time	Result	Scale	OR ° Arc	WLG	Lg.mm	Conc.	Temp.	Comment
1	002-chiral-A	08:18:01 PM	63.333	SR	0.038	589	100.00	0.060	18.6	
2	002-chiral-A	08:18:07 PM	65.000	SR	0.039	589	100.00	0.060	18.6	
3	002-chiral-A	08:18:13 PM	65.000	SR	0.039	589	100.00	0.060	18.6	
4	002-chiral-A	08:18:18 PM	65.000	SR	0.039	589	100.00	0.060	18.6	
5	002-chiral-A	08:18:24 PM	65.000	SR	0.039	589	100.00	0.060	18.6	
6	002-chiral-A	08:18:31 PM	65.000	SR	0.039	589	100.00	0.060	18.6	

Figure S12. Specific optical rotation of (+)-**2**.

LotID : 002-chiral-B
Set Temperature : OFF
Temp Corr : OFF

n	Average	Std.Dev.	Maximum	Minimum
6	-57.143	0.0000	-57.143	-57.143

S.No	Sample ID	Time	Result	Scale	OR ° Arc	WLG	Lg.mm	Conc.	Temp.	Comment
1	002-chiral-B	08:10:45 PM	-57.143	SR	-0.040	589	100.00	0.070	18.6	
2	002-chiral-B	08:10:51 PM	-57.143	SR	-0.040	589	100.00	0.070	18.6	
3	002-chiral-B	08:10:57 PM	-57.143	SR	-0.040	589	100.00	0.070	18.6	
4	002-chiral-B	08:11:03 PM	-57.143	SR	-0.040	589	100.00	0.070	18.6	
5	002-chiral-B	08:11:09 PM	-57.143	SR	-0.040	589	100.00	0.070	18.6	
6	002-chiral-B	08:11:15 PM	-57.143	SR	-0.040	589	100.00	0.070	18.6	

Figure S13. Specific optical rotation of (–)-**2**.

LotID : 003-chiral-A
Set Temperature : OFF
Temp Corr : OFF

n	Average	Std.Dev.	Maximum	Minimum
6	21.833	1.6750	23.000	19.000

S.No	Sample ID	Time	Result	Scale	OR ° Arc	WLG	Lg.mm	Conc.	Temp.	Comment
1	003-chiral-A	07:55:30 PM	19.000	SR	0.019	589	100.00	0.100	18.9	
2	003-chiral-A	07:55:36 PM	20.000	SR	0.020	589	100.00	0.100	18.9	
3	003-chiral-A	07:55:44 PM	23.000	SR	0.023	589	100.00	0.100	18.9	
4	003-chiral-A	07:55:50 PM	23.000	SR	0.023	589	100.00	0.100	18.9	
5	003-chiral-A	07:55:56 PM	23.000	SR	0.023	589	100.00	0.100	18.9	
6	003-chiral-A	07:56:02 PM	23.000	SR	0.023	589	100.00	0.100	18.9	

Figure S14. Specific optical rotation of (+)-**3**.

LotID : 003-chiral-B
Set Temperature : OFF
Temp Corr : OFF

n	Average	Std.Dev.	Maximum	Minimum
6	-16.500	0.5000	-16.000	-17.000

S.No	Sample ID	Time	Result	Scale	OR ° Arc	WLG	Lg.mm	Conc.	Temp.	Comment
1	003-chiral-B	08:01:17 PM	-17.000	SR	-0.017	589	100.00	0.100	19.1	
2	003-chiral-B	08:01:23 PM	-17.000	SR	-0.017	589	100.00	0.100	19.1	
3	003-chiral-B	08:01:29 PM	-17.000	SR	-0.017	589	100.00	0.100	19.1	
4	003-chiral-B	08:01:35 PM	-16.000	SR	-0.016	589	100.00	0.100	19.1	
5	003-chiral-B	08:01:41 PM	-16.000	SR	-0.016	589	100.00	0.100	19.1	
6	003-chiral-B	08:01:47 PM	-16.000	SR	-0.016	589	100.00	0.100	19.1	

Figure S15. Specific optical rotation of (–)-**3**.

LotID : 4-chiral-A
Set Temperature : OFF
Temp Corr : OFF

n	Average	Std.Dev.	Maximum	Minimum
6	18.000	1.1547	20.000	16.000

S.No	Sample ID	Time	Result	Scale	OR ° Arc	WLG	Lg.mm	Conc.	Temp.	Comment
1	4-chiral-A	06:45:58 PM	18.000	SR	0.009	589	100.00	0.050	19.4	
2	4-chiral-A	06:46:04 PM	16.000	SR	0.008	589	100.00	0.050	19.4	
3	4-chiral-A	06:46:10 PM	18.000	SR	0.009	589	100.00	0.050	19.4	
4	4-chiral-A	06:46:16 PM	18.000	SR	0.009	589	100.00	0.050	19.4	
5	4-chiral-A	06:46:22 PM	20.000	SR	0.010	589	100.00	0.050	19.4	
6	4-chiral-A	06:46:28 PM	18.000	SR	0.009	589	100.00	0.050	19.4	

Figure S16. Specific optical rotation of (+)-**4**.

LotID : 4-chiral-B
Set Temperature : OFF
Temp Corr : OFF

n	Average	Std.Dev.	Maximum	Minimum
6	-18.572	1.6496	-17.143	-21.429

S.No	Sample ID	Time	Result	Scale	OR ° Arc	WLG	Lg.mm	Conc.	Temp.	Comment
1	4-chiral-B	06:51:35 PM	-21.429	SR	-0.015	589	100.00	0.070	19.5	
2	4-chiral-B	06:51:41 PM	-20.000	SR	-0.014	589	100.00	0.070	19.5	
3	4-chiral-B	06:51:47 PM	-18.571	SR	-0.013	589	100.00	0.070	19.5	
4	4-chiral-B	06:51:53 PM	-17.143	SR	-0.012	589	100.00	0.070	19.5	
5	4-chiral-B	06:51:58 PM	-17.143	SR	-0.012	589	100.00	0.070	19.5	
6	4-chiral-B	06:52:04 PM	-17.143	SR	-0.012	589	100.00	0.070	19.5	

Figure S17. Specific optical rotation of (–)-**4**.

LotID : 005-chiral-A
Set Temperature : OFF
Temp Corr : OFF

n	Average	Std.Dev.	Maximum	Minimum
6	50.625	0.6250	51.250	50.000

S.No	Sample ID	Time	Result	Scale	OR ° Arc	WLG	Lg.mm	Conc.	Temp.	Comment
1	005-chiral-A	08:52:47 PM	50.000	SR	0.040	589	100.00	0.080	18.9	
2	005-chiral-A	08:52:56 PM	51.250	SR	0.041	589	100.00	0.080	18.9	
3	005-chiral-A	08:53:02 PM	51.250	SR	0.041	589	100.00	0.080	18.9	
4	005-chiral-A	08:53:08 PM	51.250	SR	0.041	589	100.00	0.080	18.9	
5	005-chiral-A	08:53:14 PM	50.000	SR	0.040	589	100.00	0.080	18.9	
6	005-chiral-A	08:53:20 PM	50.000	SR	0.040	589	100.00	0.080	18.9	

Figure S18. Specific optical rotation of (+)-**5**.

LotID : 005-chiral-B
Set Temperature : OFF
Temp Corr : OFF

n	Average	Std.Dev.	Maximum	Minimum
6	-56.667	4.7139	-46.667	-61.111

S.No	Sample ID	Time	Result	Scale	OR ° Arc	WLG	Lg.mm	Conc.	Temp.	Comment
1	005-chiral-B	08:46:03 PM	-46.667	SR	-0.042	589	100.00	0.090	19.1	
2	005 chiral-B	08:46:11 PM	-56.667	SR	-0.051	589	100.00	0.090	19.1	
3	005-chiral-B	08:46:21 PM	-60.000	SR	-0.054	589	100.00	0.090	19.1	
4	005-chiral-B	08:46:27 PM	-61.111	SR	-0.055	589	100.00	0.090	19.1	
5	005-chiral-B	08:46:33 PM	-57.778	SR	-0.052	589	100.00	0.090	19.1	
6	005-chiral-B	08:46:39 PM	-57.778	SR	-0.052	589	100.00	0.090	19.1	

Figure S19. Specific optical rotation of (–)-**5**.

LotID : 006-chiral-A
Set Temperature : OFF
Temp Corr : OFF

n	Average	Std.Dev.	Maximum	Minimum
6	9.833	1.0672	12.000	9.000

S.No	Sample ID	Time	Result	Scale	OR ° Arc	WLG	Lg.mm	Conc.	Temp.	Comment
1	006-chiral-A	09:02:12 PM	12.000	SR	0.012	589	100.00	0.100	18.8	
2	006-chiral-A	09:02:18 PM	9.000	SR	0.009	589	100.00	0.100	18.8	
3	006-chiral-A	09:02:24 PM	9.000	SR	0.009	589	100.00	0.100	18.8	
4	006-chiral-A	09:02:30 PM	9.000	SR	0.009	589	100.00	0.100	18.8	
5	006-chiral-A	09:02:36 PM	10.000	SR	0.010	589	100.00	0.100	18.8	
6	006-chiral-A	09:02:42 PM	10.000	SR	0.010	589	100.00	0.100	18.8	

Figure S20. Specific optical rotation of (+)-**6**.

LotID : 006-chiral-B
Set Temperature : OFF
Temp Corr : OFF

n	Average	Std.Dev.	Maximum	Minimum
6	-20.167	2.7335	-16.000	-24.000

S.No	Sample ID	Time	Result	Scale	OR ° Arc	WLG	Lg.mm	Conc.	Temp.	Comment
1	006-chiral-B	09:11:09 PM	-22.000	SR	-0.022	589	100.00	0.100	18.8	
2	006-chiral-B	09:11:15 PM	-22.000	SR	-0.022	589	100.00	0.100	18.8	
3	006-chiral-B	09:11:21 PM	-24.000	SR	-0.024	589	100.00	0.100	18.8	
4	006-chiral-B	09:11:27 PM	-19.000	SR	-0.019	589	100.00	0.100	18.8	
5	006-chiral-B	09:11:33 PM	-18.000	SR	-0.018	589	100.00	0.100	18.9	
6	006-chiral-B	09:11:39 PM	-16.000	SR	-0.016	589	100.00	0.100	18.9	

Figure S21. Specific optical rotation of (–)-**6**.

1.5 NMR, HR-ESI-MS, IR, and UV spectra of ocellatuperoxide A (1)

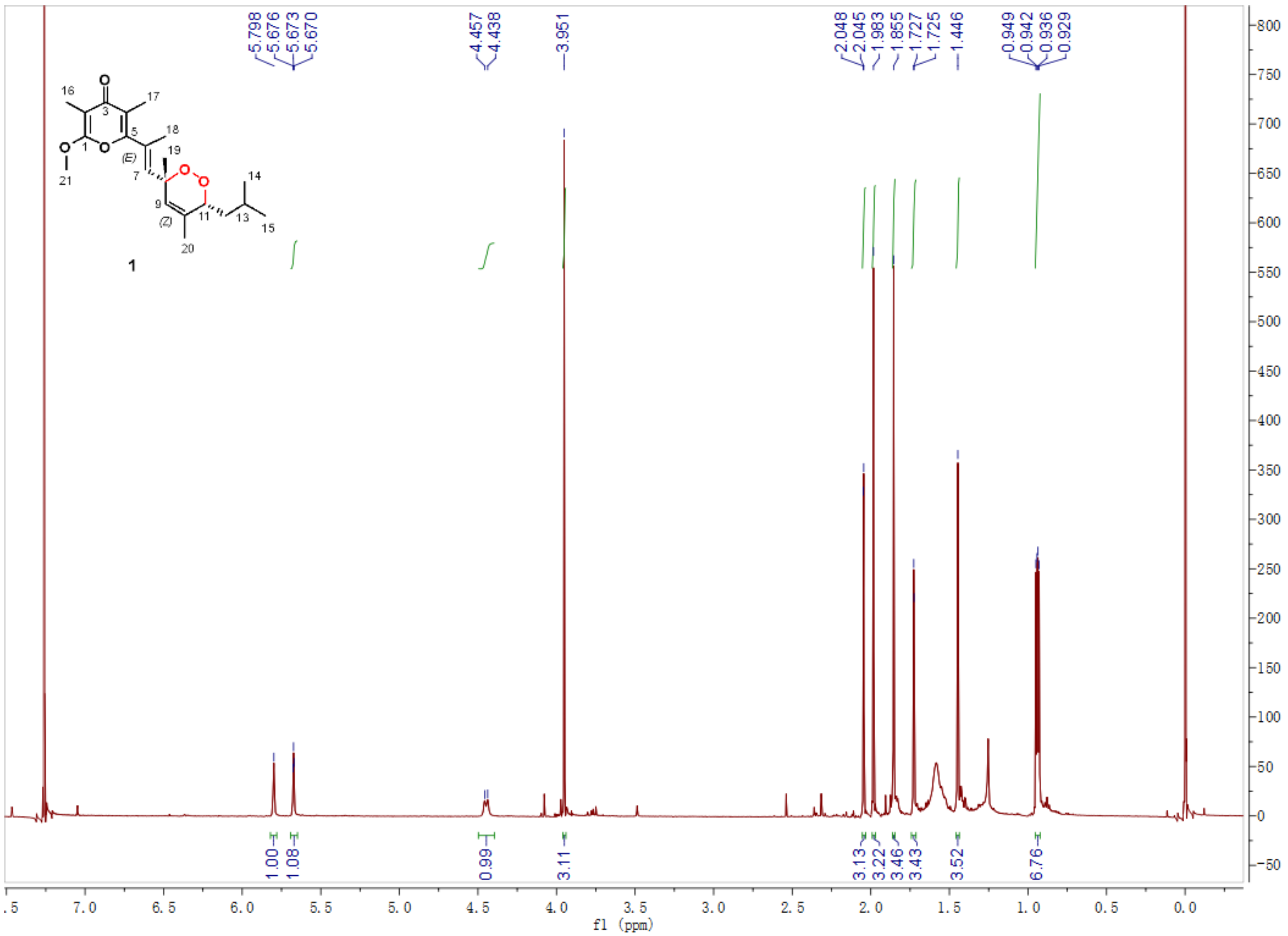

Figure S22. ^{1}H NMR spectrum (600 MHz) of **1** in $CDCl_3$.

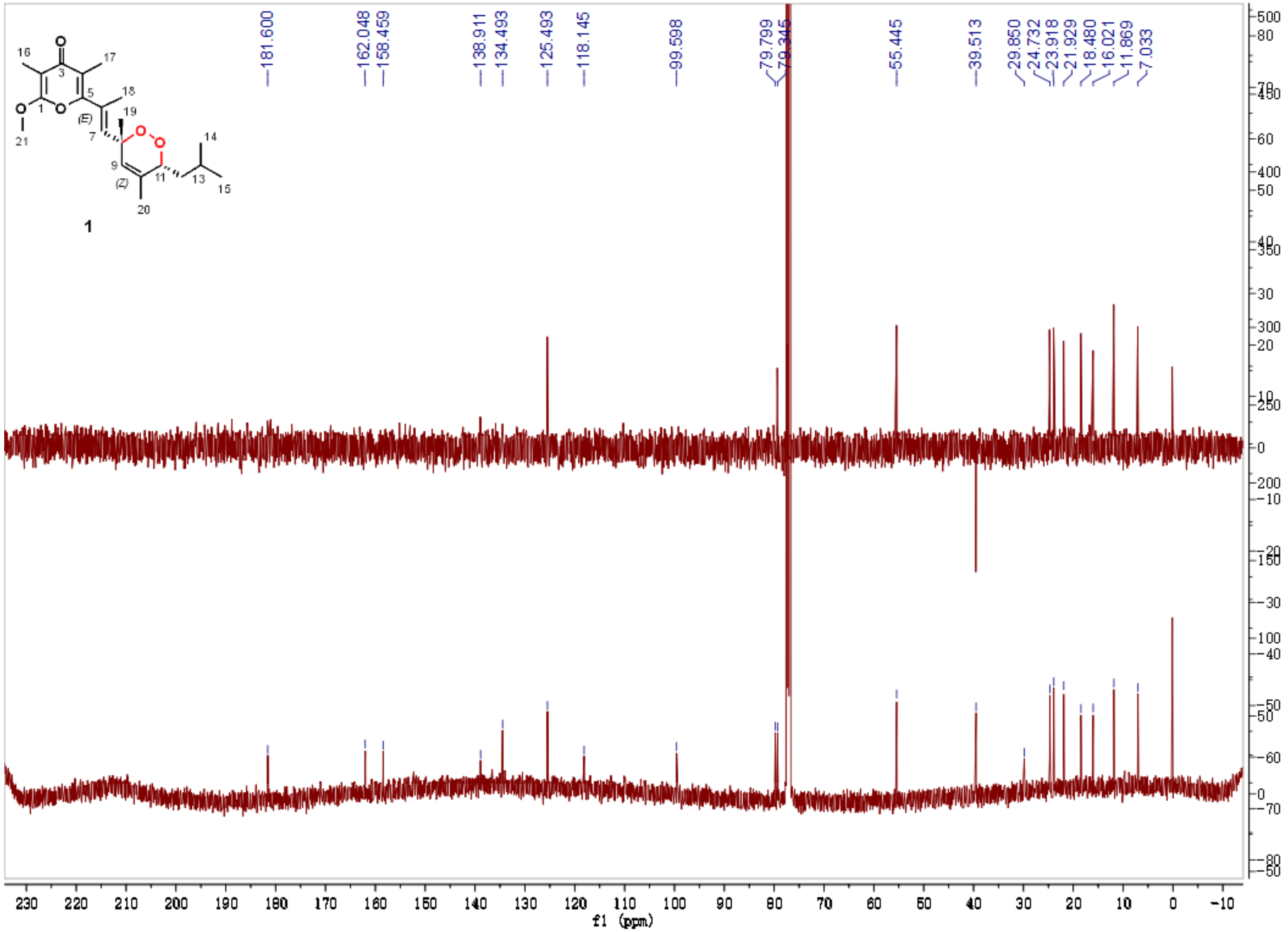

Figure S23. ^{13}C NMR spectrum (150 MHz) of **1** in $CDCl_3$.

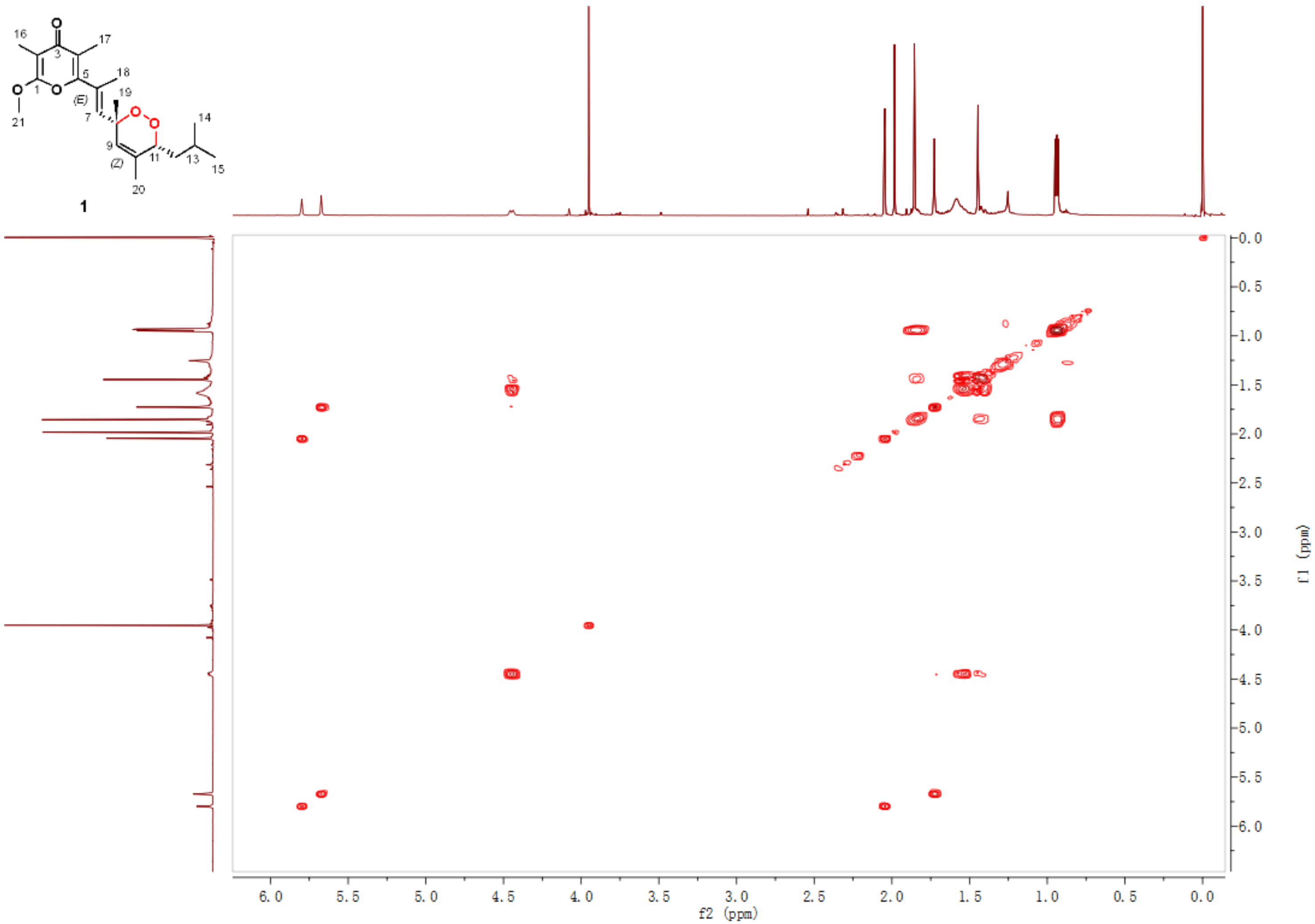

Figure S24. ^{1}H-^{1}H COSY spectrum (600 MHz) of **1** in $CDCl_3$.

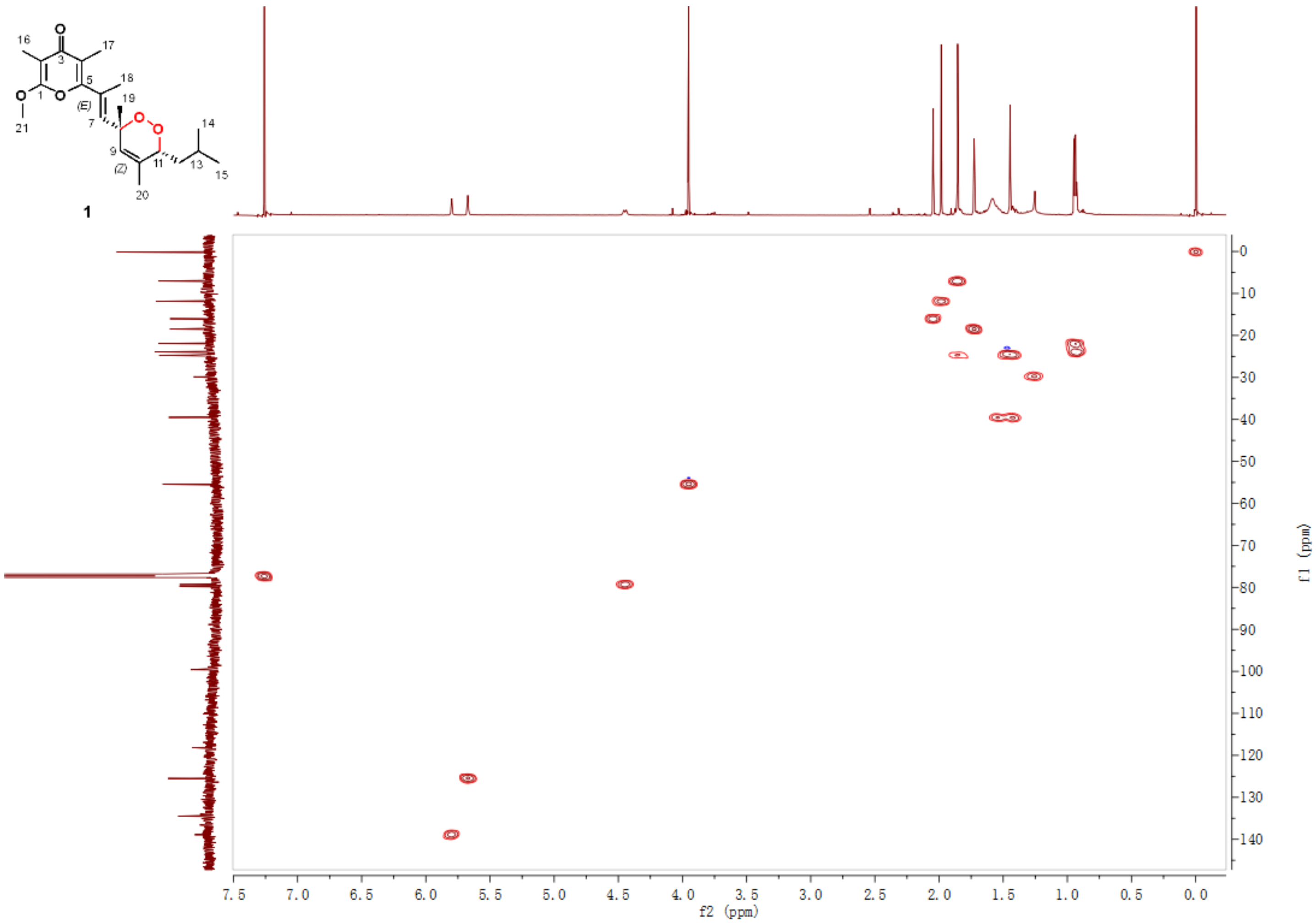

Figure S25. HSQC spectrum (600 MHz) of **1** in $CDCl_3$.

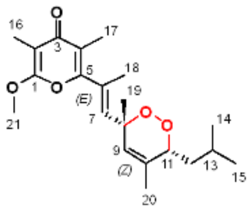

1

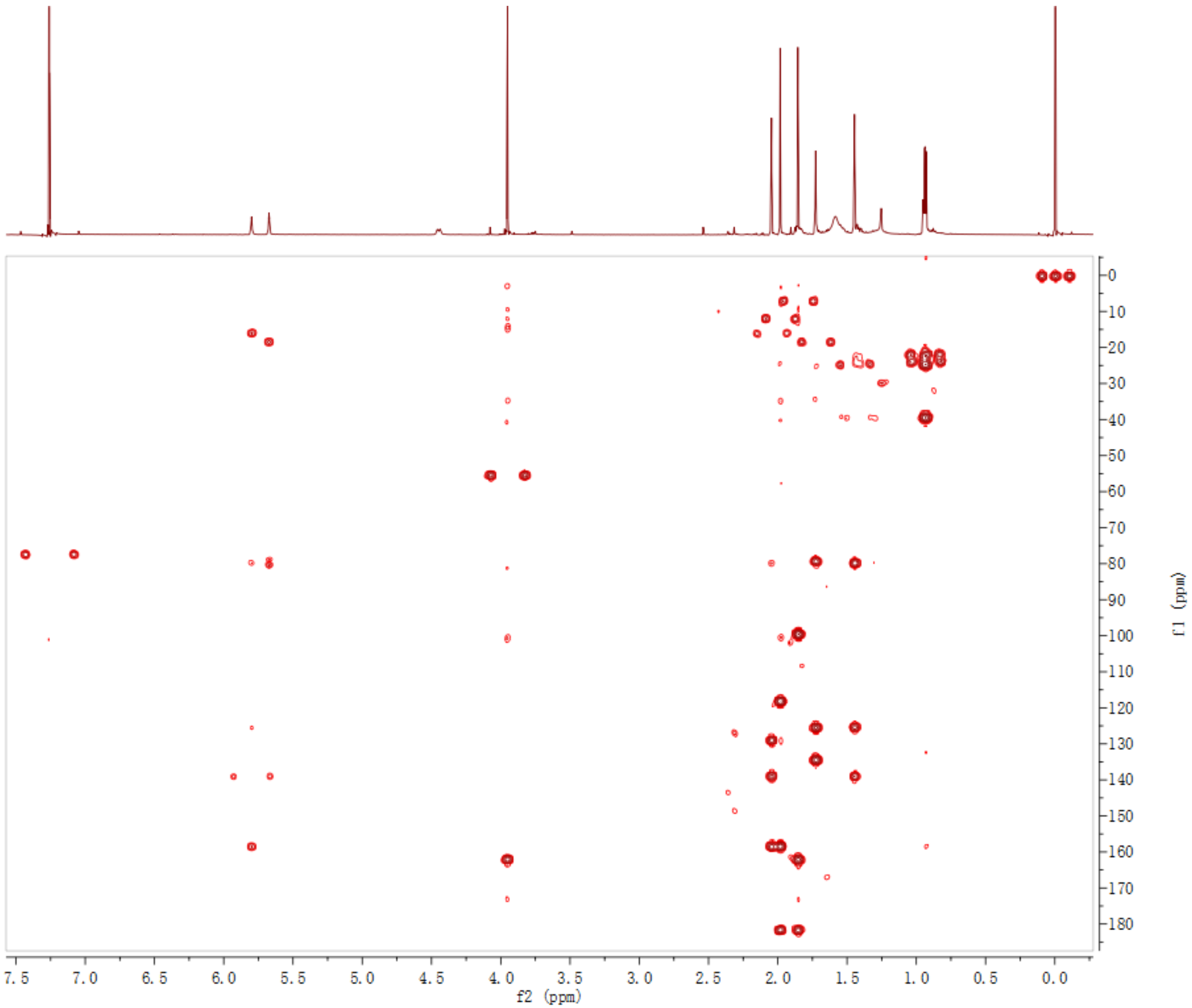

Figure S26. HMBC spectrum (600 MHz) of **1** in $CDCl_3$.

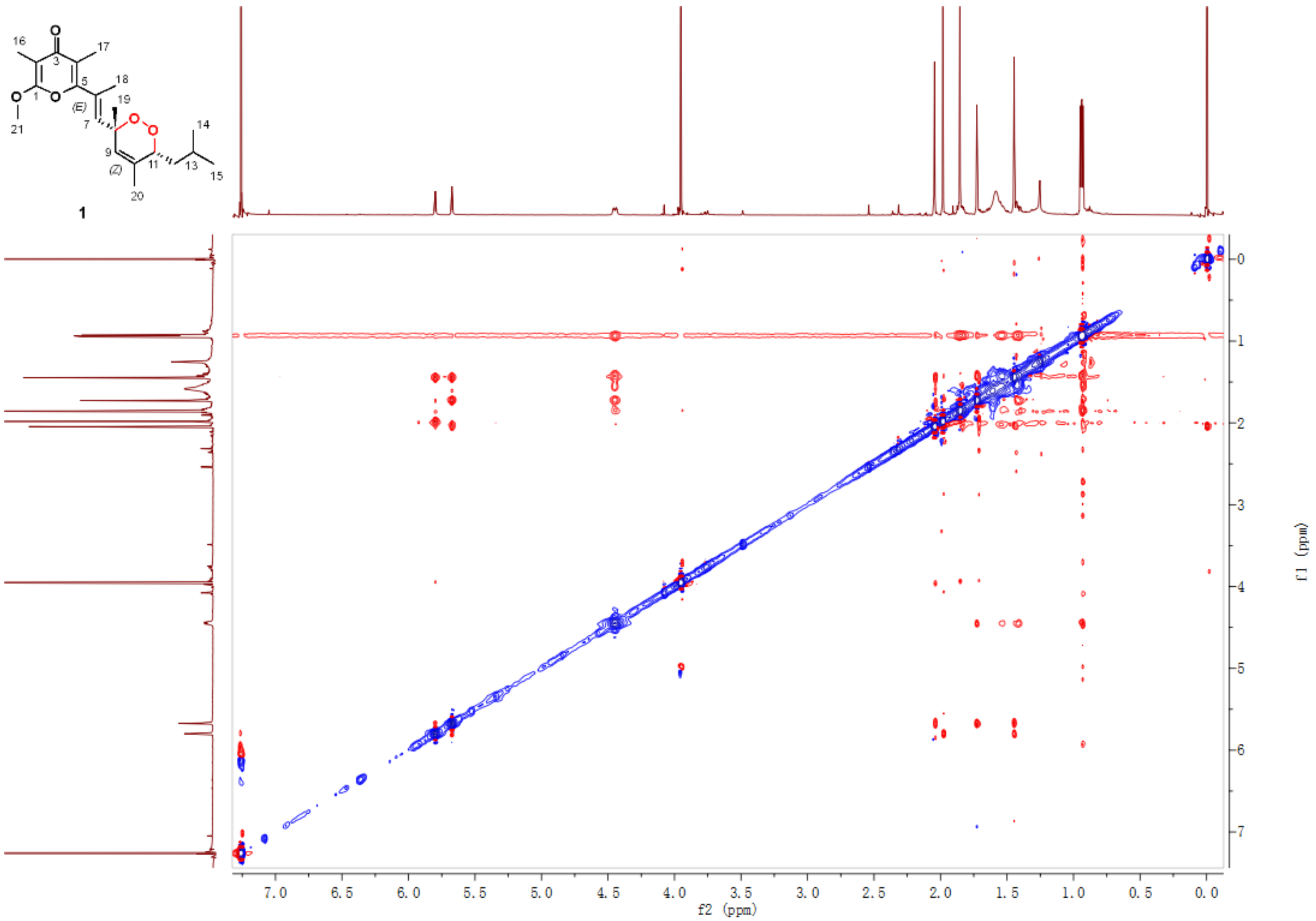

Figure S27. NOESY spectrum (600 MHz) of **1** in $CDCl_3$.

User Spectra

Fragmentor Voltage	Collision Energy	Ionization Mode
135	0	ESI

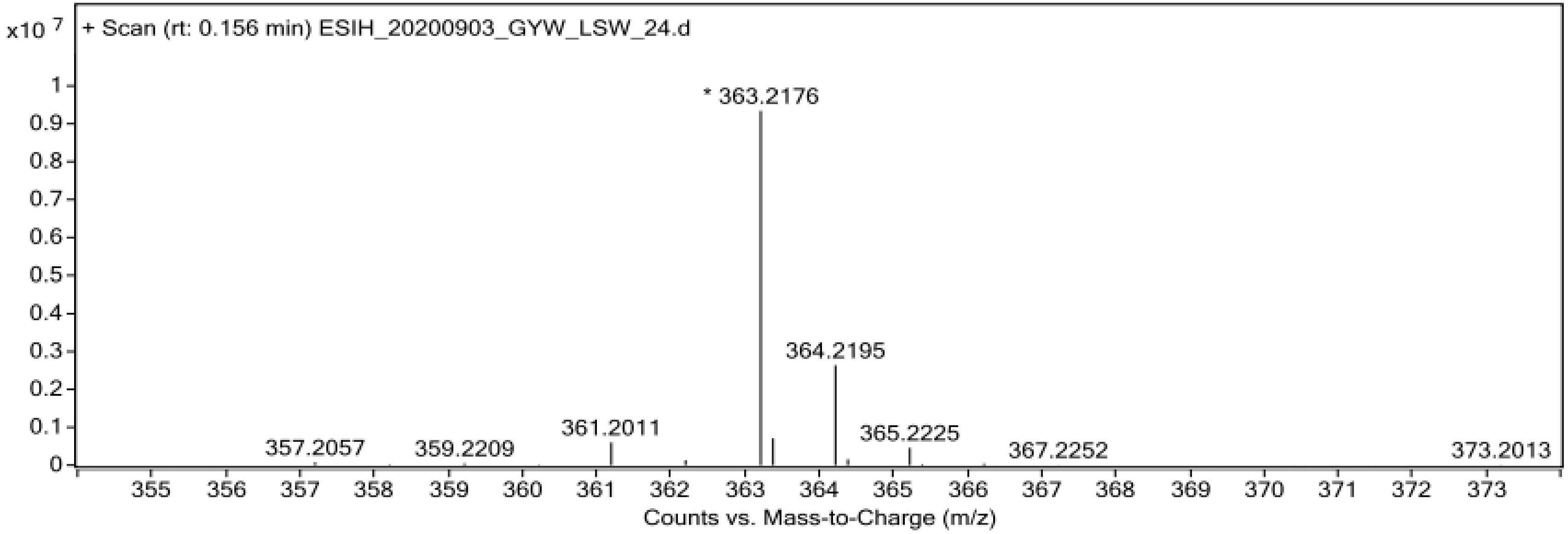

Formula Calculator Results

m/z	Calc m/z	Diff (mDa)	Diff (ppm)	Ion Formula	Ion
363.2176	363.2166	-0.96	-2.63	C21 H31 O5	(M+H)+

Figure S28. HR-ESI-MS (positive mode) spectrum of **1**.

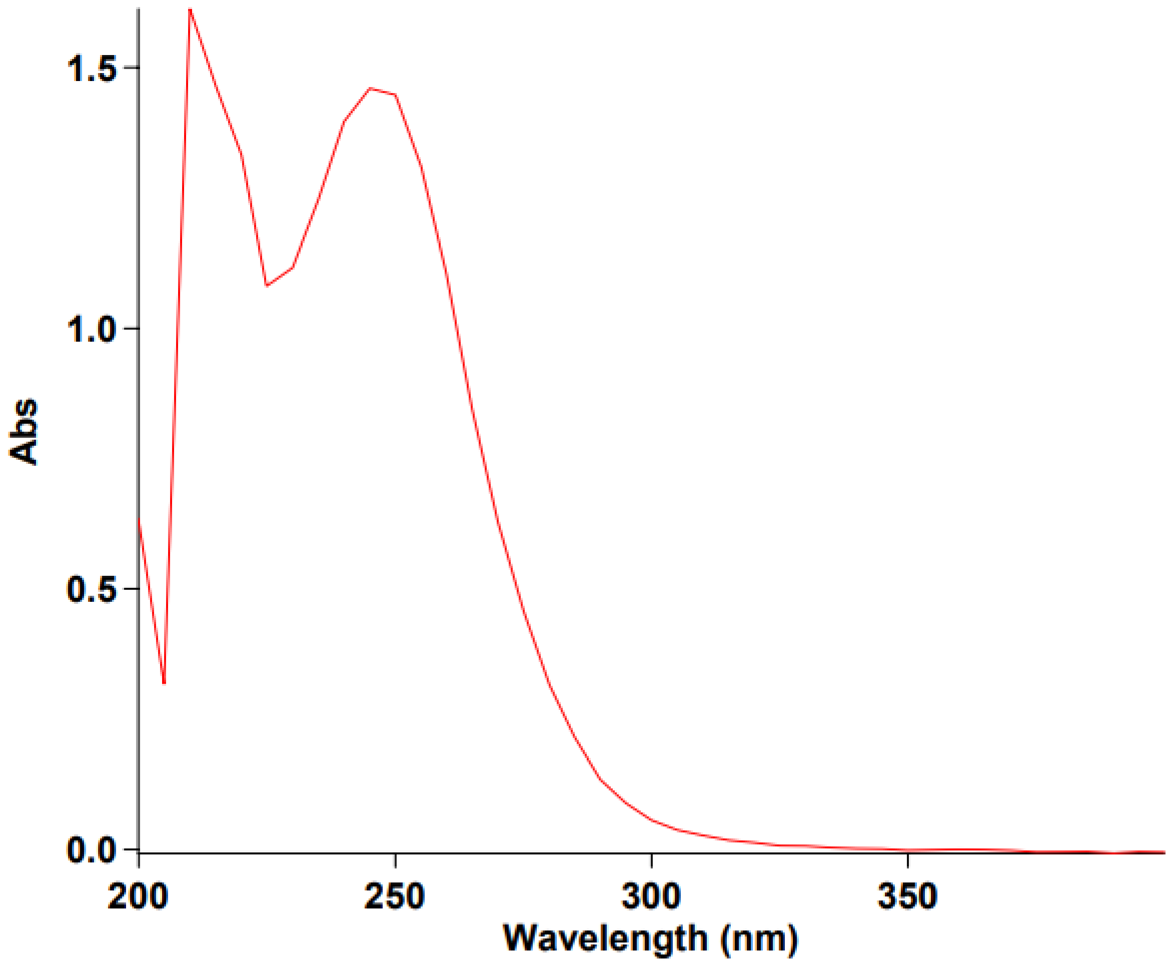

Figure S29. UV spectrum of **1**.

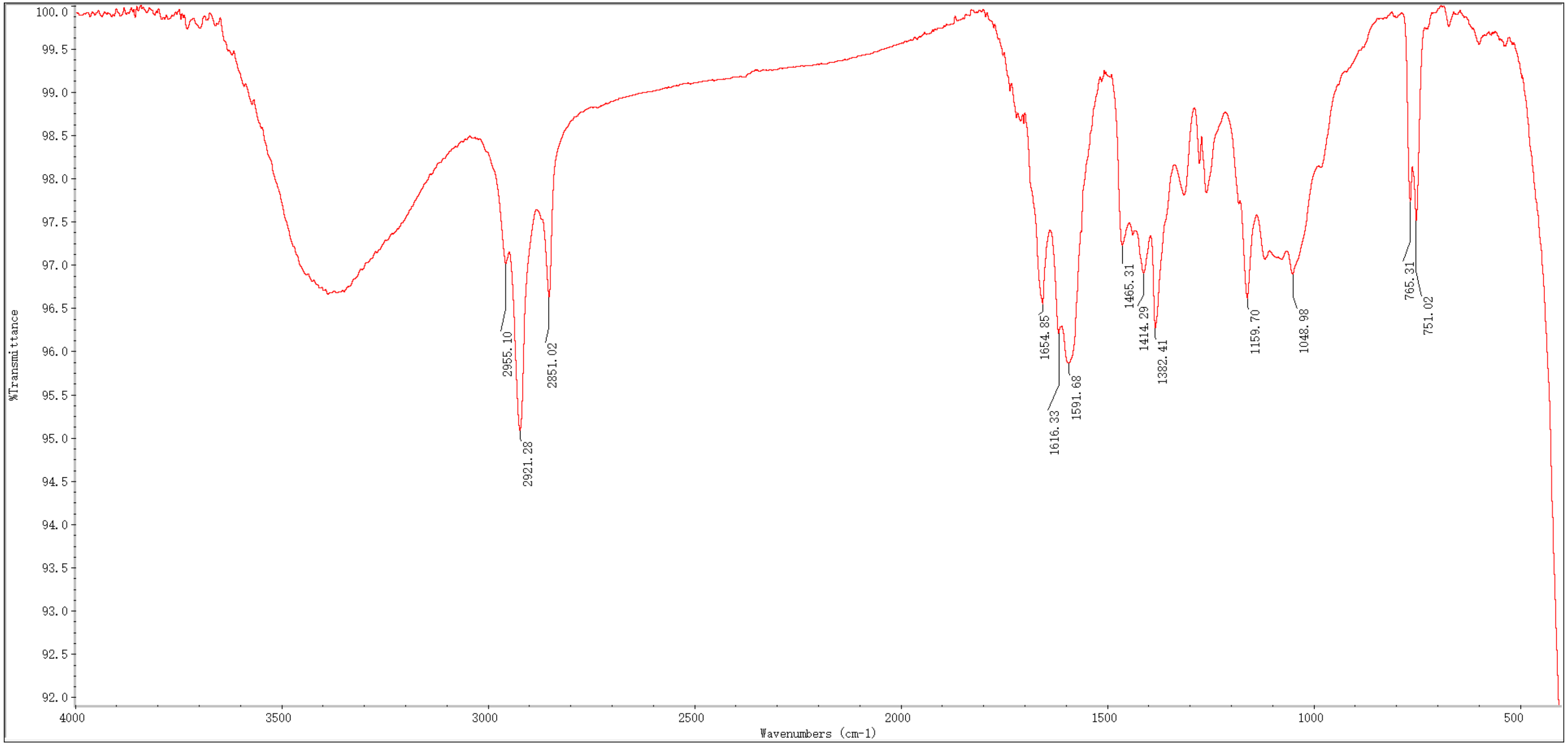

Figure S30. IR spectrum of **1**.

1.6 NMR, HR-ESI-MS, IR, and UV spectra of ocellatuperoxide B (2)

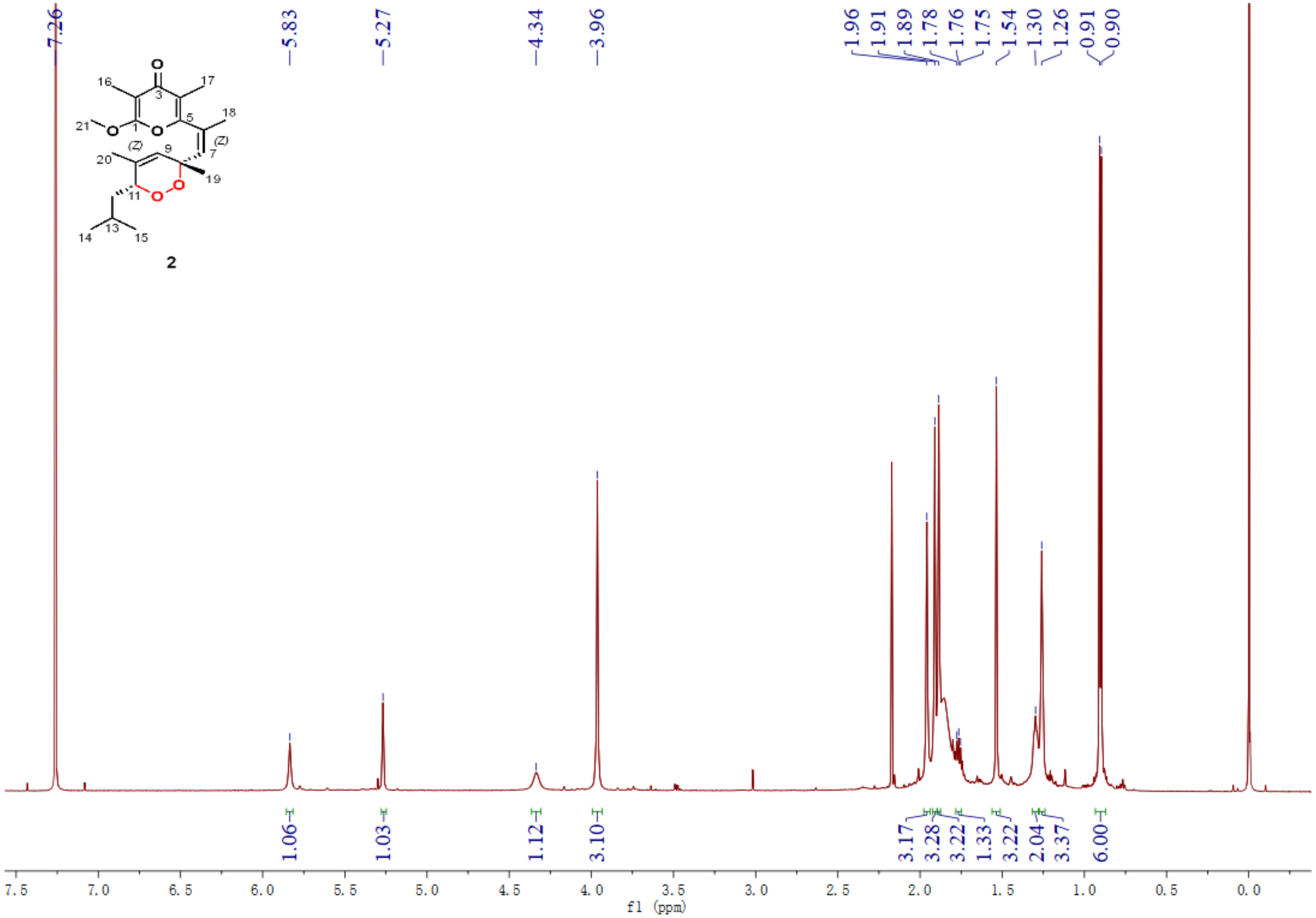

Figure S31. ^{1}H NMR spectrum (600 MHz) of **2** in $CDCl_3$.

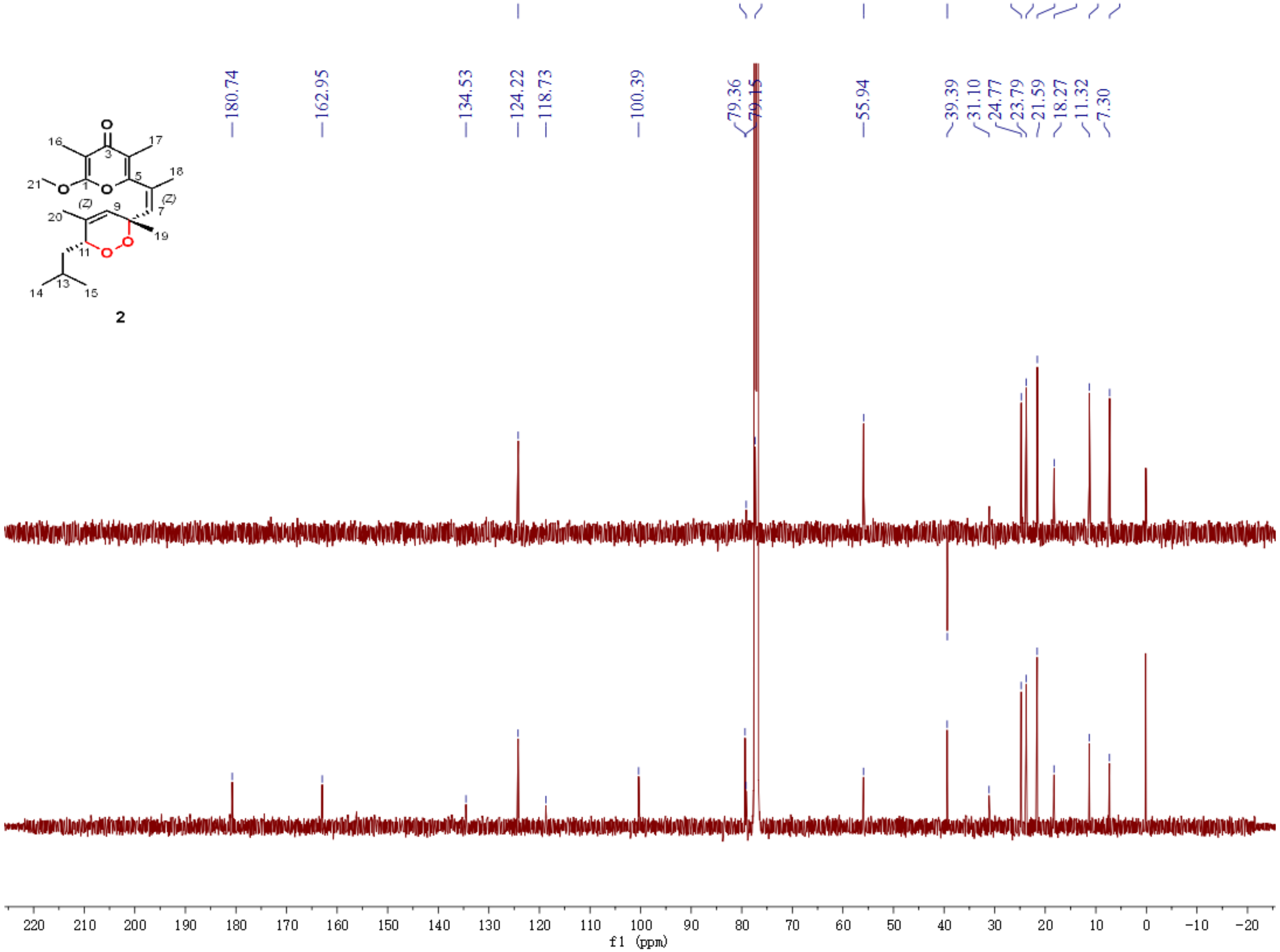

Figure S32. ^{13}C NMR spectrum (150 MHz) of **2** in $CDCl_3$.

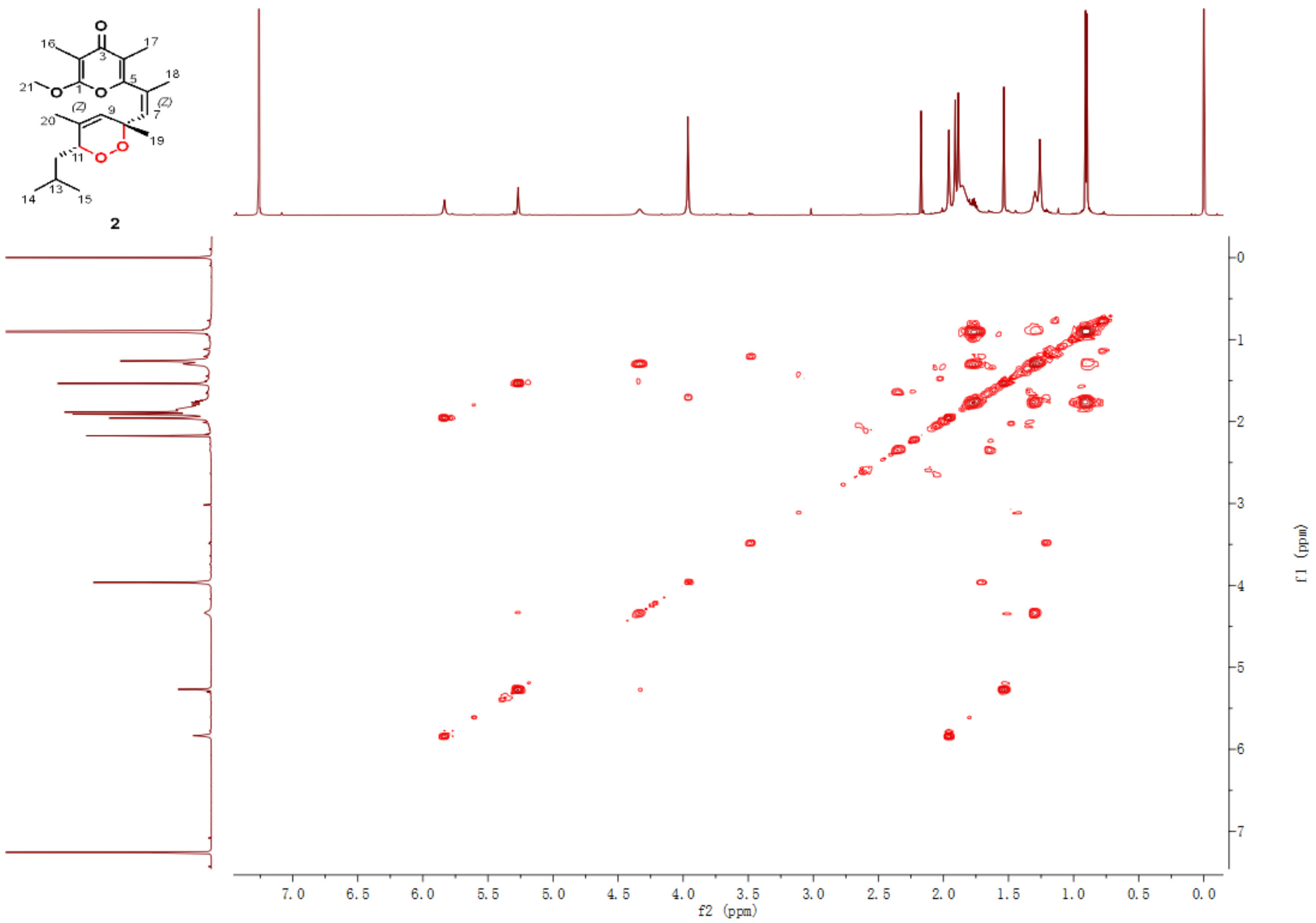

Figure S33. ^{1}H-^{1}H COSY spectrum (600 MHz) of **2** in $CDCl_3$.

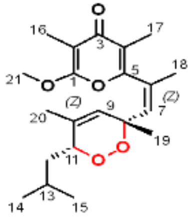

2

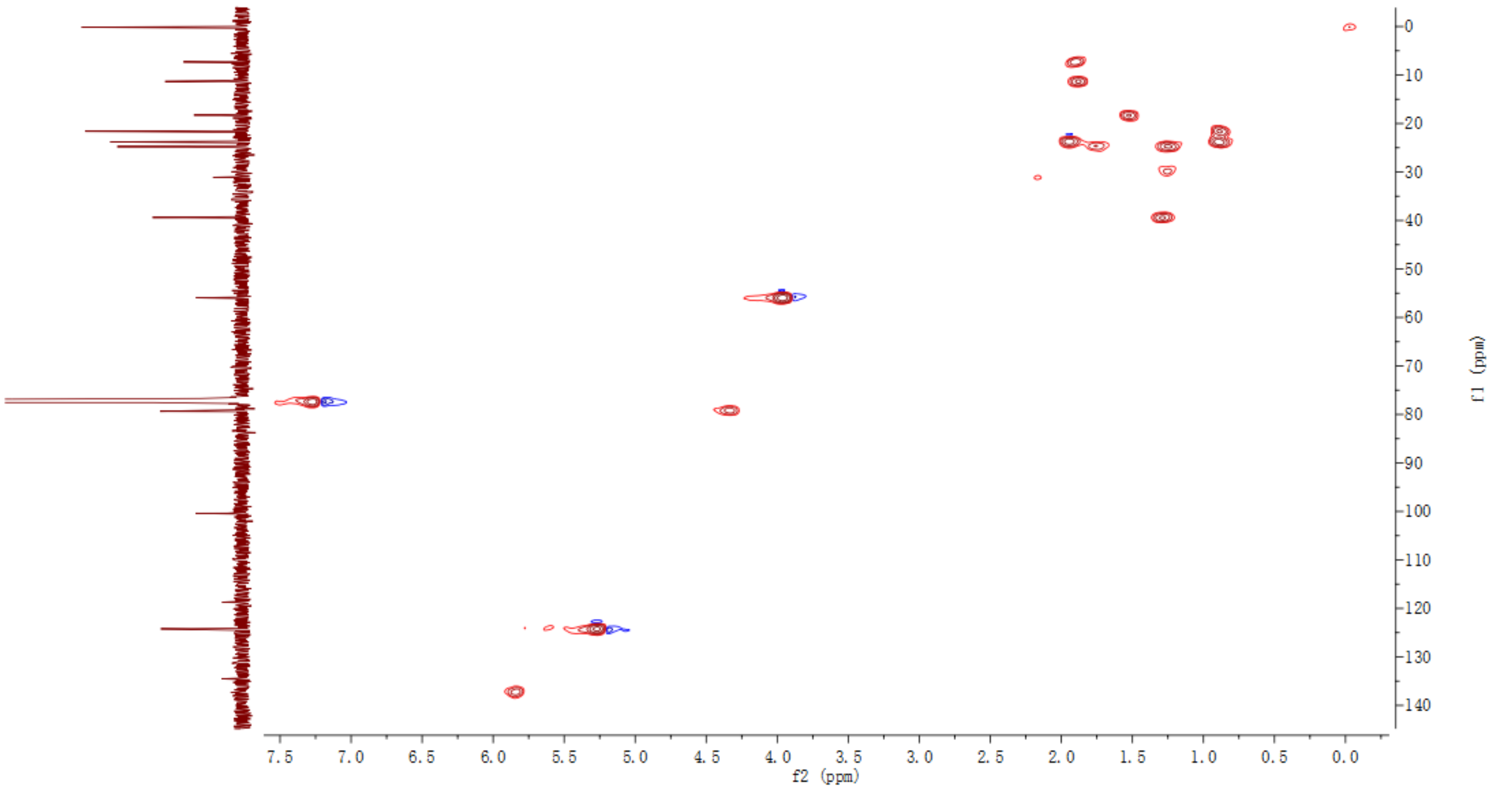

Figure S34. HSQC spectrum (600 MHz) of **2** in $CDCl_3$.

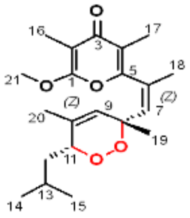

2

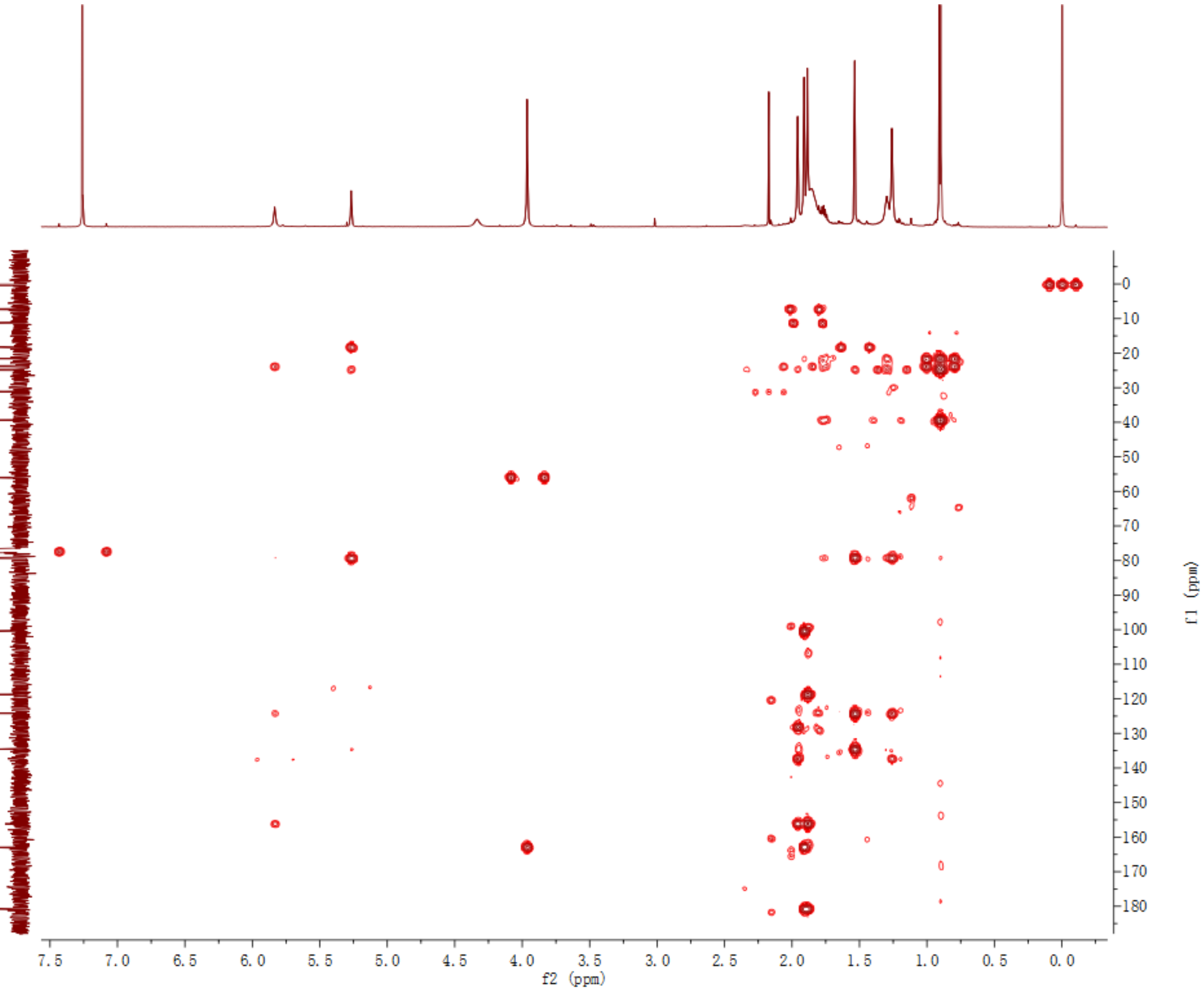

Figure S35. HMBC spectrum (600 MHz) of **2** in $CDCl_3$.

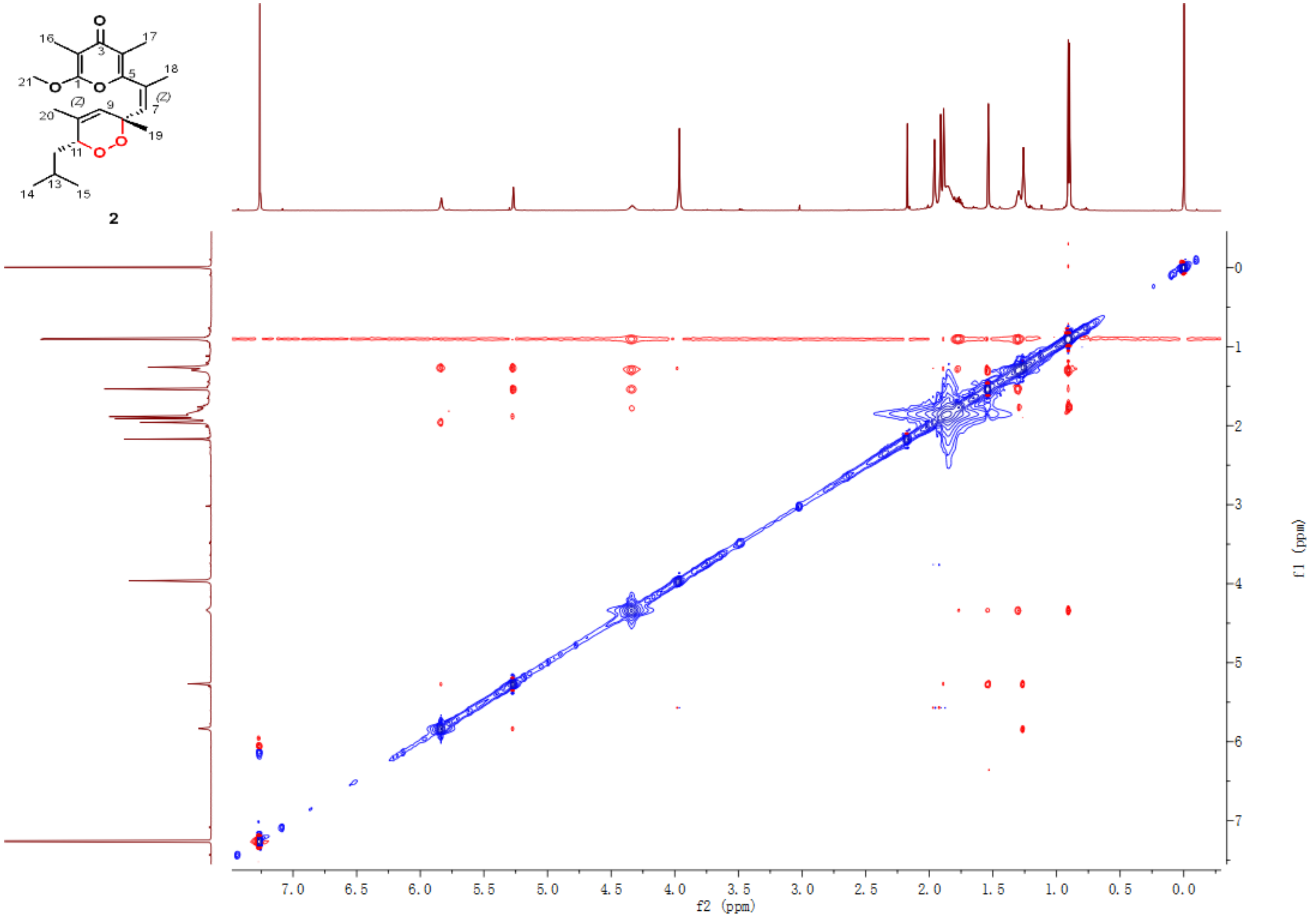

Figure S36. NOESY spectrum (600 MHz) of **2** in $CDCl_3$.

User Spectra

Fragmentor Voltage	Collision Energy	Ionization Mode
135	0	ESI

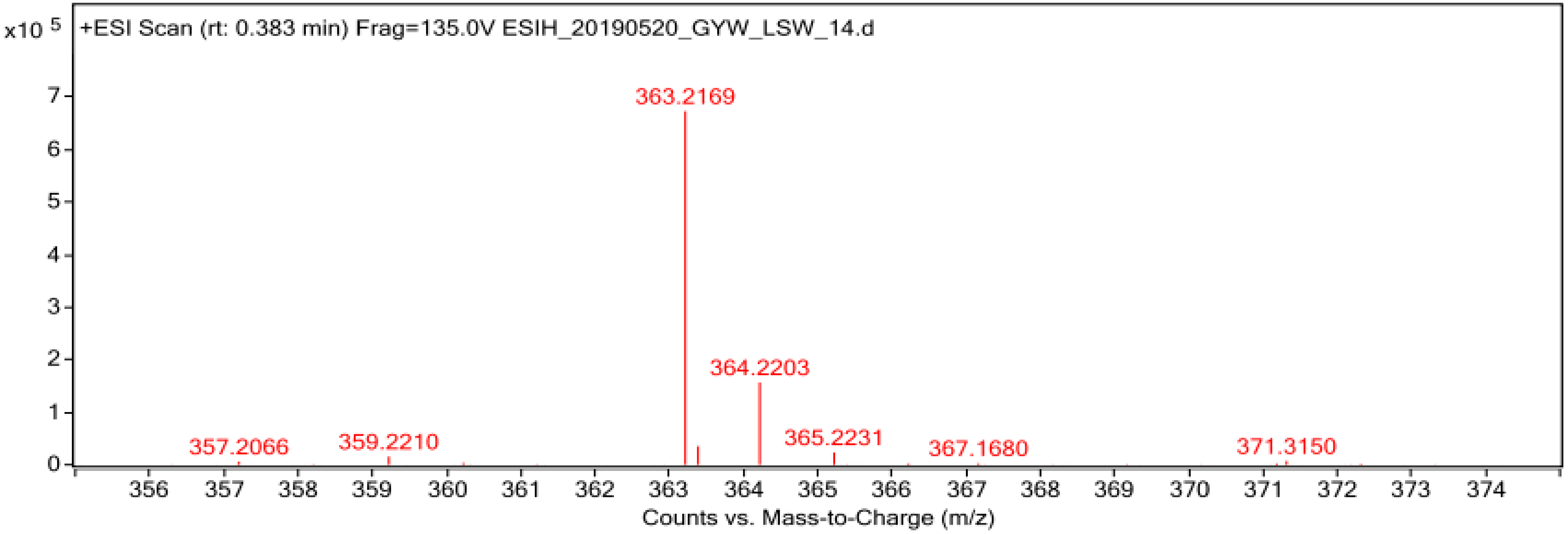

Formula Calculator Results

m/z	Calc m/z	Diff (mDa)	Diff (ppm)	Ion Formula	Ion
363.2169	363.2166	-0.27	-0.75	C21 H31 O5	(M+H)+

Figure S37. HR-ESI-MS (positive mode) spectrum of **2**.

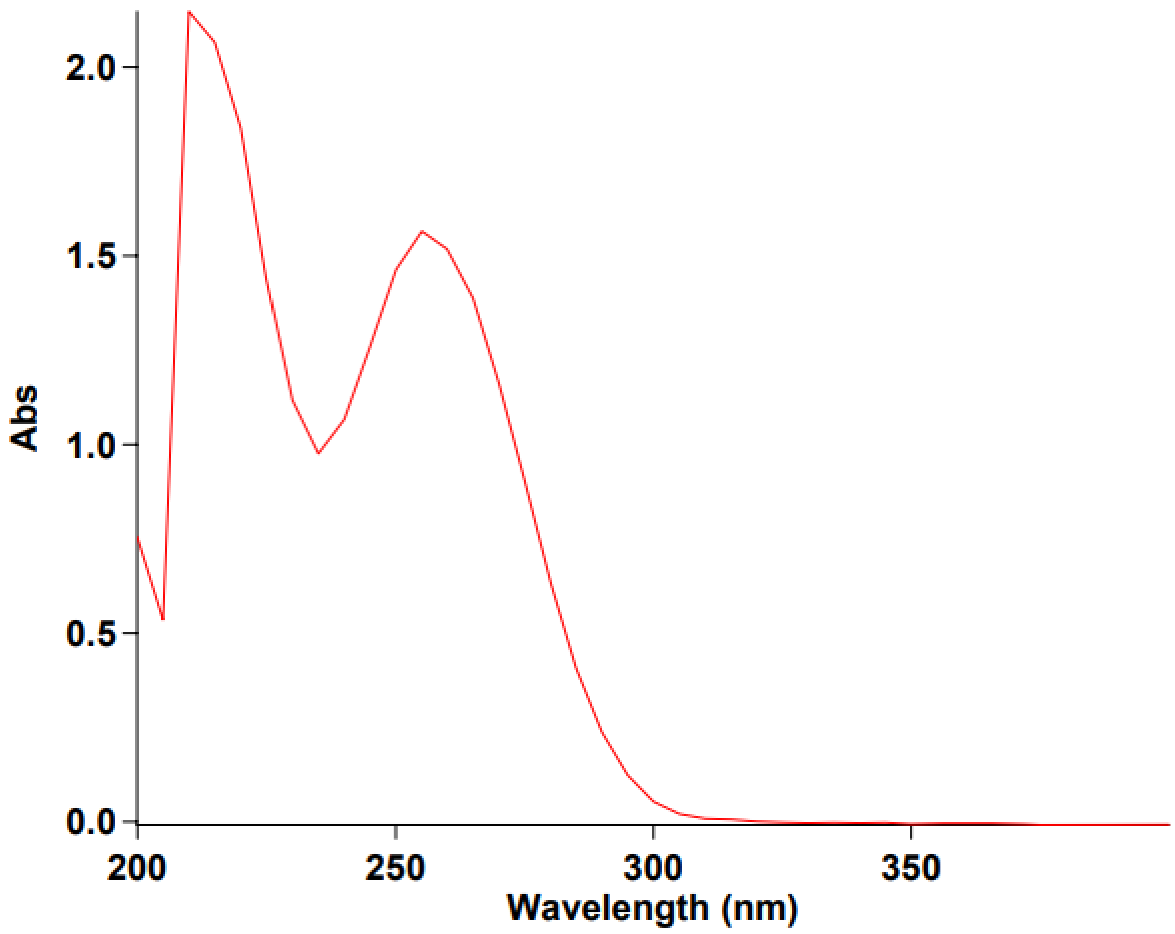

Figure S38. UV spectrum of **2**.

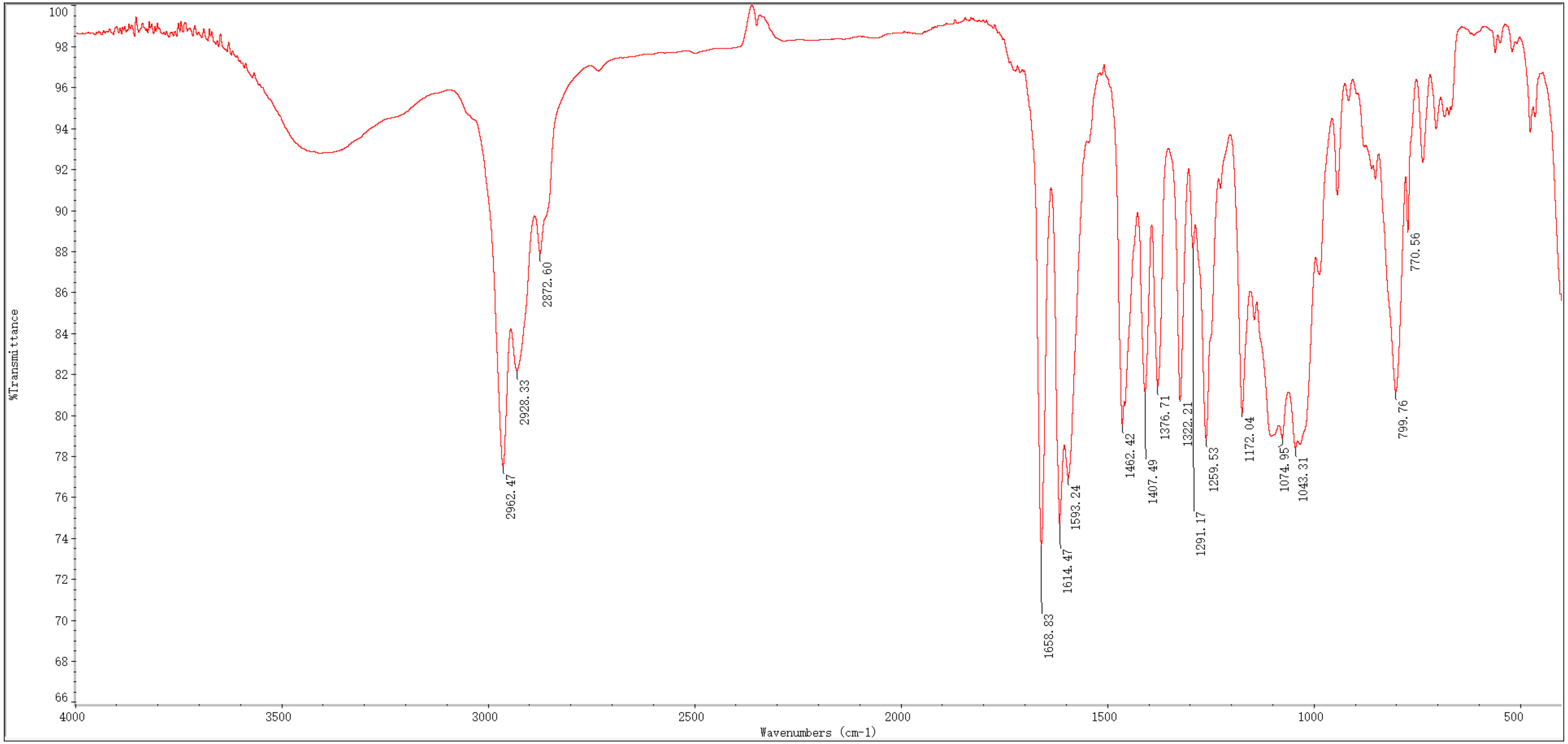

Figure S39. IR spectrum of **2**.

1.7 NMR, HR-ESI-MS, IR, and UV spectra of ocellatuperoxide C (3)

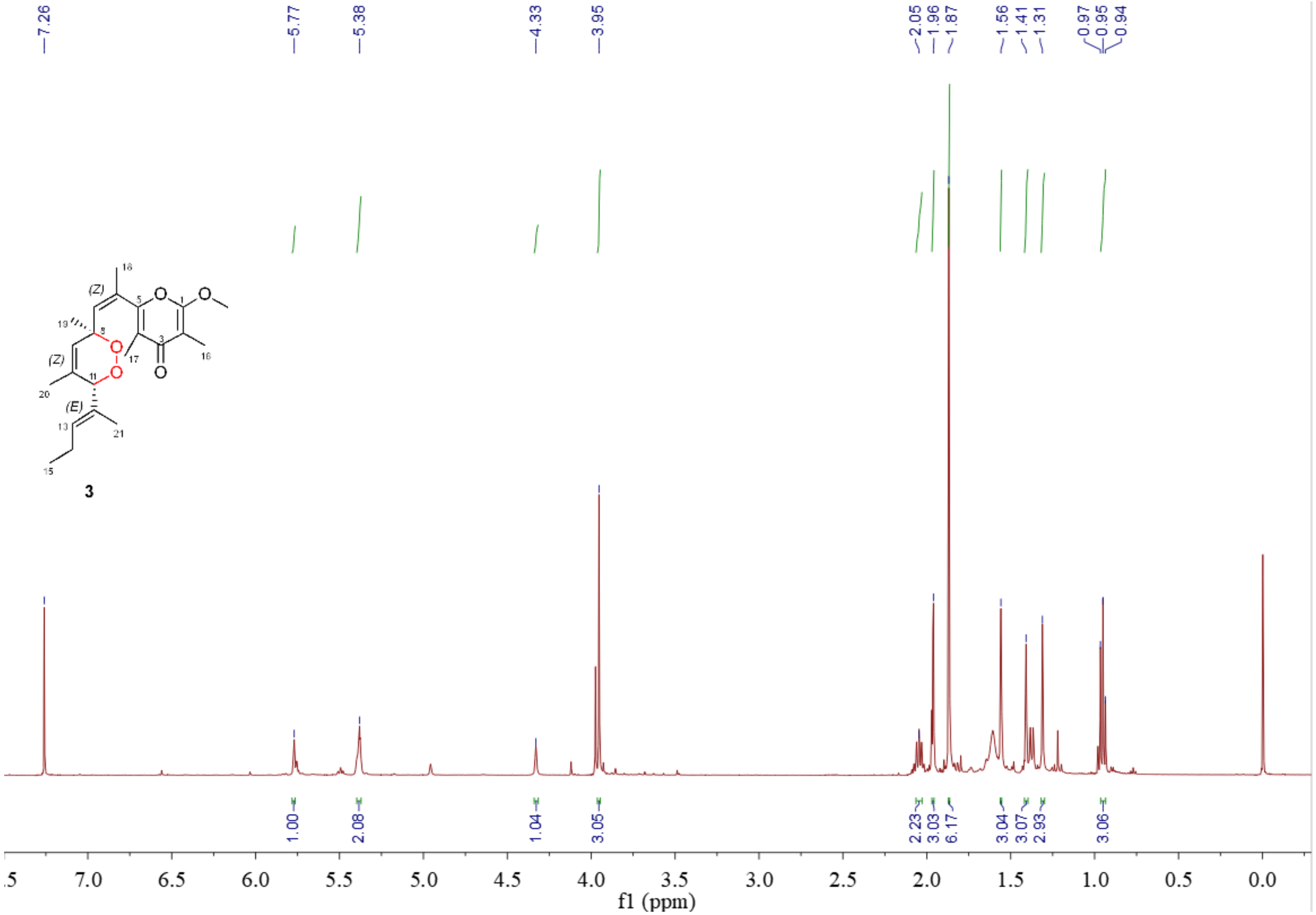

Figure S40. ^{1}H NMR spectrum (600 MHz) of **3** in $CDCl_3$.

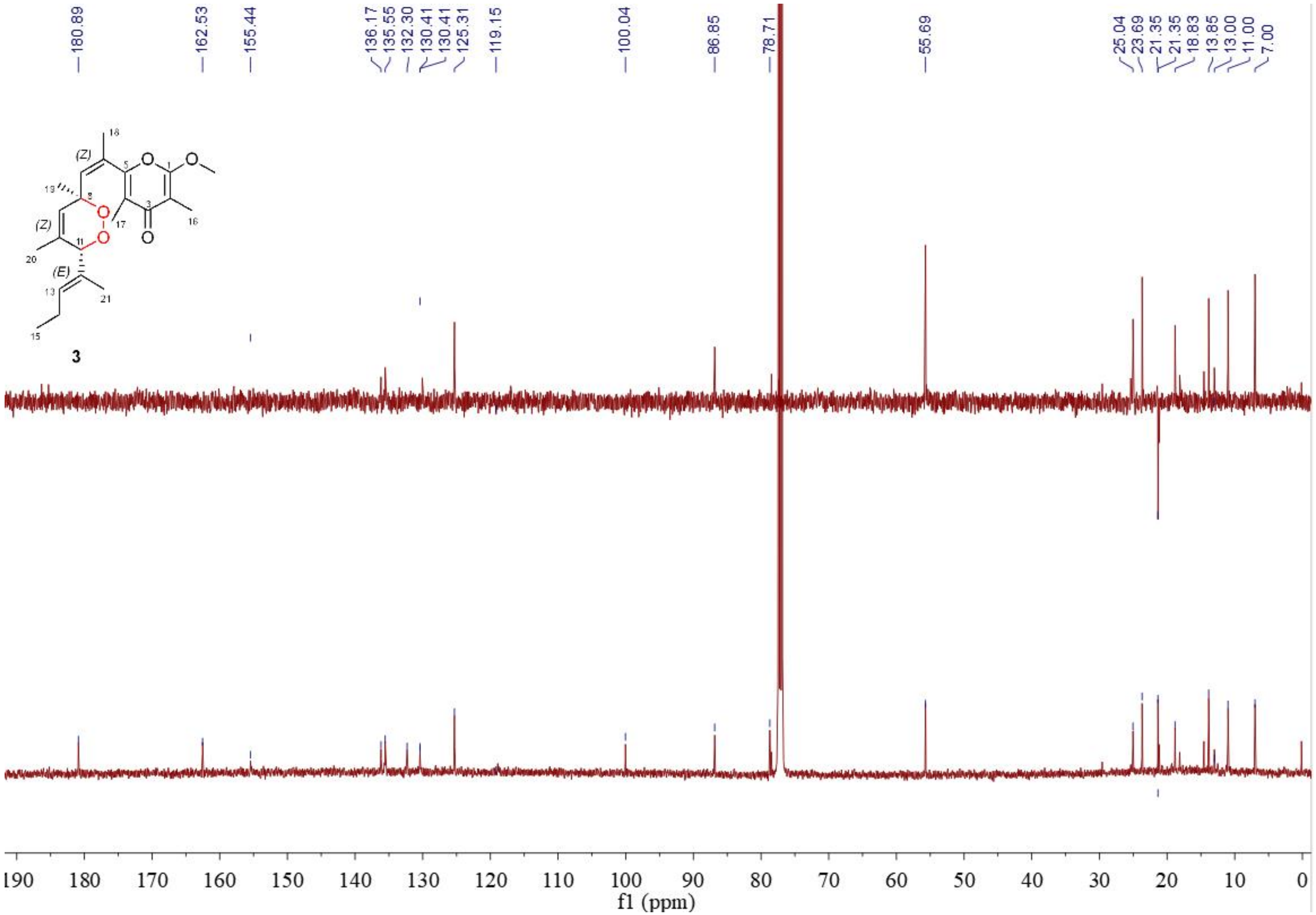

Figure S41. ^{13}C NMR spectrum (150 MHz) of **3** in $CDCl_3$.

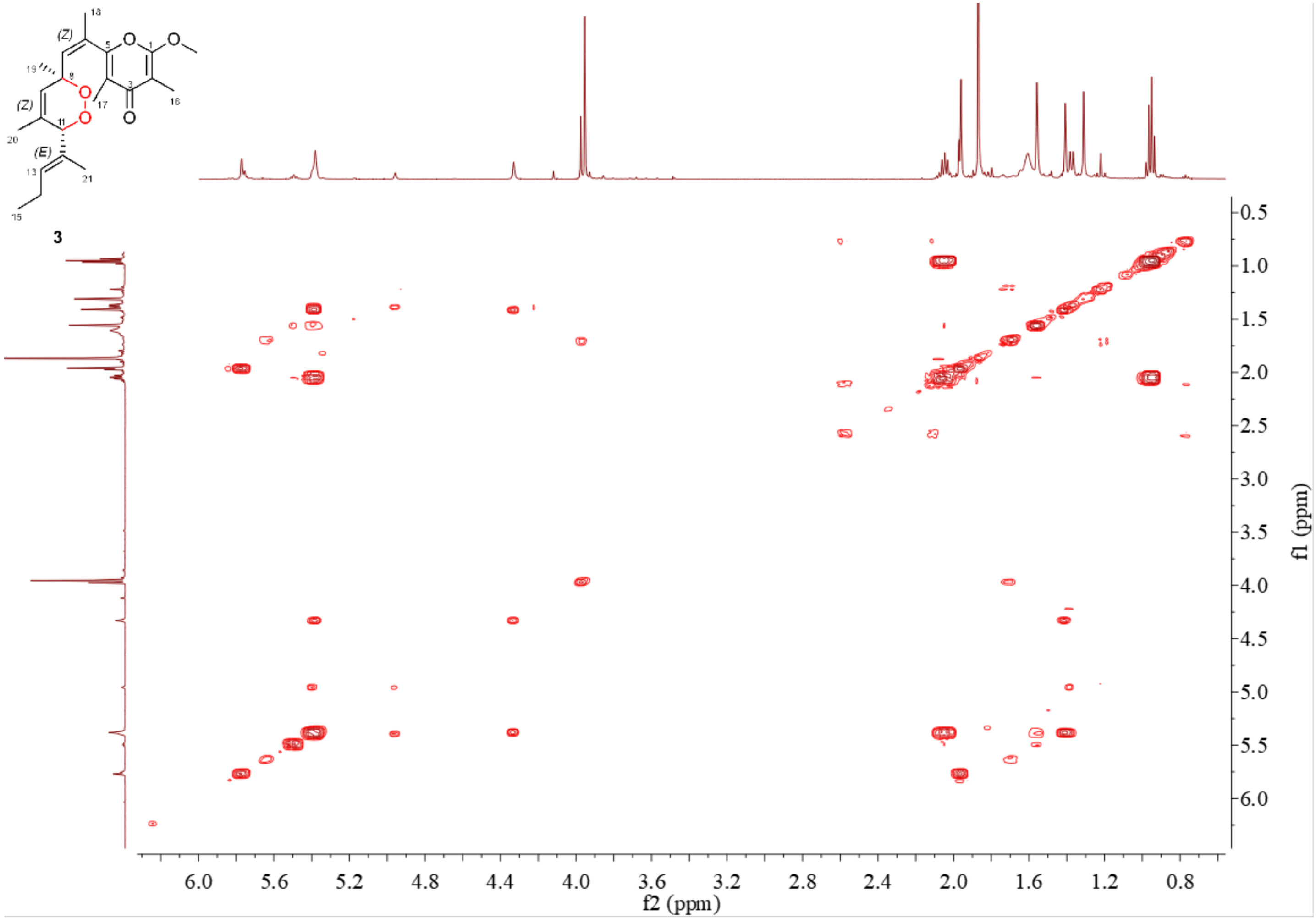

Figure S42. ^{1}H-^{1}H COSY spectrum (600 MHz) of **3** in $CDCl_3$.

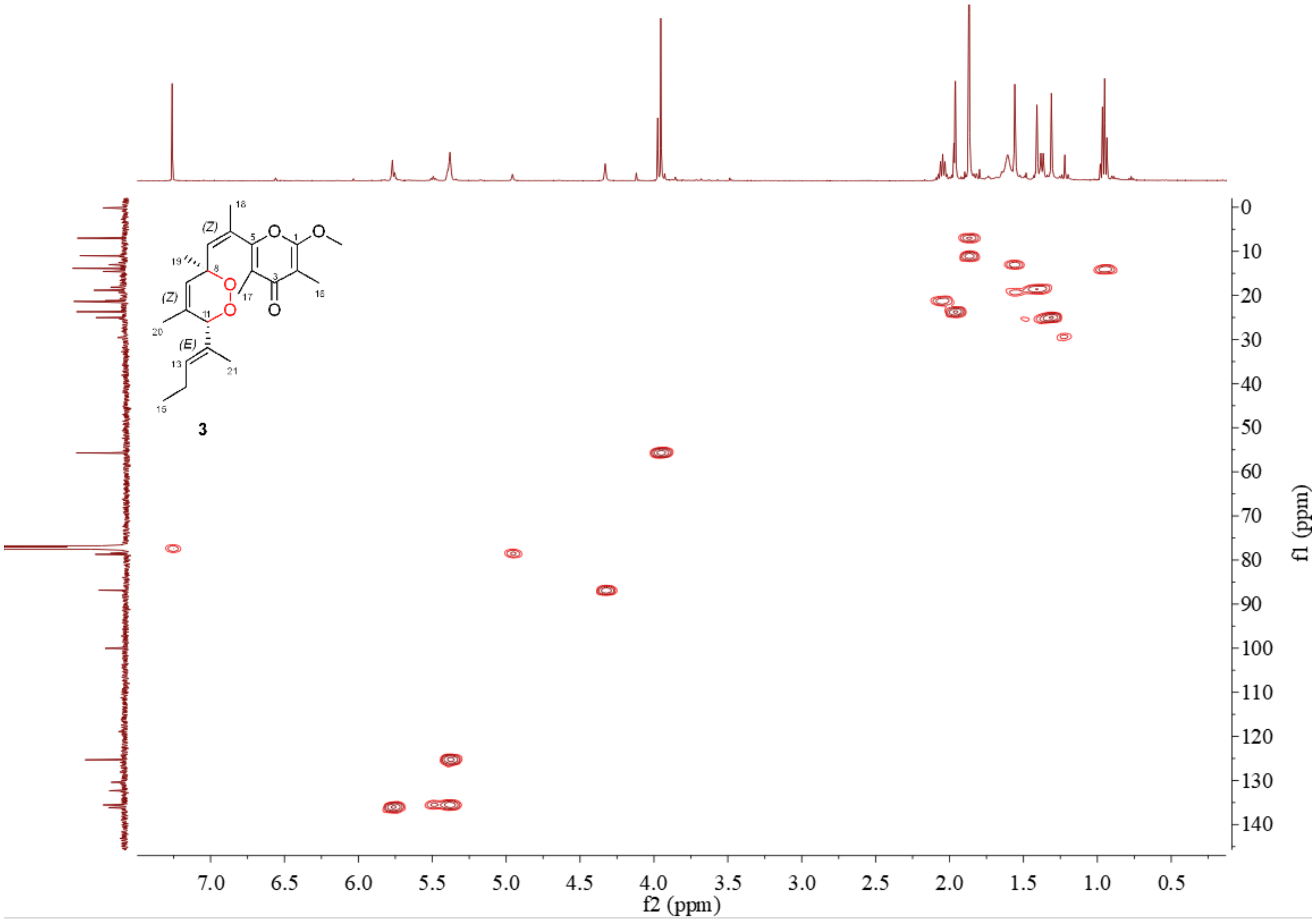

Figure S43. HSQC spectrum (600 MHz) of **3** in $CDCl_3$.

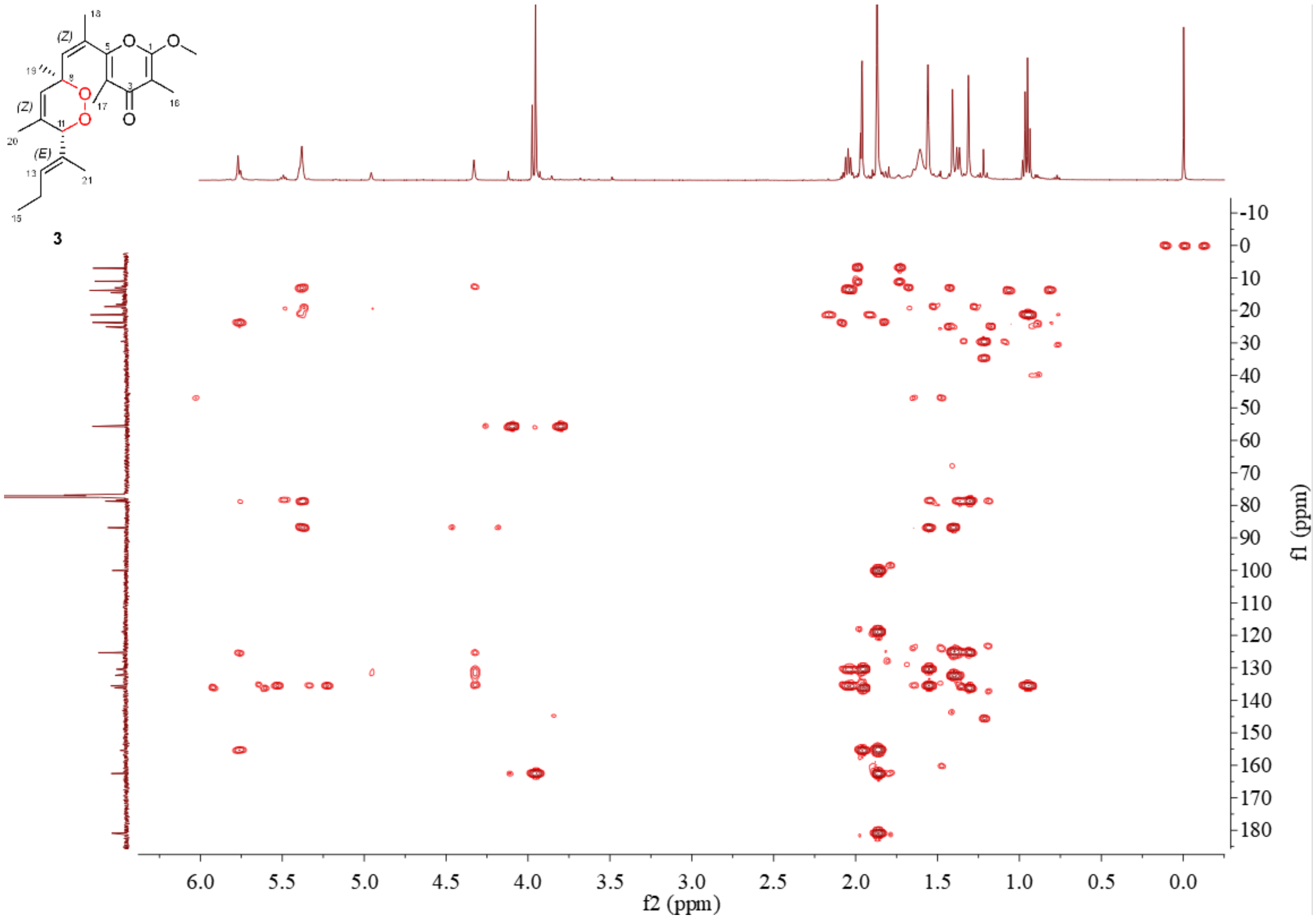

Figure S44. HMBC spectrum (600 MHz) of **3** in $CDCl_3$.

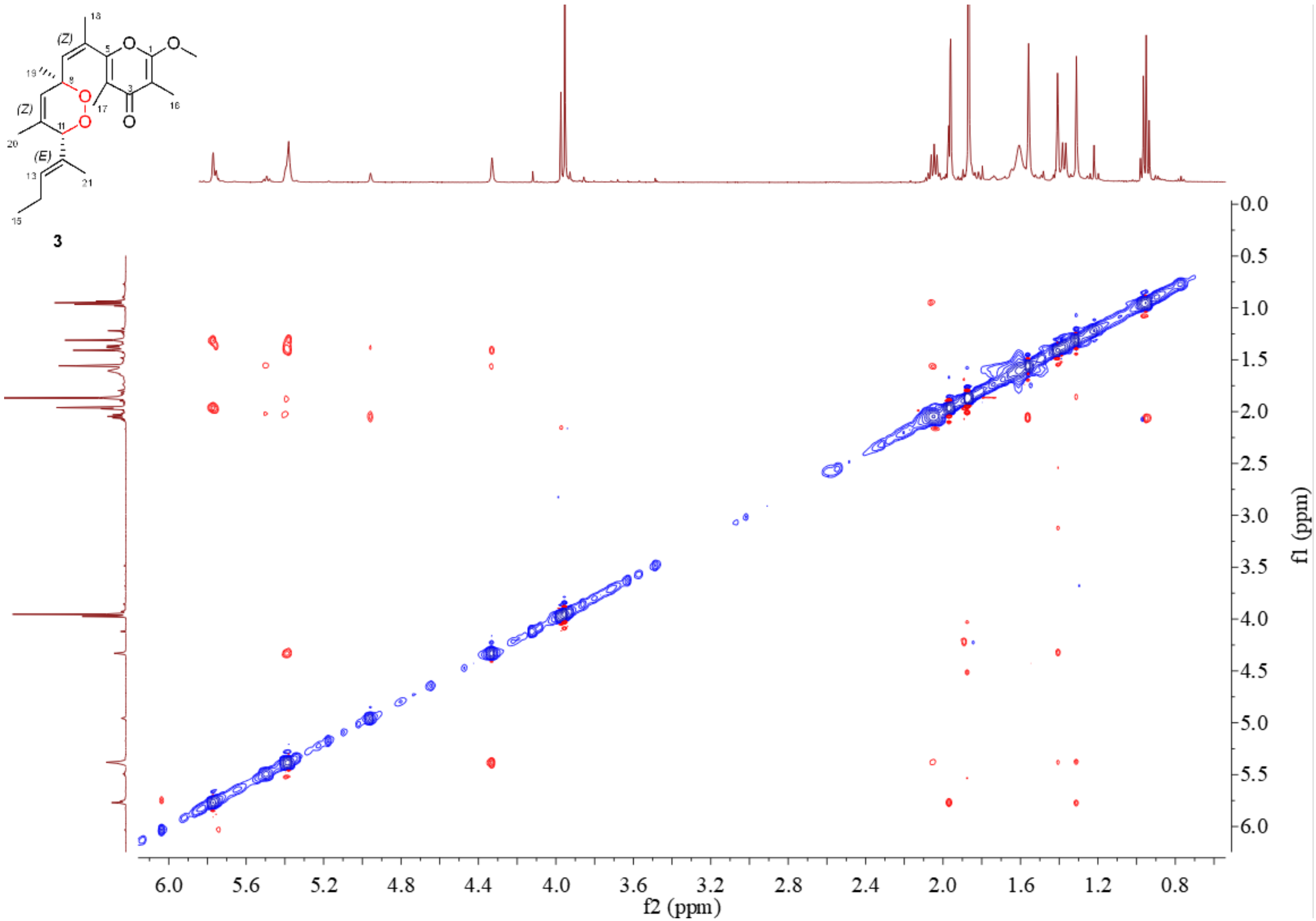

Figure S45. NOESY spectrum (600 MHz) of **3** in $CDCl_3$.

User Spectra

Fragmentor Voltage	Collision Energy	Ionization Mode
175	0	ESI

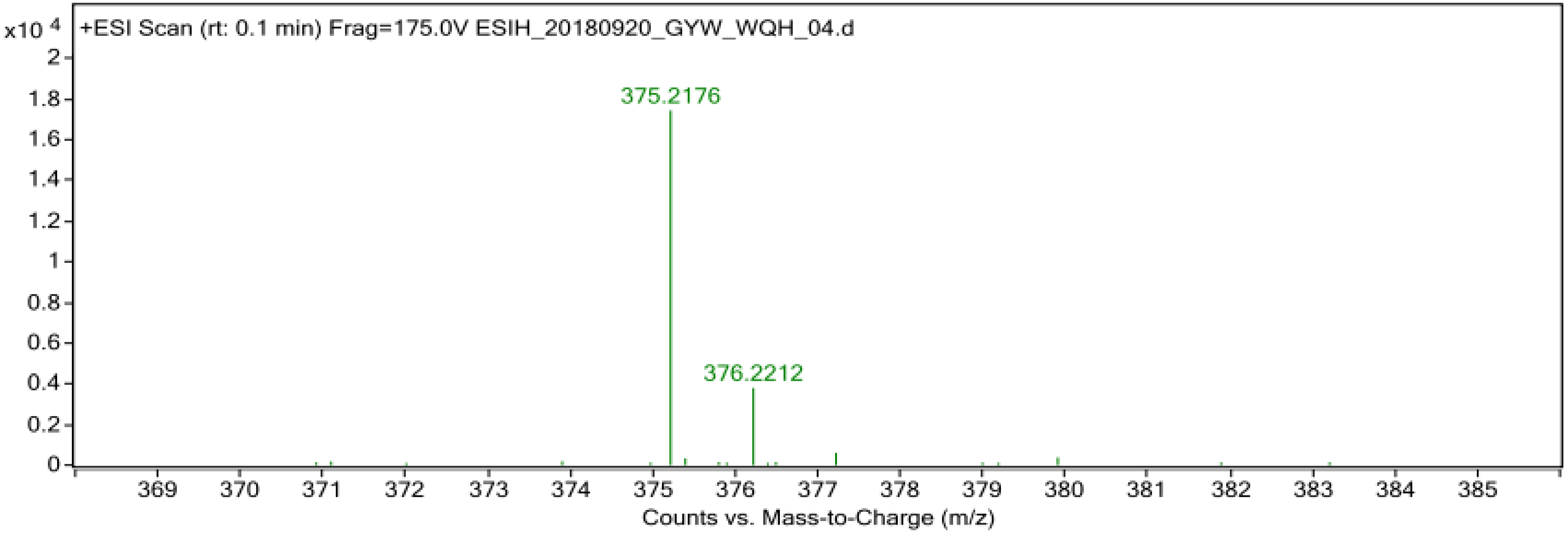

Formula Calculator Results

m/z	Calc m/z	Diff (mDa)	Diff (ppm)	Ion Formula	Ion
375.2176	375.2166	-1.02	-2.71	C22 H31 O5	(M+H)+

Figure S46. HR-ESI-MS (positive mode) spectrum of **3**.

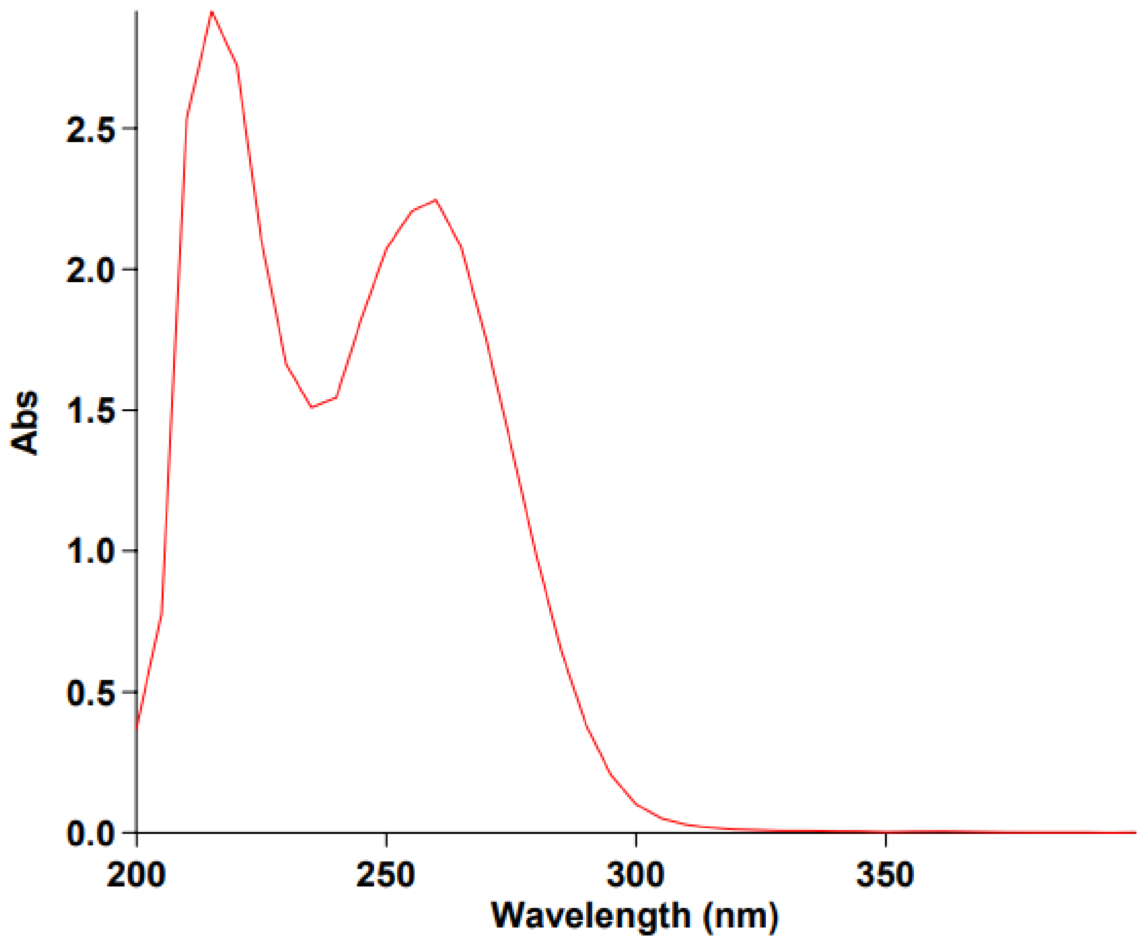

Figure S47. UV spectrum of **3**.

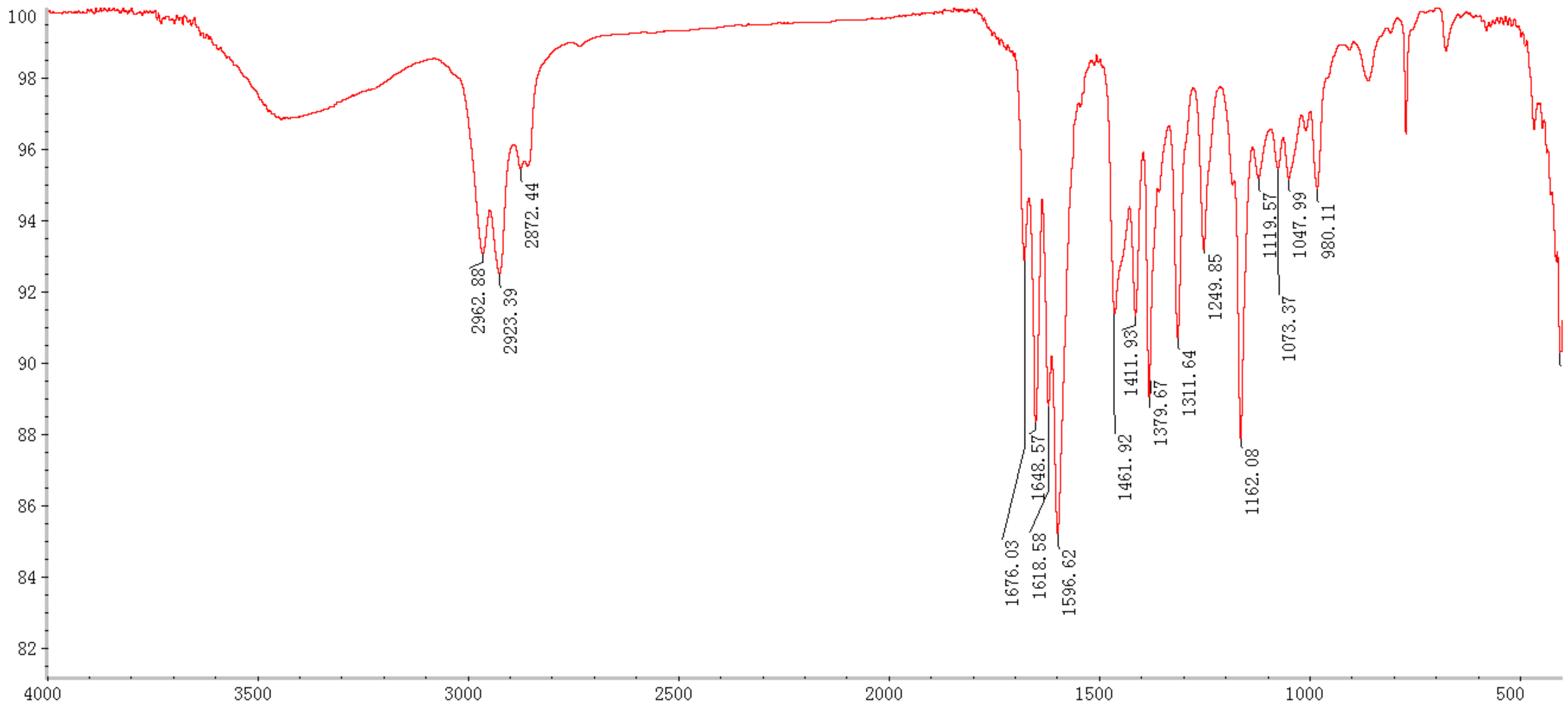

Figure S48. IR spectrum of **3**.

1.8 NMR, HR-ESI-MS, IR, and UV spectra of ocellatuperoxide D (4)

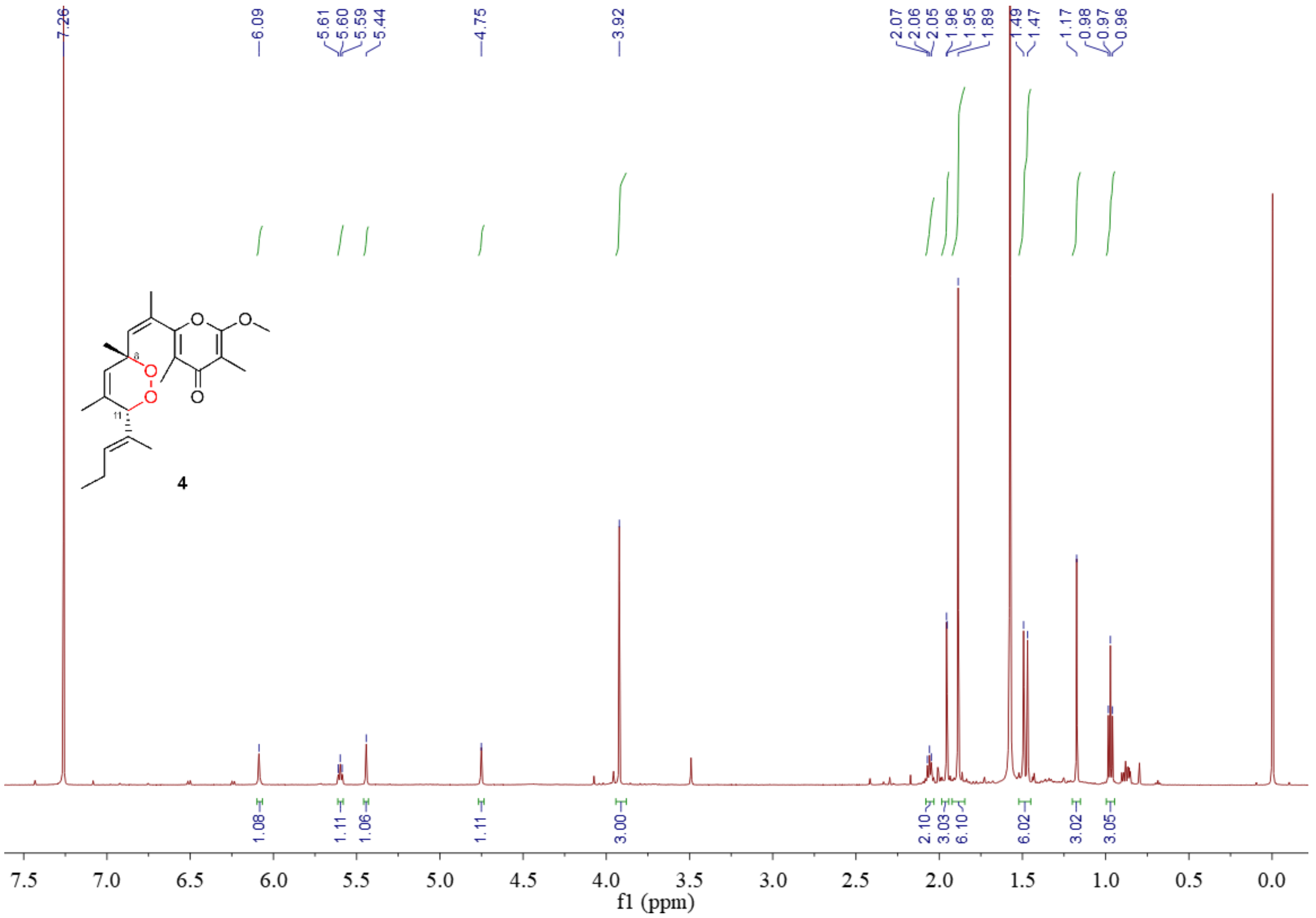

Figure S49. ^{1}H NMR spectrum (600 MHz) of **4** in $CDCl_3$.

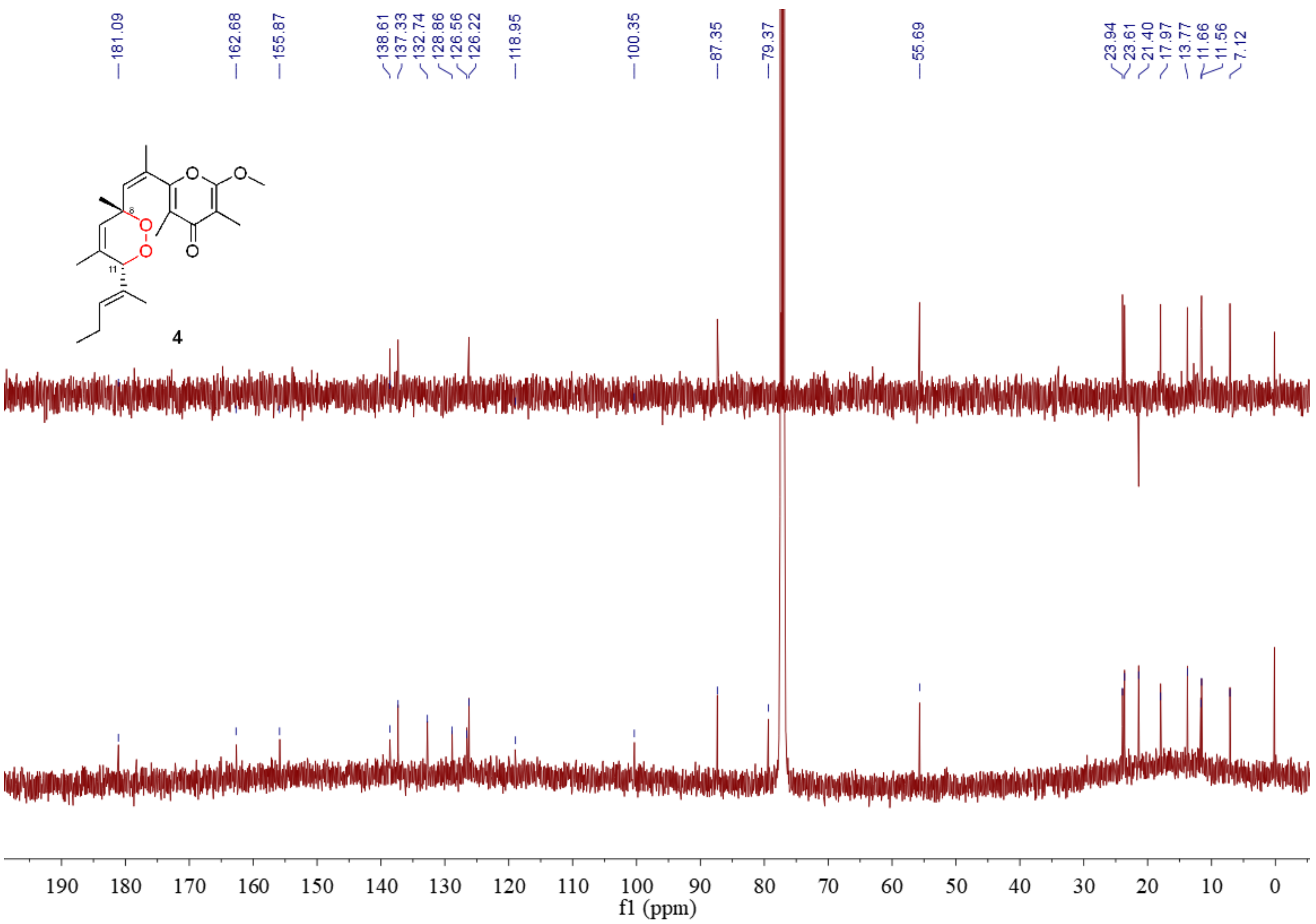

Figure S50. ^{13}C NMR spectrum (150 MHz) of **4** in $CDCl_3$.

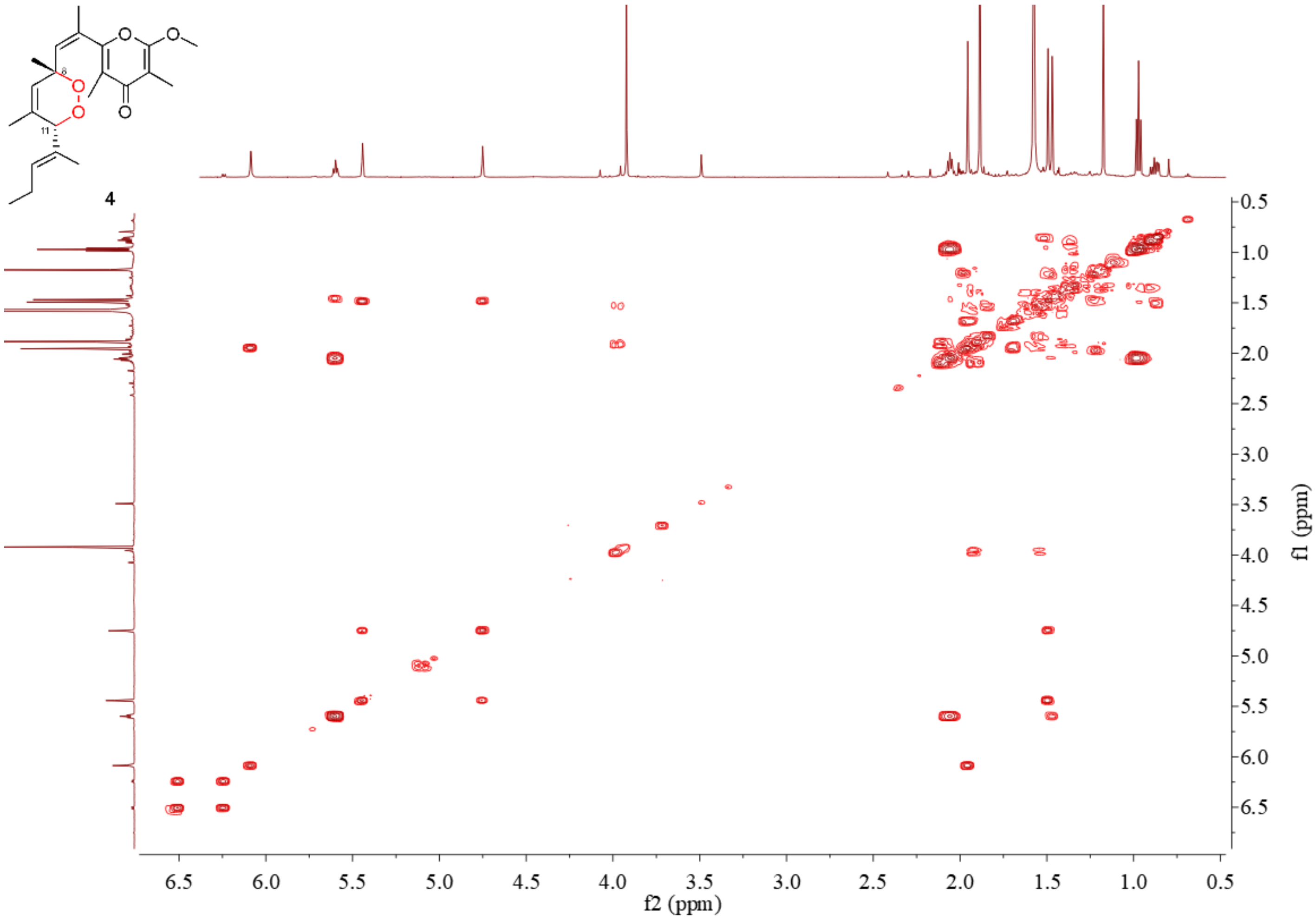

Figure S51. ^{1}H-^{1}H COSY spectrum (600 MHz) of **4** in $CDCl_3$.

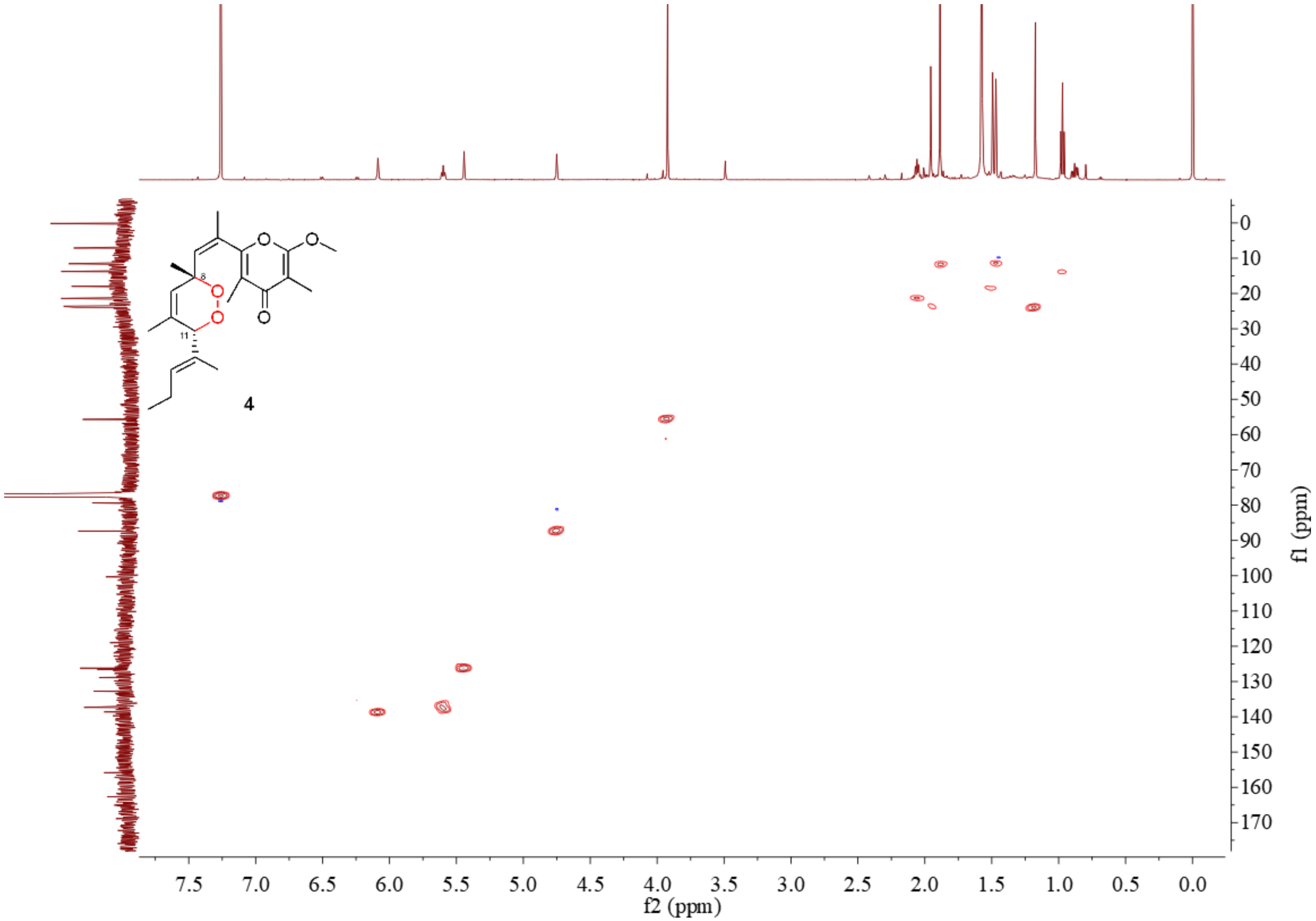

Figure S52. HSQC spectrum (600 MHz) of **4** in $CDCl_3$.

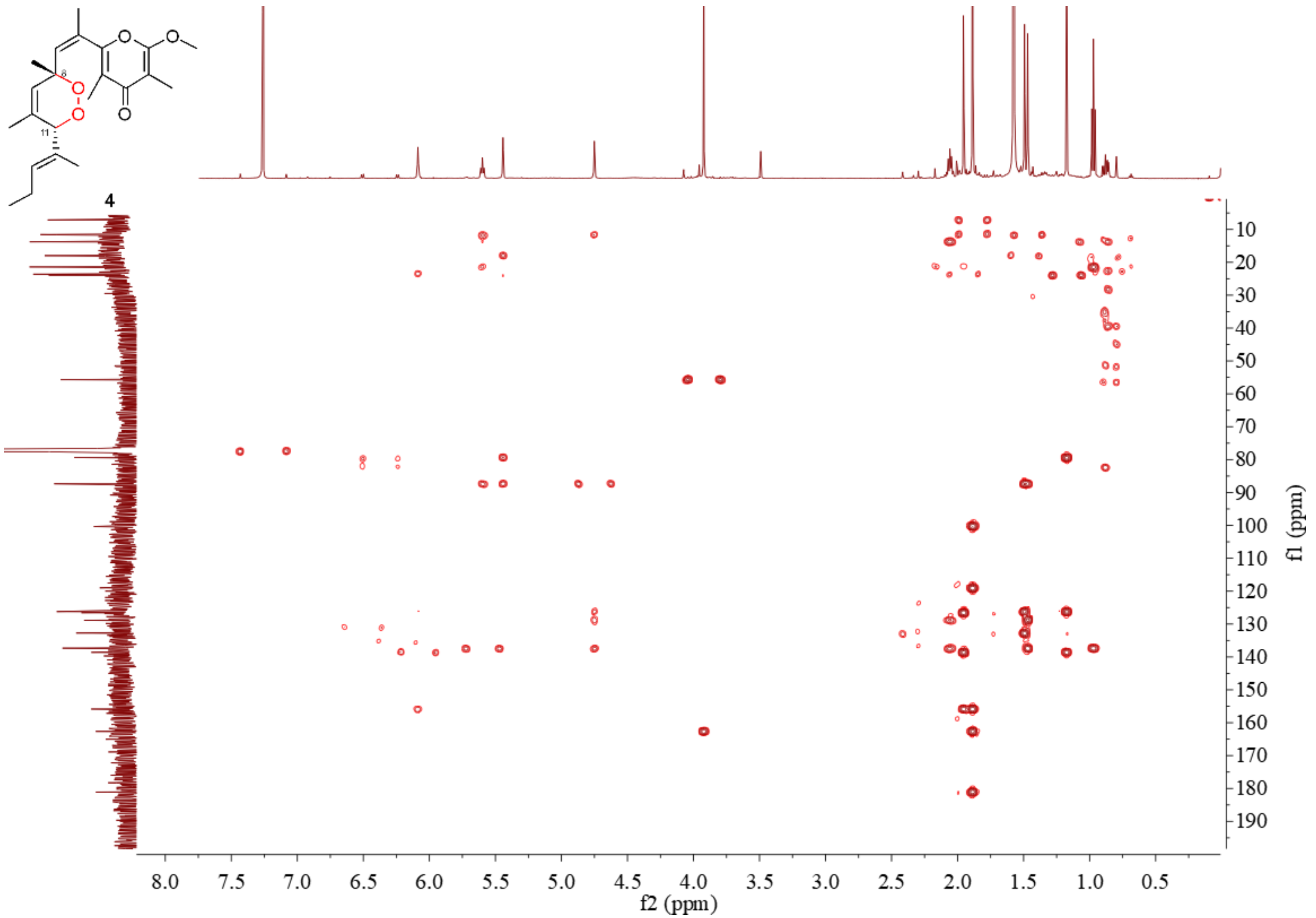

Figure S53. HMBC spectrum (600 MHz) of **4** in $CDCl_3$.

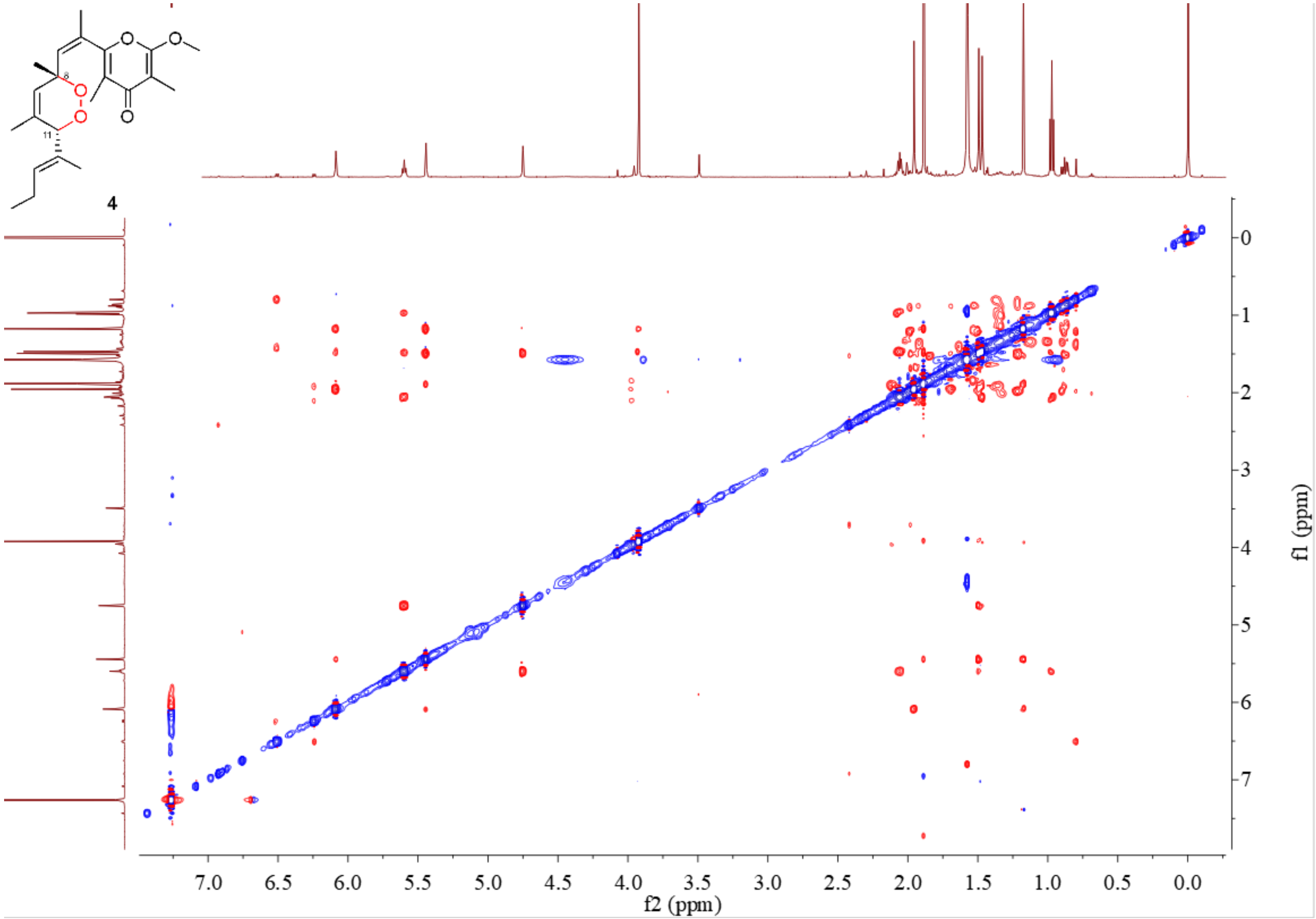

Figure S54. NOESY spectrum (600 MHz) of **4** in $CDCl_3$.

User Spectra

Fragmentor Voltage	Collision Energy	Ionization Mode
175	0	ESI

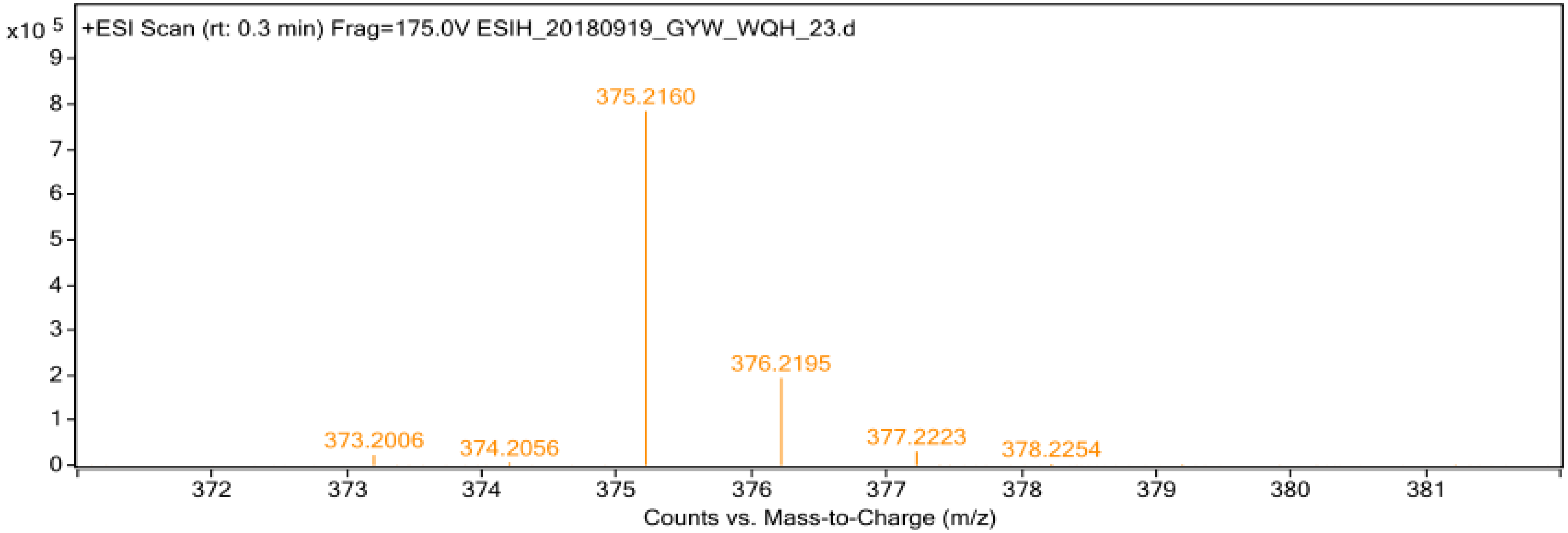

Formula Calculator Results

m/z	Calc m/z	Diff (mDa)	Diff (ppm)	Ion Formula	Ion
375.216	375.2166	0.56	1.5	C22 H31 O5	(M+H)+

Figure S55. HR-ESI-MS (positive mode) spectrum of **4**.

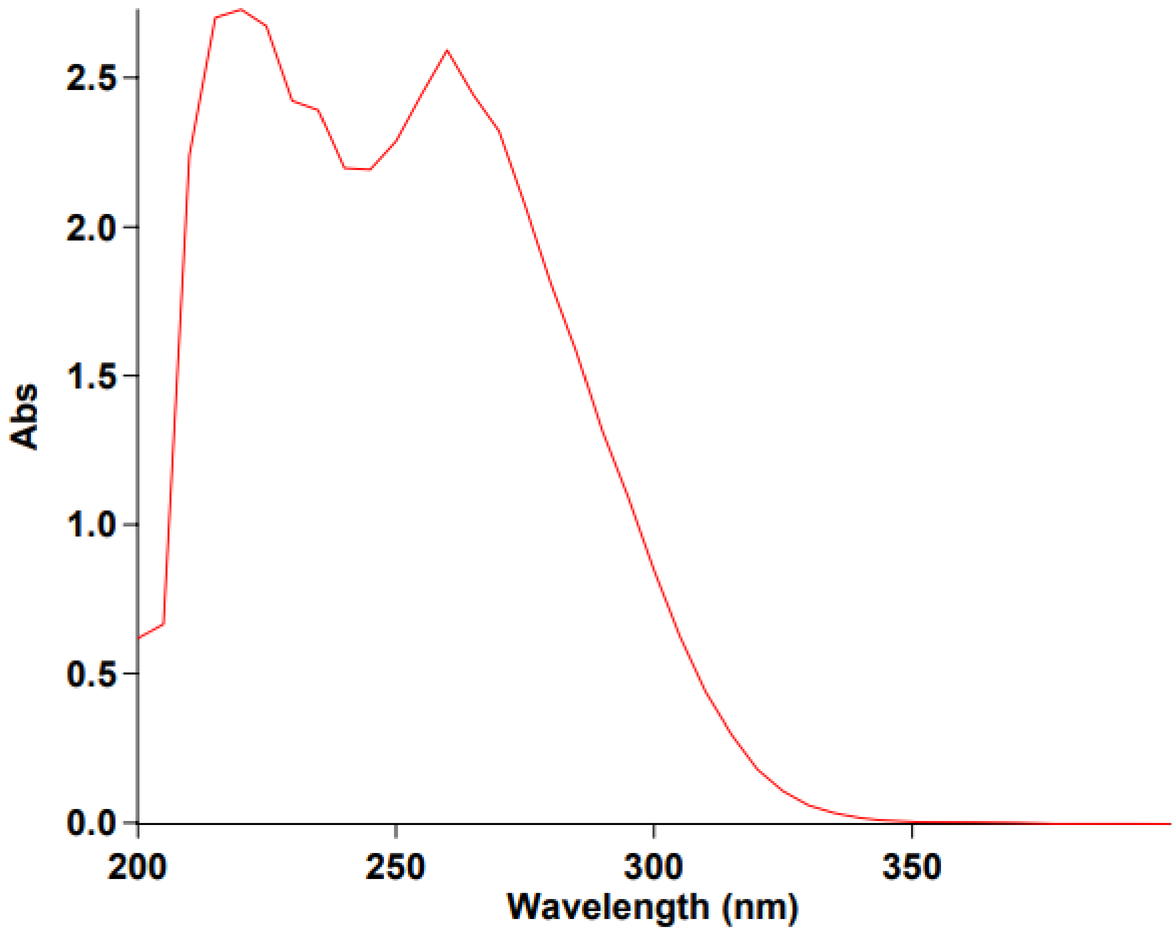

Figure S56. UV spectrum of **4**.

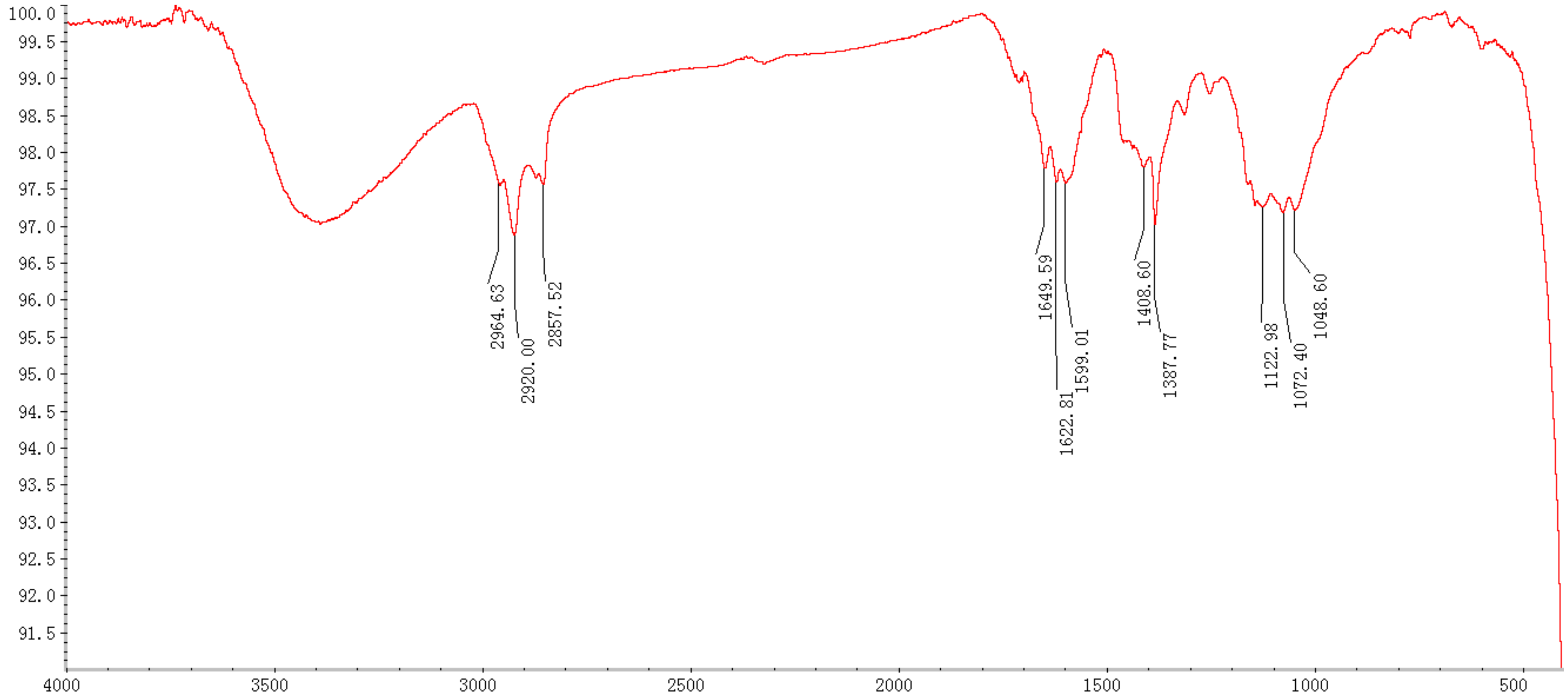

Figure S57. IR spectrum of **4**.

1.9 NMR, HR-ESI-MS, IR, and UV spectra of ocellatuperoxide E (5)

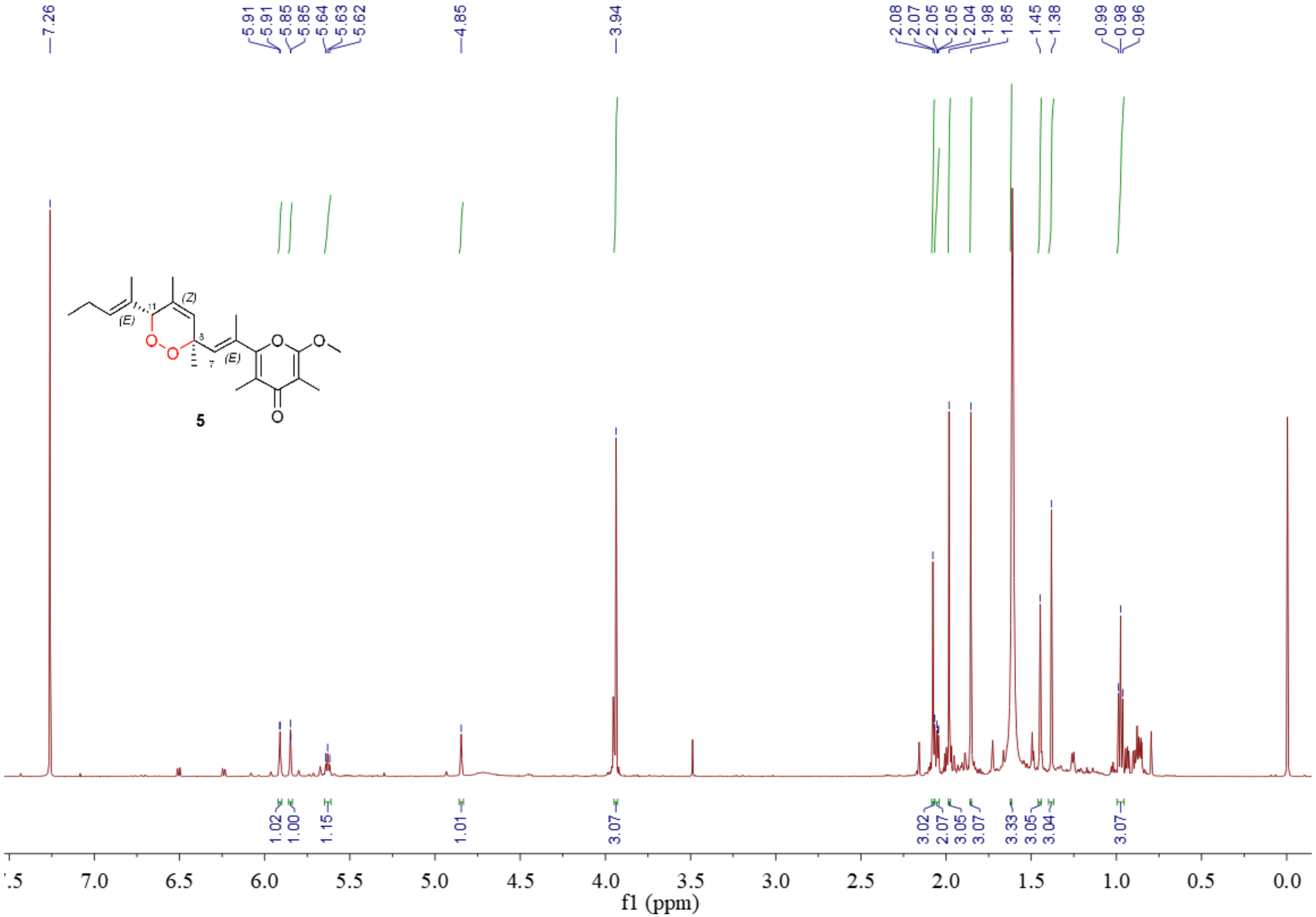

Figure S58. ^{1}H NMR spectrum (600 MHz) of **5** in $CDCl_3$.

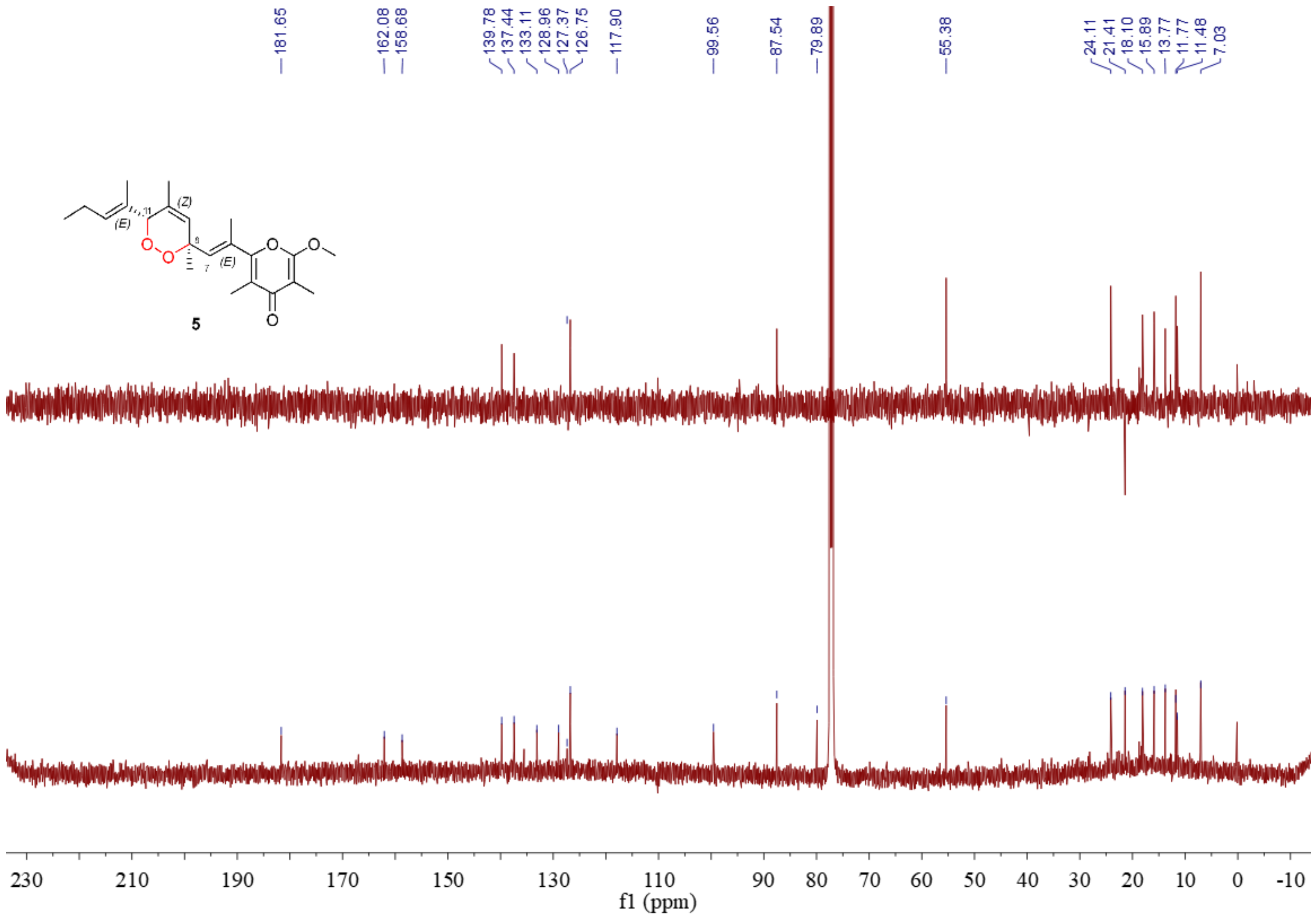

Figure S59. ^{13}C NMR spectrum (150 MHz) of **5** in $CDCl_3$.

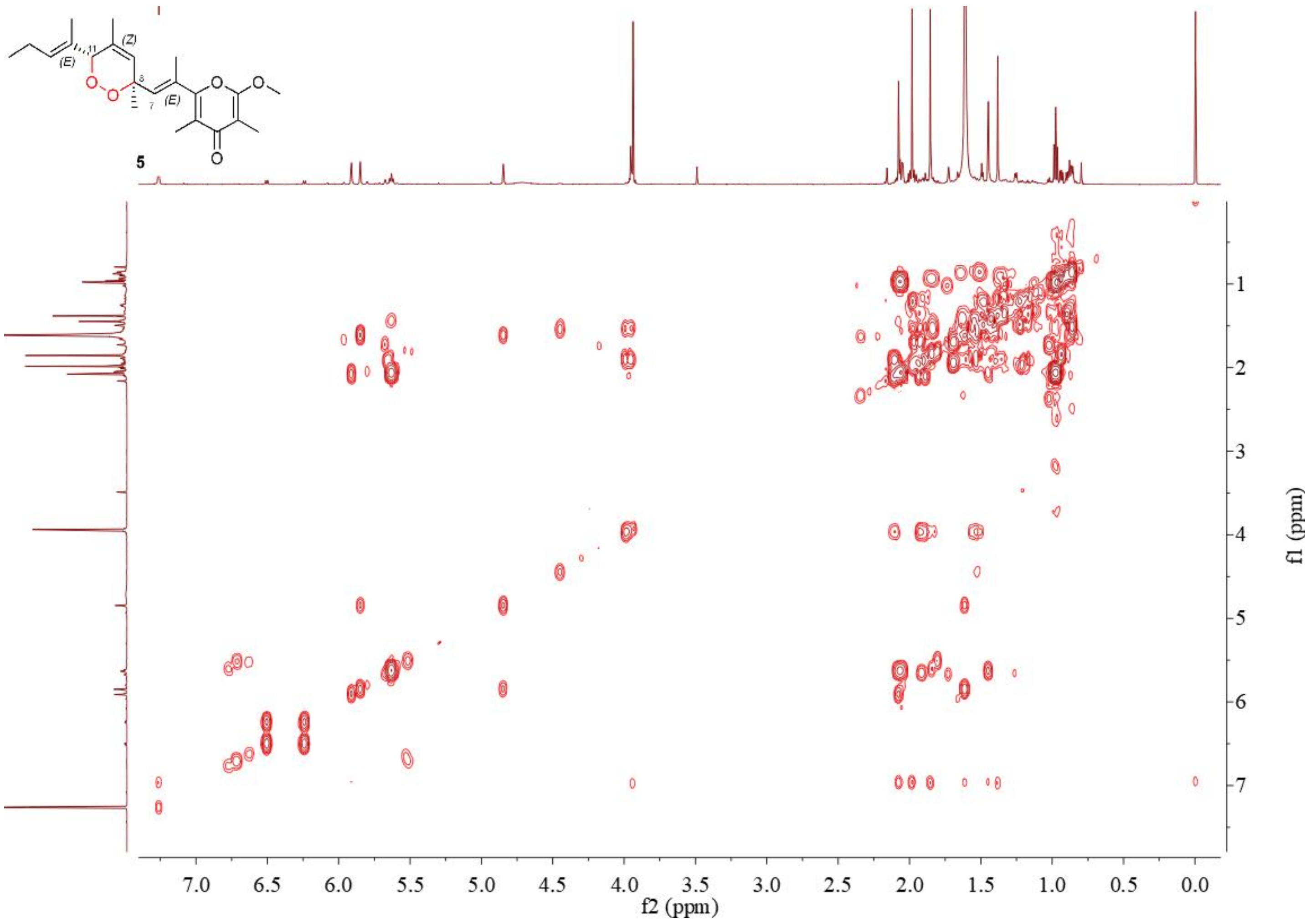

Figure S60. ^{1}H-^{1}H COSY spectrum (600 MHz) of **5** in $CDCl_3$.

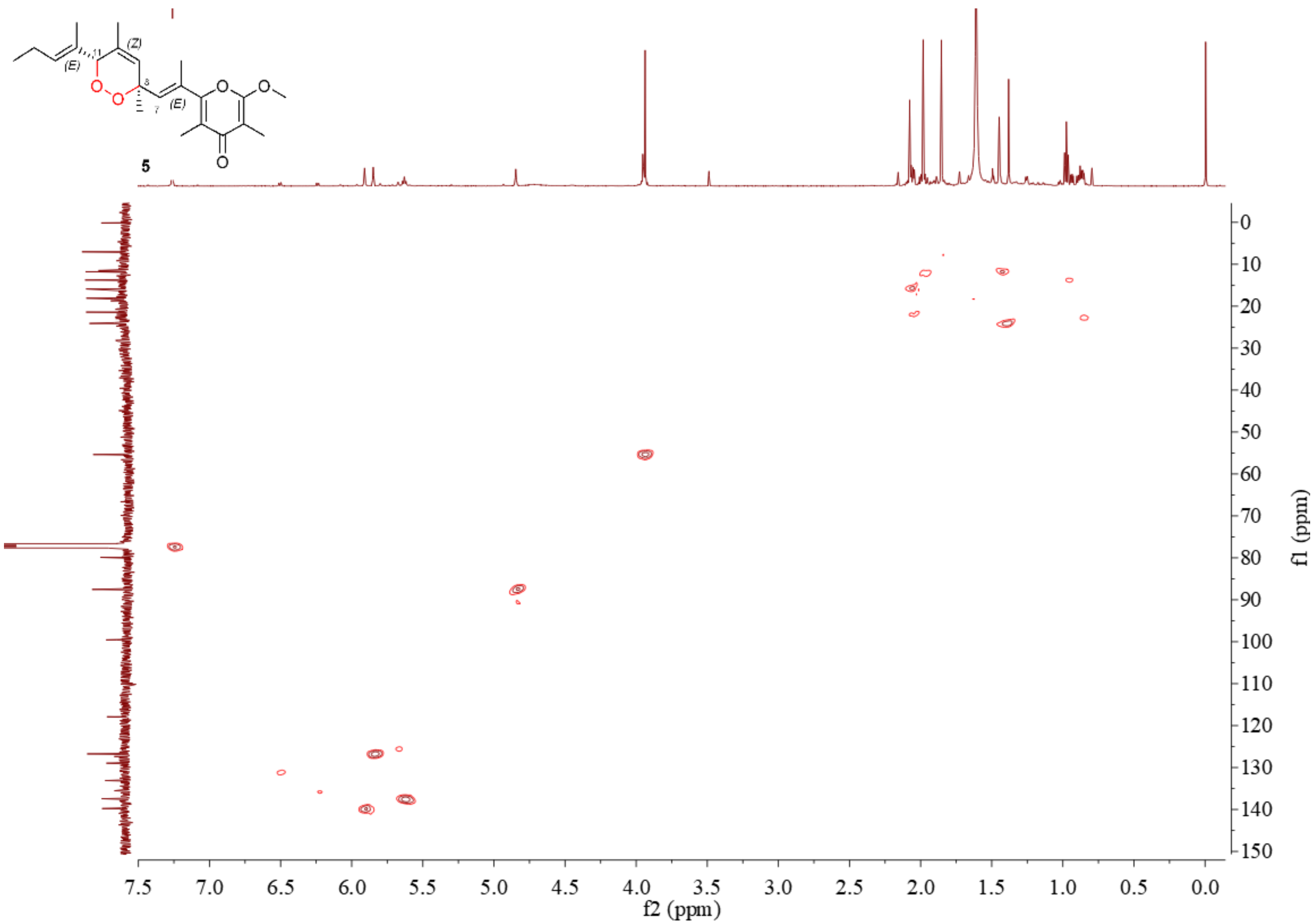

Figure S61. HSQC spectrum (600 MHz) of **5** in $CDCl_3$.

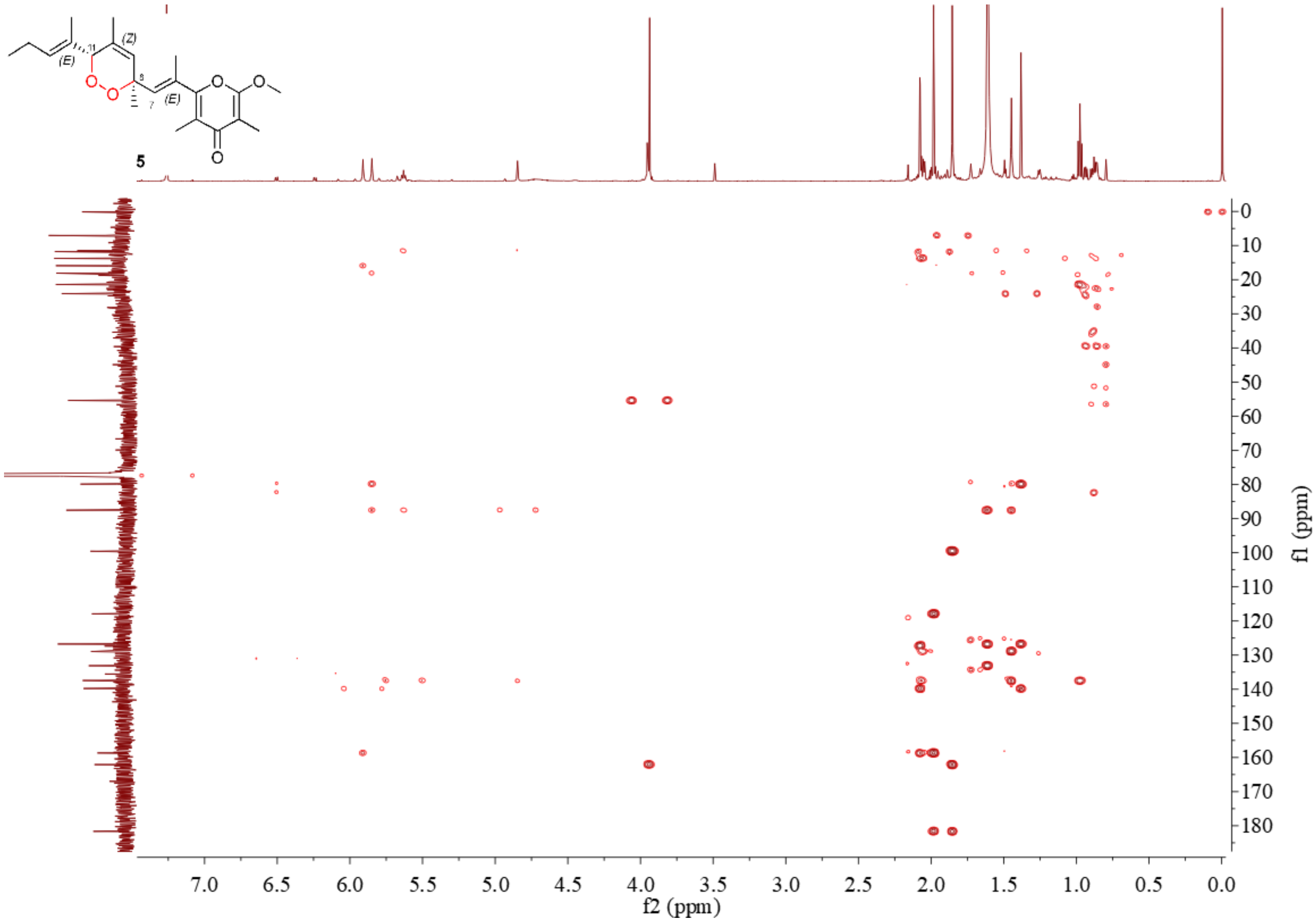

Figure S62. HMBC spectrum (600 MHz) of **5** in $CDCl_3$.

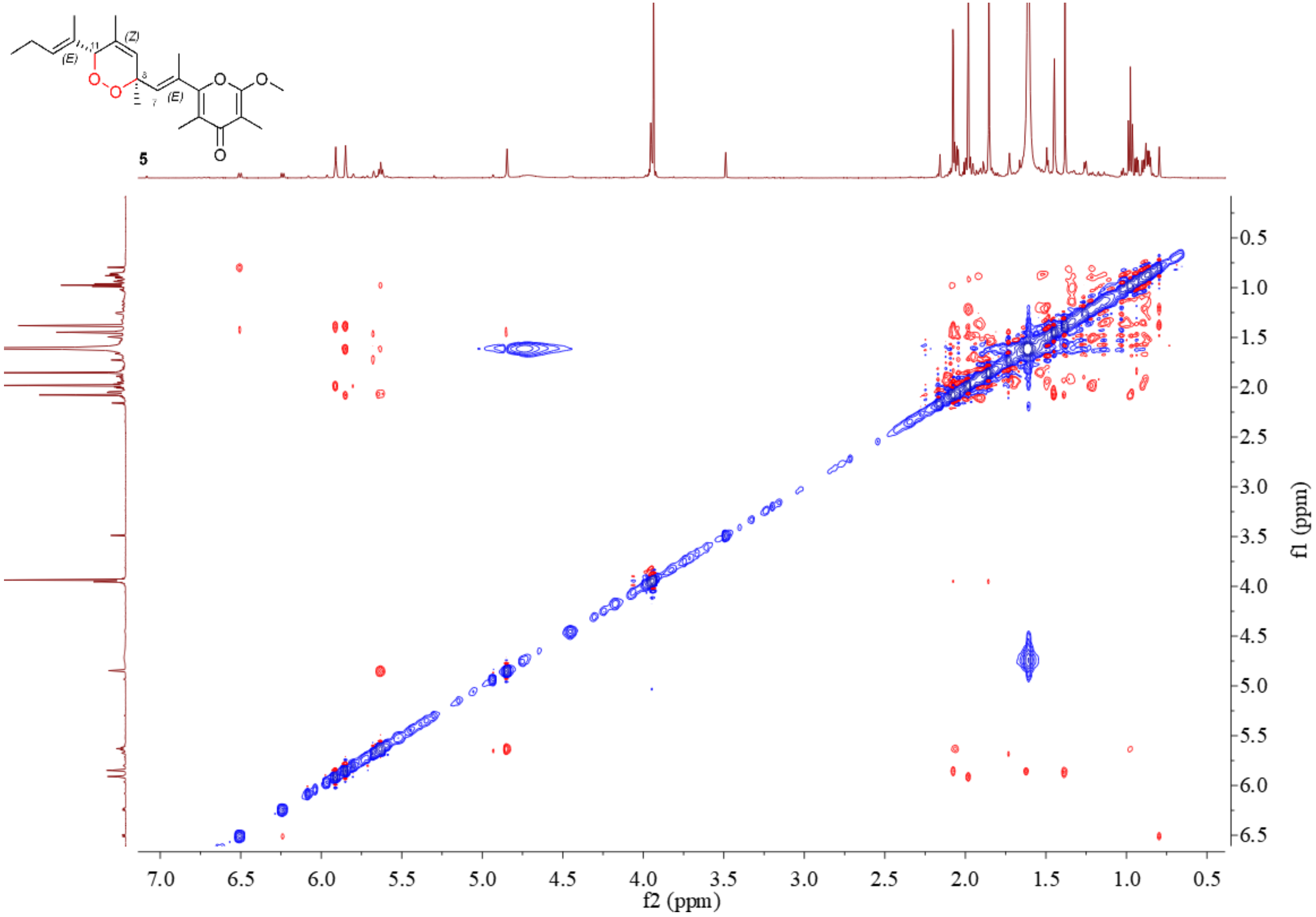

Figure S63. NOESY spectrum (600 MHz) of **5** in $CDCl_3$.

User Spectra

Fragmentor Voltage	Collision Energy	Ionization Mode
175	0	ESI

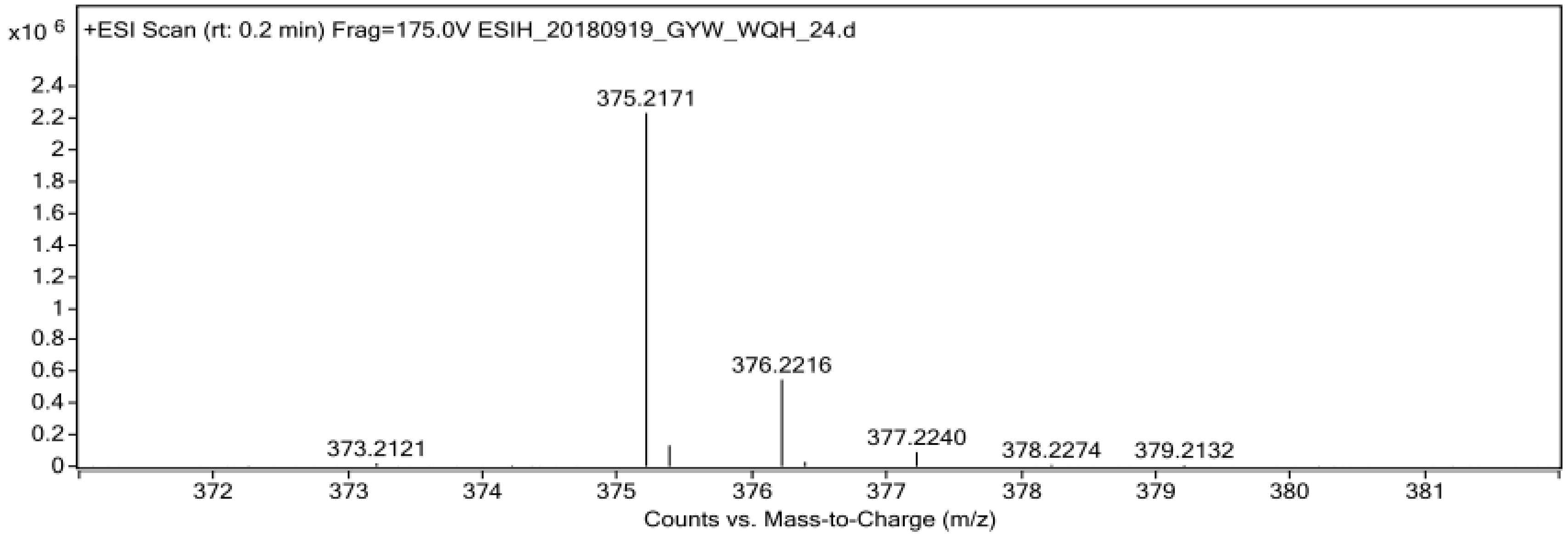

Formula Calculator Results

m/z	Calc m/z	Diff (mDa)	Diff (ppm)	Ion Formula	Ion
375.2171	375.2166	-0.46	-1.23	C22 H31 O5	(M+H)+

Figure S64. HR-ESI-MS (positive mode) spectrum of **5**.

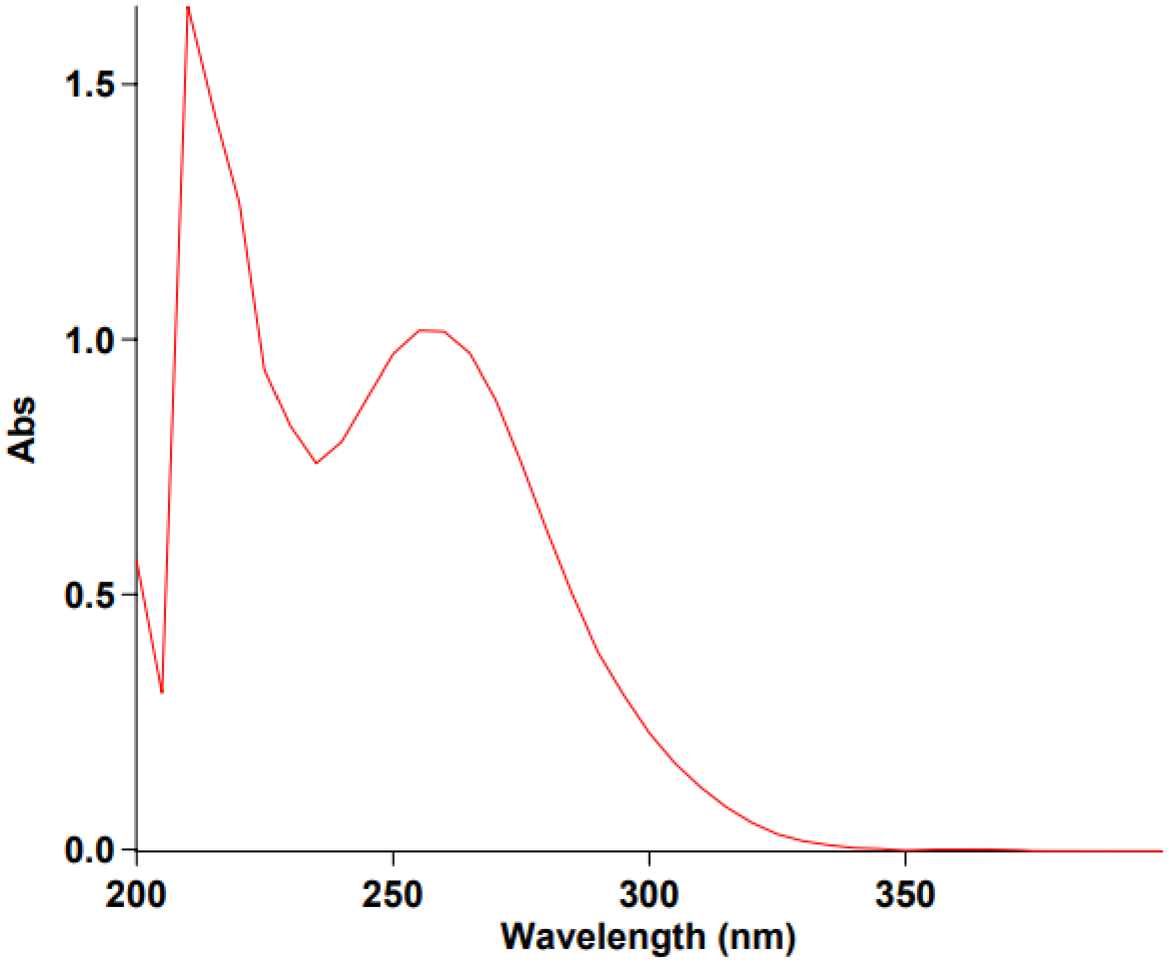

Figure S65. UV spectrum of **5**.

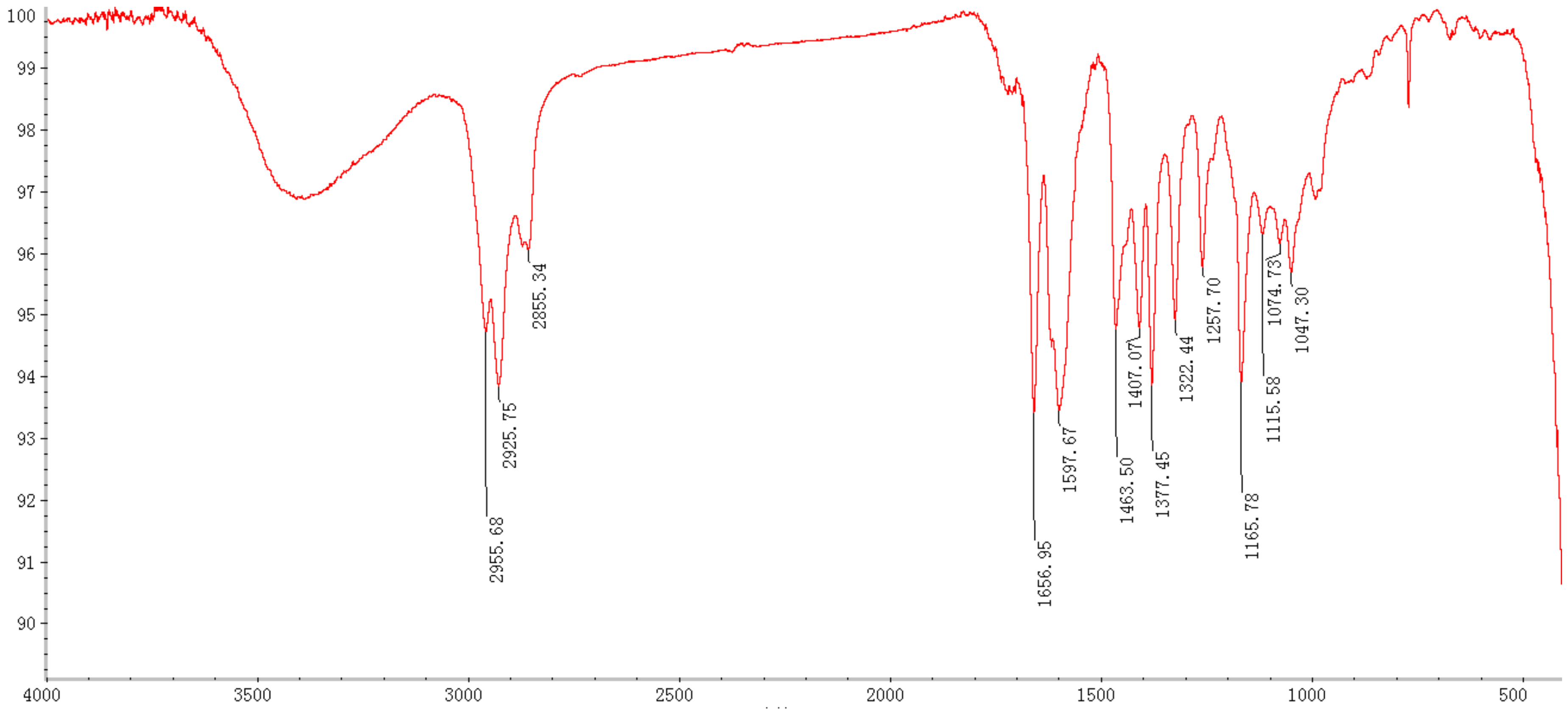

Figure S66. IR spectrum of **5**.

1.10 NMR, HR-ESI-MS, IR, and UV spectra of ocellatuperoxide F (6)

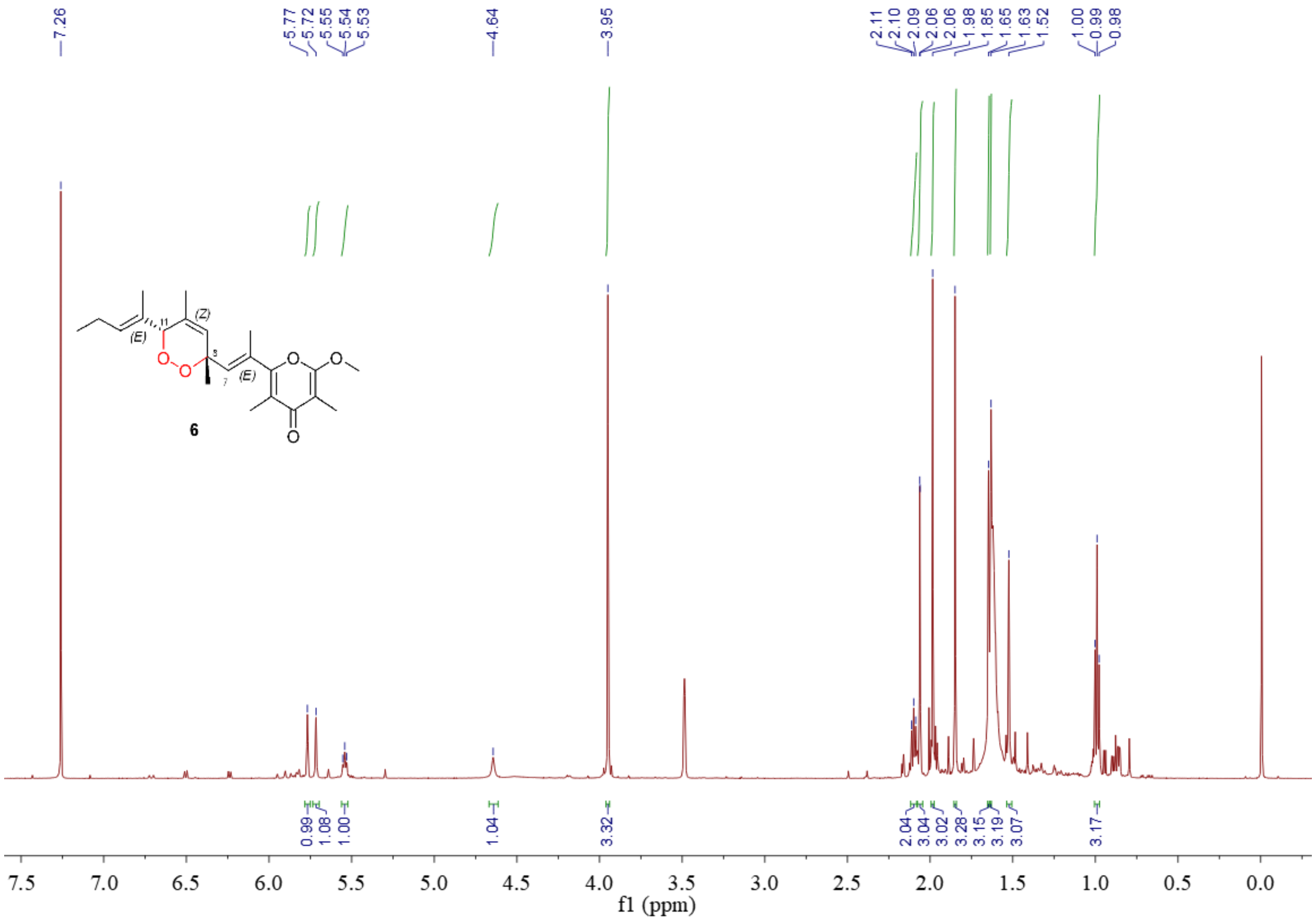

Figure S67. ^{1}H NMR spectrum (600 MHz) of **6** in $CDCl_3$.

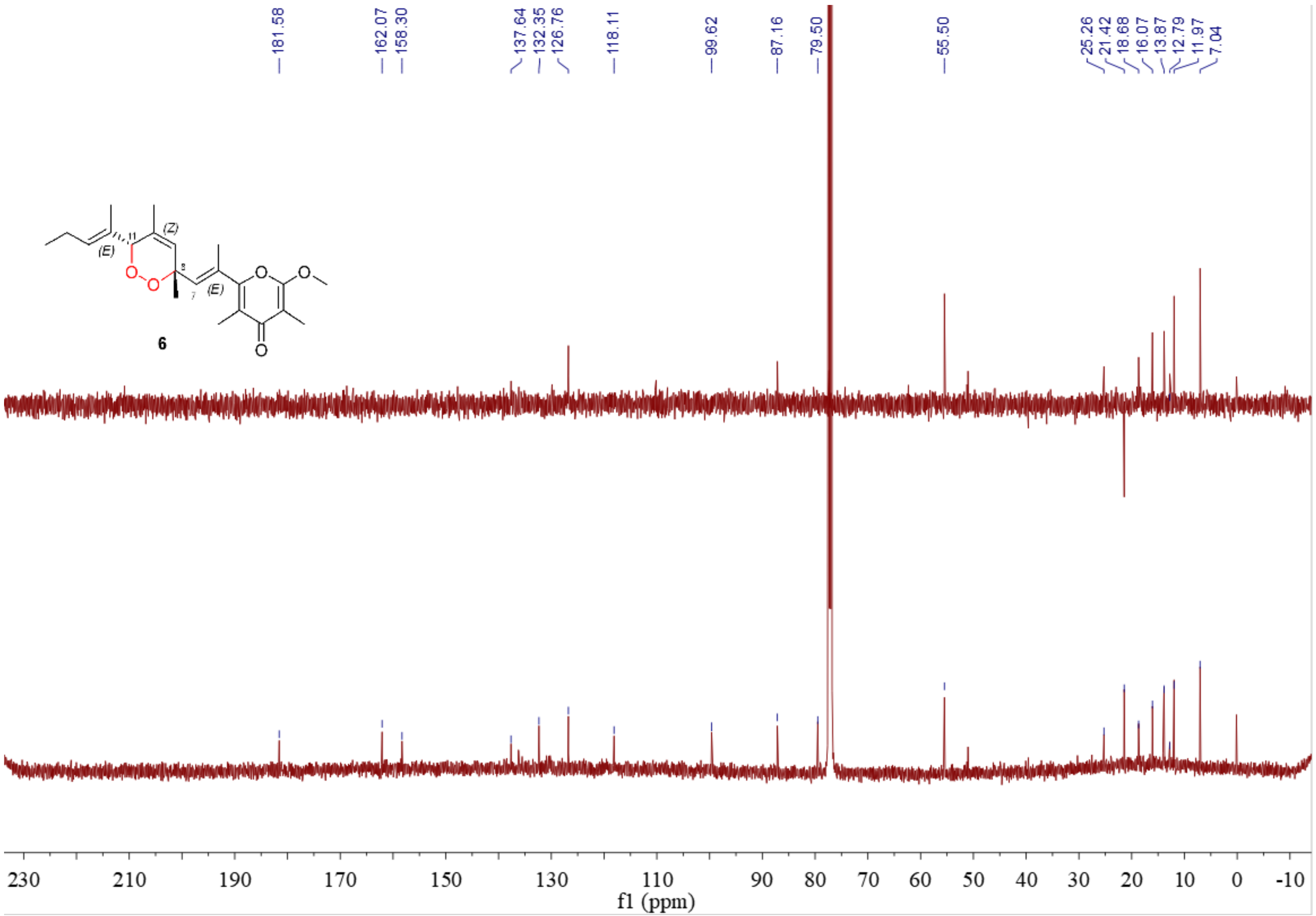

Figure S68. ^{13}C NMR spectrum (150 MHz) of **6** in $CDCl_3$.

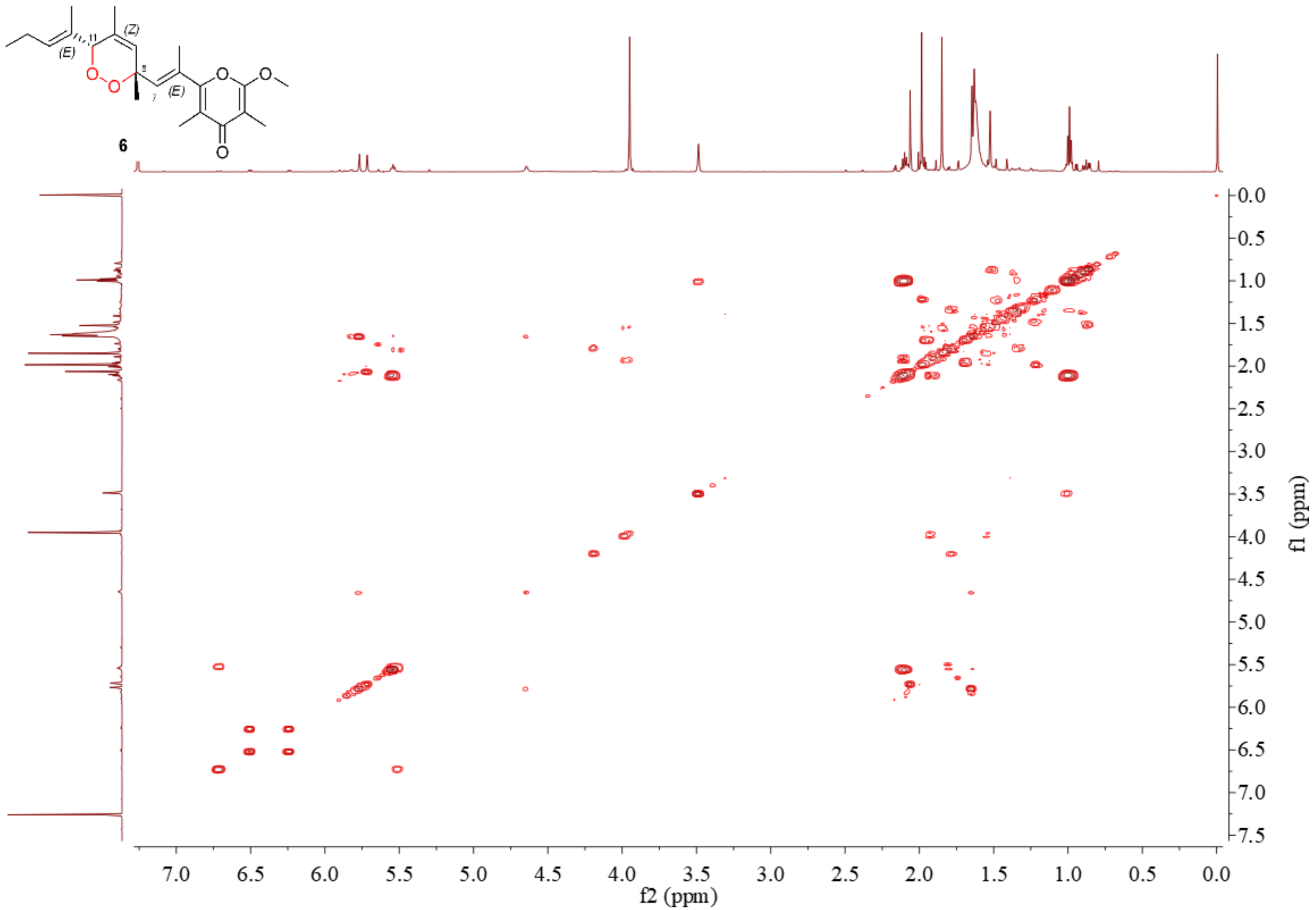

Figure S69. ^{1}H-^{1}H COSY spectrum (600 MHz) of **6** in $CDCl_3$.

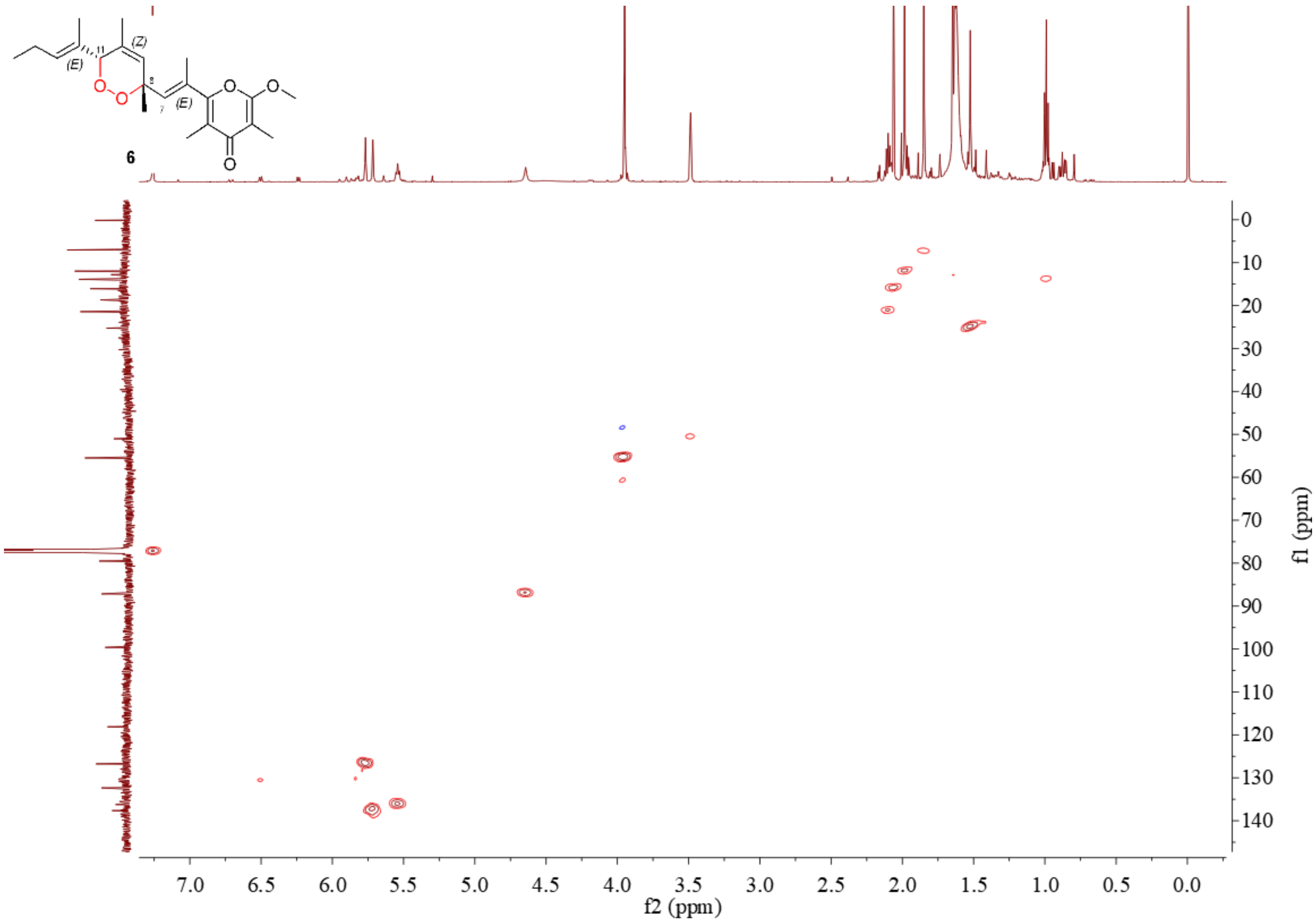

Figure S70. HSQC spectrum (600 MHz) of **6** in $CDCl_3$.

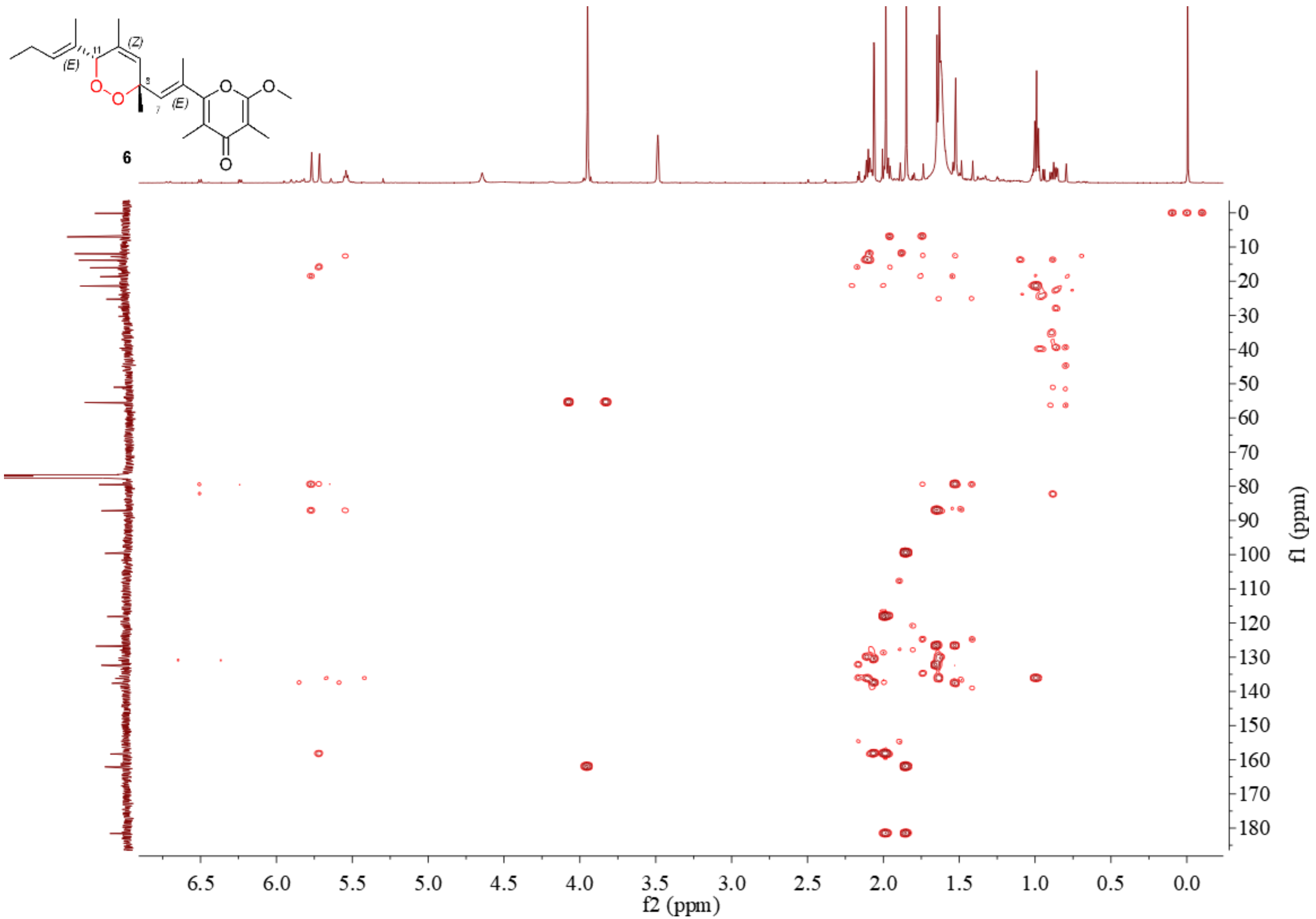

Figure S71. HMBC spectrum (600 MHz) of **6** in $CDCl_3$.

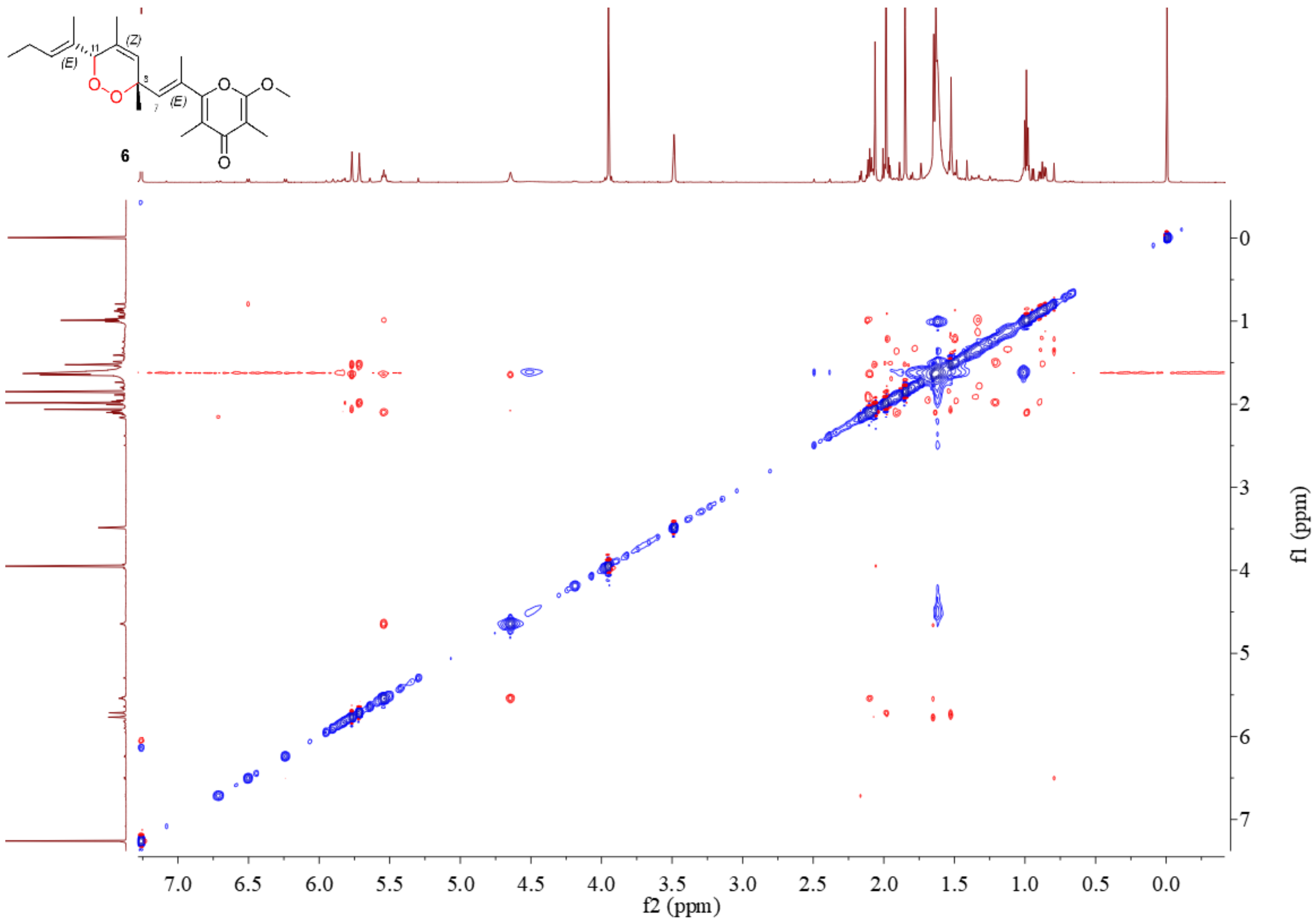

Figure S72. NOESY spectrum (600 MHz) of **6** in $CDCl_3$.

User Spectra

Fragmentor Voltage	Collision Energy	Ionization Mode
175	0	ESI

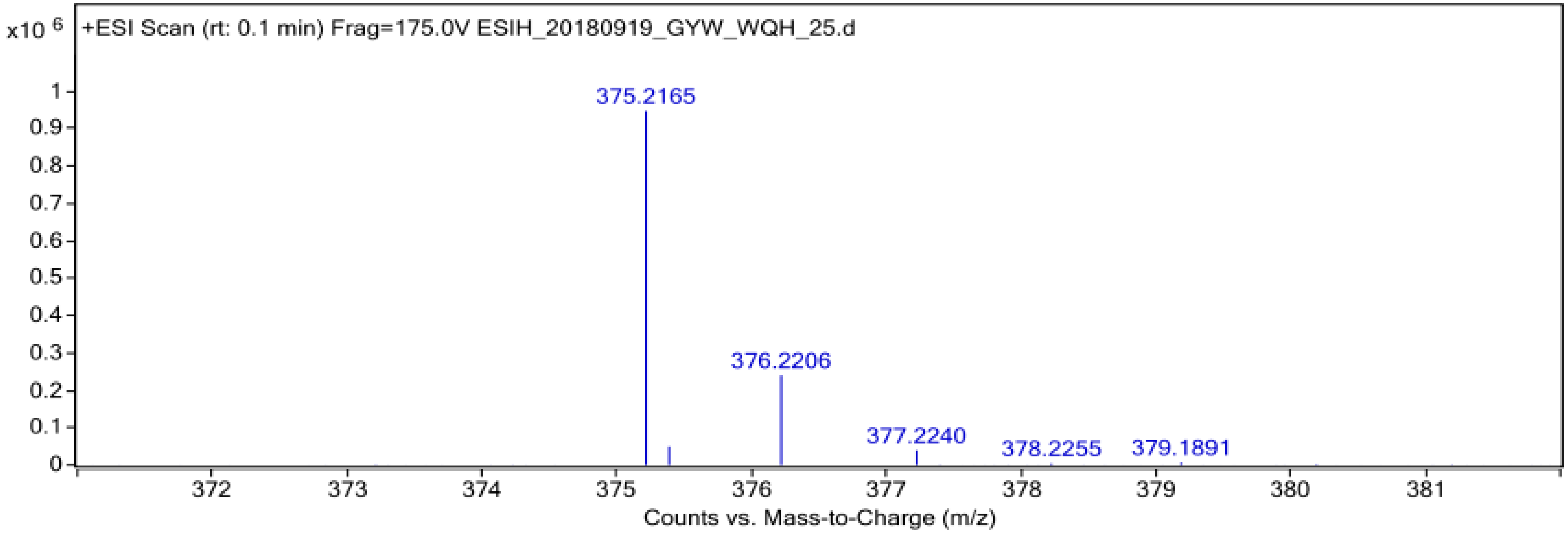

Formula Calculator Results

m/z	Calc m/z	Diff (mDa)	Diff (ppm)	Ion Formula	Ion
375.2165	375.2166	0.08	0.23	C22 H31 O5	(M+H)+

Figure S73. HR-ESI-MS (positive mode) spectrum of **6**.

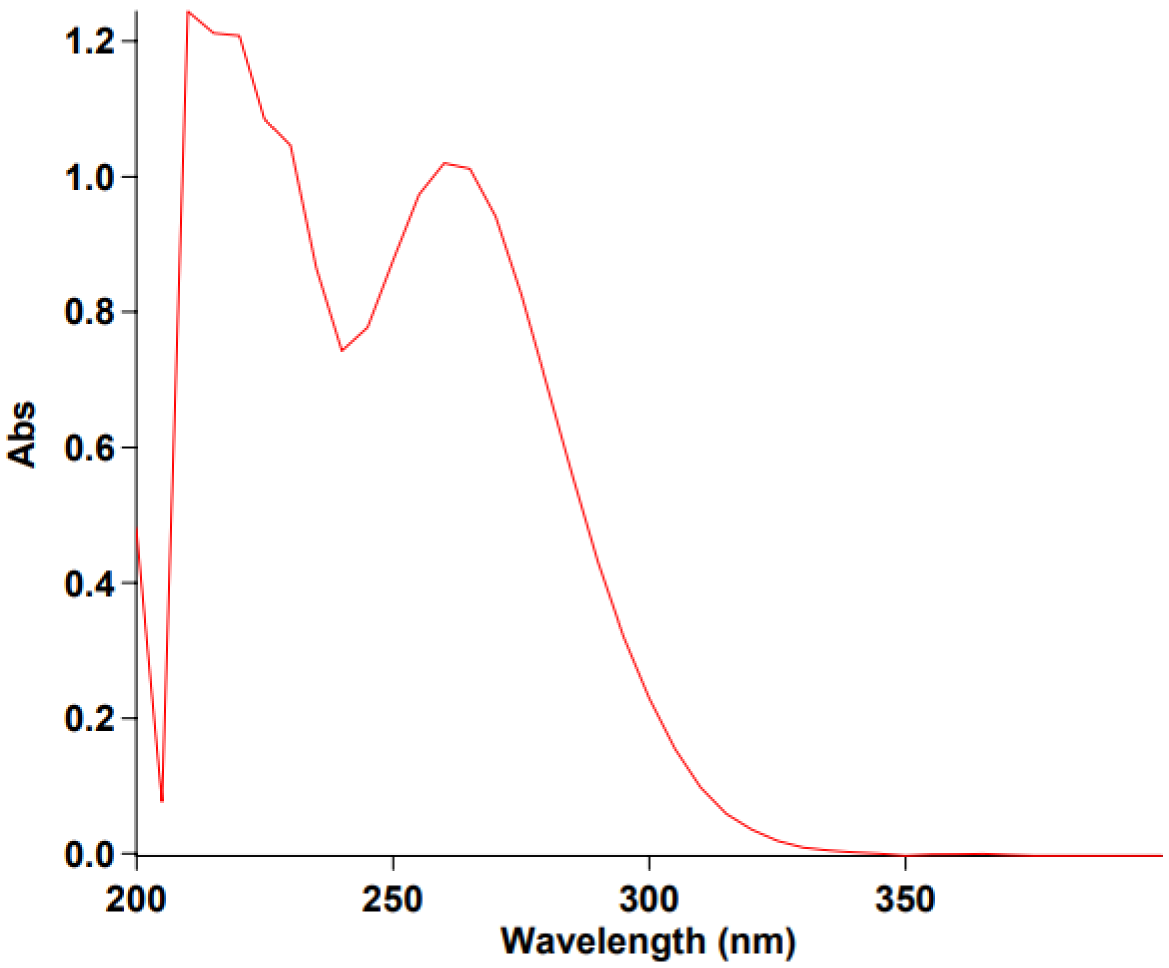

Figure S74. UV spectrum of **6**.

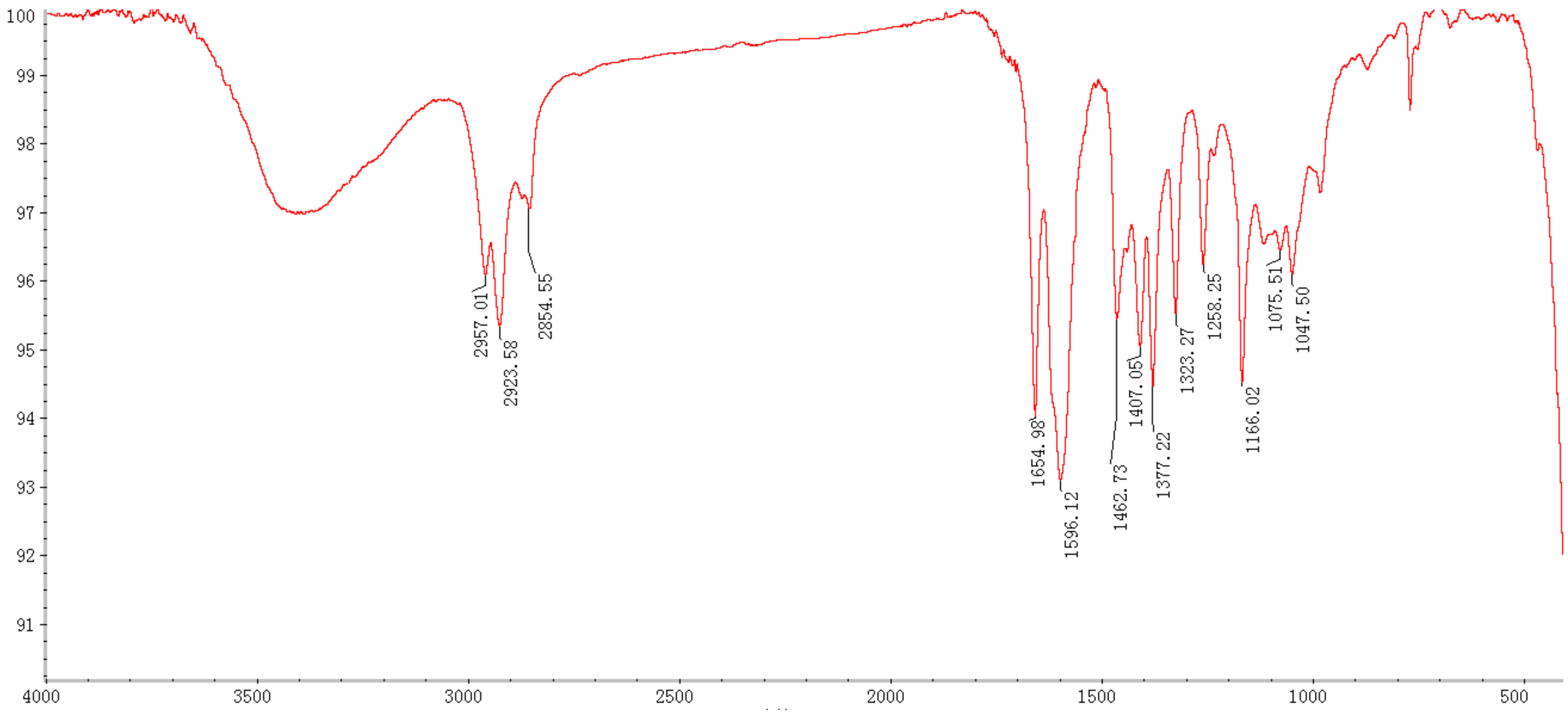

Figure S75. IR spectrum of **6**.

2. Computational Section

2.1 Computational details

Conformational searches were carried out using the torsional sampling (MCMM) method and OPLS_2005 force field in the Macromodel 9.9.223 software applying an energy window of 21 kJ/mol, which afforded 5 conformers for (8*R*, 11*R*)-**1**, 3 conformers for (8*R*, 11*S*)-**3**, and 3 conformers for (8*R*, 11*S*)-**5** above 4% population for re-optimization to keep the computational cost to a minimum. These conformers were re-optimized with Gaussian 09 using DFT at the B3LYP/6-311G(d,p) level of theory, all of which were subjected to TDDFT-ECD calculations at the mPW1PW91/6-31G** level of theory.

2.3 Cartesian Coordinates, Relative Energies, and Boltzmann populations of all the calculated Low-energy conformers

Table S2. Cartesian Coordinates, Relative Energies, and Boltzmann Populations of Low-energy conformers of (8*R*,11*R*)-**1.**

Conformer **1-1**:

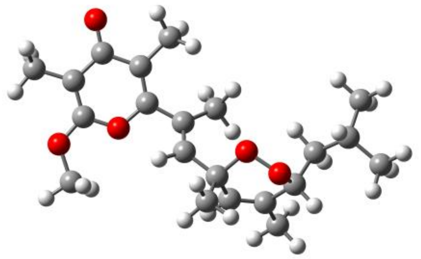

C	4.21027300	-0.65526600	0.18327400
C	4.94990400	0.48547500	0.12911600
C	4.25140200	1.74650400	-0.00040700
C	2.77605500	1.69175200	-0.07687000
C	2.14341500	0.49785500	0.01691700
O	2.86935800	-0.67181100	0.13523000
C	6.44982900	0.47812500	0.19950400
C	2.06856200	3.00140900	-0.29275600
O	4.85606300	2.82900100	-0.05777500
O	4.76218300	-1.86138500	0.30604300
C	3.91998100	-3.03449900	0.33149700
C	0.68425500	0.21989300	0.00670700
C	0.25627500	-0.85043400	-0.68349400
C	-0.20837600	1.13198000	0.81653200
C	-1.16432100	-1.37026900	-0.84860600
O	-1.94280300	-0.22131100	-1.27154100
O	-3.34309900	-0.63696400	-1.33409300
C	-3.86078800	-0.71788200	0.00644200
C	-2.96927400	-1.58815500	0.86960100
C	-1.74230800	-1.90025900	0.44862400
C	-1.18450300	-2.44921500	-1.94244500
C	-4.16168800	0.67099500	0.58805300
C	-3.55071200	-2.10556800	2.15757600
C	-5.21272500	1.48170800	-0.19451500
C	-6.62704100	0.90237100	-0.04623700
C	-5.18537200	2.95137700	0.24776600
H	6.84543500	-0.53634700	0.20765900
H	6.87280800	1.01505800	-0.65401600
H	6.79848600	0.99367300	1.09980600
H	2.71821600	3.65180000	-0.87954100
H	1.12052800	2.88250000	-0.81438900
H	1.87794900	3.51758000	0.65369200
H	3.21733400	-2.98831800	1.16355400
H	3.38000800	-3.14070100	-0.60980500
H	4.60710300	-3.86569900	0.46475400
H	0.99802000	-1.43763600	-1.21334100
H	-0.66017400	1.91304500	0.20206200
H	-1.02063100	0.55645000	1.25835800
H	0.35241700	1.61493700	1.61778800
H	-4.79876100	-1.26143400	-0.16123400
H	-1.11201100	-2.57328800	1.02198300
H	-0.61013600	-3.32251900	-1.62320600
H	-2.20986400	-2.75893000	-2.13958600
H	-0.74218600	-2.05796400	-2.86241700
H	-4.49536200	0.55661700	1.62565900
H	-3.22092300	1.22648300	0.62001200
H	-2.85739800	-2.78717400	2.65320400
H	-3.77788500	-1.29120900	2.85294600
H	-4.49011100	-2.63956900	1.97733200
H	-4.93520900	1.44289300	-1.25412500
H	-7.34759700	1.48664400	-0.62567600
H	-6.69246700	-0.13192400	-0.39447200
H	-6.94617100	0.92330600	1.00174400
H	-5.91056400	3.54510200	-0.31633700
H	-5.43457400	3.04425500	1.31079200

H	-4.19695200	3.39475400	0.09622300

Relative Energy = -1194.61412550 a.u.
Number of Imaginary Frequencies = 0
P (%) = 33.75%

Conformer **1-2**:

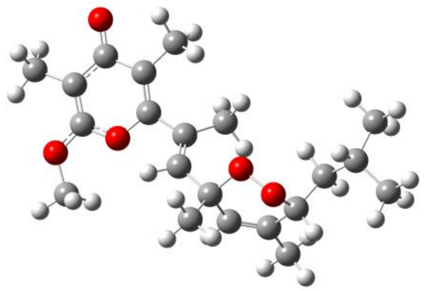

C	-4.20109900	-0.66340300	-0.21034000
C	-4.94596400	0.47377800	-0.15343500
C	-4.25603500	1.73476800	0.01792400
C	-2.78165100	1.68516600	0.10591100
C	-2.14293500	0.49618000	-0.00041200
O	-2.86054500	-0.67555600	-0.14373100
C	-6.44447700	0.46173100	-0.24764600
C	-2.07912100	2.99266700	0.34748200
O	-4.86682800	2.81251600	0.09536400
O	-4.74681300	-1.87065700	-0.34858000
C	-3.89515400	-3.03203000	-0.45929600
C	-0.68179500	0.22753500	0.02064700
C	-0.25122000	-0.82622000	0.73346300
C	0.20665600	1.13047100	-0.80439100
C	1.17122900	-1.33566800	0.91462200
O	1.95191900	-0.16977300	1.28068700
O	3.35298200	-0.58108100	1.34785900
C	3.85909200	-0.71874800	0.00774500
C	2.96077200	-1.62556800	-0.80947400
C	1.73850500	-1.92127700	-0.36356100
C	1.19835000	-2.36527300	2.05498900
C	4.15530300	0.64313400	-0.63678200
C	3.53059300	-2.19596400	-2.08016200
C	5.20006300	1.49473800	0.10992300
C	6.61720200	0.91479700	-0.00403500
C	5.16884200	2.94131400	-0.40295200
H	-6.82272900	-0.52781500	-0.50016400
H	-6.89698900	0.77322400	0.69931000
H	-6.78070000	1.17348800	-1.00608300
H	-2.72088200	3.62218100	0.96519600
H	-1.12052100	2.86516500	0.84729900
H	-1.91045300	3.53698700	-0.58734700
H	-3.22818100	-2.94188900	-1.31695900
H	-3.31527900	-3.17243900	0.45306800
H	-4.57916100	-3.86451600	-0.60036000
H	-0.99249600	-1.40825100	1.27014500
H	-0.36002900	1.60804500	-1.60475200
H	0.66601300	1.91514000	-0.20026700
H	1.01356500	0.54869600	-1.24836200
H	4.79885700	-1.25429700	0.19057800
H	1.10394300	-2.61910800	-0.90139000
H	0.62002600	-3.25071200	1.77934900
H	2.22448500	-2.66830500	2.25834700
H	0.76364200	-1.93258400	2.95992200
H	4.49336100	0.48076700	-1.66655800
H	3.21285200	1.19317800	-0.69901400
H	2.83514400	-2.90130400	-2.53816800
H	3.74642500	-1.41162300	-2.81265300
H	4.47435300	-2.71776500	-1.88740200
H	4.91792300	1.50621100	1.16893000
H	7.33285200	1.53016100	0.54869300
H	6.68547300	-0.10070400	0.39525200
H	6.94053700	0.88561800	-1.05056500
H	5.88969300	3.56430200	0.13453400
H	5.42142000	2.98420900	-1.46835300
H	4.17820300	3.38757800	-0.27632400

Relative Energy = -1194.61411067 a.u.
Number of Imaginary Frequencies = 0
P (%) = 30.74%

Conformer **1-3**:

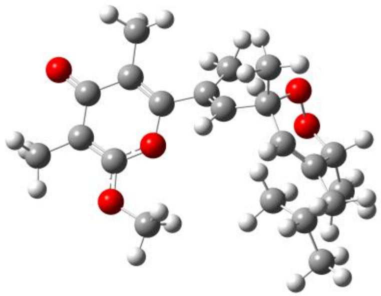

C	2.91184300	1.39758400	0.56258700
C	4.18341400	1.04024200	0.23644600
C	4.39706000	-0.26756200	-0.34815000
C	3.20586400	-1.11568900	-0.54064200
C	1.98053000	-0.64064800	-0.21474600
O	1.84235000	0.61472000	0.34567200
C	5.36174400	1.94045300	0.47284100
C	3.44654100	-2.50506600	-1.06278400
O	5.52921300	-0.67074100	-0.66094500
O	2.61784300	2.57204700	1.11994900
C	1.24548000	2.88693300	1.44103000
C	0.66600200	-1.31782100	-0.36140600
C	-0.16006100	-1.29020900	0.69636900
C	0.35818000	-1.94399800	-1.70893400
C	-1.53660900	-1.90070900	0.87377700
O	-2.19640700	-2.24296700	-0.35751100
O	-2.60338500	-1.01124000	-1.01810300
C	-3.76840000	-0.51150400	-0.34539600
C	-3.50253200	-0.37145200	1.14094700
C	-2.43791900	-0.97619100	1.67033000
C	-1.41473100	-3.26044300	1.58839000
C	-4.16483900	0.78406800	-1.07540400
C	-4.50440500	0.38697400	1.96927800
C	-3.23928400	2.01272600	-0.94687600
C	-1.96028900	1.89548500	-1.78947100
C	-4.01540400	3.28164600	-1.33260100
H	5.89875300	2.12095500	-0.46281500
H	5.05773100	2.89868300	0.89141400
H	6.07422600	1.47073700	1.15788500
H	3.62810100	-2.50113300	-2.14245400
H	4.35029800	-2.90374700	-0.59789900
H	2.61668900	-3.17890100	-0.85553600
H	1.28272500	3.88699500	1.86463100
H	0.62707000	2.87981600	0.54336300
H	0.84940100	2.18271400	2.17302300
H	0.19485100	-0.79892200	1.59805700
H	1.18683200	-1.80366300	-2.40131400
H	0.15499400	-3.01359100	-1.62799700
H	-0.52843800	-1.47950600	-2.14166700
H	-4.58472600	-1.23410400	-0.49176900
H	-2.21348800	-0.87873500	2.72874200
H	-0.91032400	-3.13104800	2.54772400
H	-2.40755700	-3.67874000	1.76517600
H	-0.83100600	-3.95459800	0.98073800
H	-4.29945700	0.53657700	-2.13437900
H	-5.15959700	1.04550900	-0.70105100
H	-4.24516900	0.34338500	3.02828800
H	-4.57016600	1.43840100	1.67509000
H	-5.50682500	-0.03866800	1.84775300
H	-2.94047400	2.11076600	0.10350900
H	-1.35065100	2.79835600	-1.68615500
H	-1.35821700	1.03747300	-1.49078300
H	-2.20831500	1.78165500	-2.85078000
H	-3.38457900	4.16995400	-1.23754300
H	-4.35797500	3.22707400	-2.37185200
H	-4.89473000	3.42284500	-0.69740800

Relative Energy = -1194.61283221 a.u.

Number of Imaginary Frequencies = 0

P (%) = 9.34%

Conformer **1-4**:

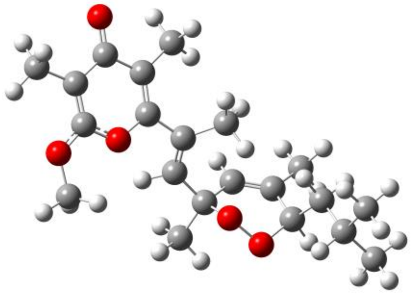

C	4.06048700	0.89418300	0.47406700
C	4.93382000	-0.14809700	0.42495100
C	4.40955800	-1.46565000	0.13298400
C	2.95627000	-1.56807100	-0.11256000
C	2.17958400	-0.46334600	-0.01571600
O	2.74026500	0.76638600	0.26774800
C	6.40531600	0.02281400	0.67017700
C	2.43601300	-2.92494600	-0.49845800
O	5.14017800	-2.46738700	0.07475900
O	4.44699600	2.13868600	0.74943300
C	3.47600200	3.20830500	0.73507900
C	0.70831600	-0.35374300	-0.19073000
C	0.24119500	0.68282700	-0.90600500
C	-0.15856900	-1.39313500	0.48551000
C	-1.20363700	1.03738300	-1.23212400
O	-1.85007300	1.21893700	0.05601900
O	-3.27194800	1.45246000	-0.19481300
C	-3.89774500	0.20281800	-0.53815500
C	-3.14498000	-0.47966900	-1.66291000
C	-1.91855300	-0.06601500	-1.98443700
C	-1.23421900	2.35479400	-2.02202600
C	-4.14761800	-0.68102300	0.69283600
C	-3.85904500	-1.57982100	-2.40085500
C	-4.98653200	-0.02525600	1.80636500
C	-6.43519700	0.24174900	1.37301500
C	-4.95525500	-0.89805500	3.06905800
H	6.69694100	1.07206100	0.64732700
H	6.69344300	-0.39164600	1.64210500
H	6.97437500	-0.52247400	-0.08644400
H	1.49981700	-2.87248000	-1.05116000
H	3.18477300	-3.42227700	-1.11693100
H	2.28371400	-3.56028500	0.38003100
H	3.04697000	3.32460200	-0.26056600
H	2.68661600	3.02358400	1.46361200
H	4.03916900	4.09696100	1.00692000
H	0.96644800	1.36962400	-1.32834400
H	-0.55972000	-2.11524800	-0.23042800
H	0.40018700	-1.94181900	1.24328400
H	-1.00474000	-0.90387100	0.96321100
H	-4.85655100	0.55204500	-0.94065400
H	-1.38783700	-0.50141700	-2.82568700
H	-0.74629500	2.22528300	-2.99140800
H	-2.26422600	2.66651800	-2.18912700
H	-0.70632800	3.13508000	-1.46741300
H	-4.64482500	-1.60102600	0.36427200
H	-3.17767700	-0.97999900	1.09919800
H	-3.26559800	-1.93170700	-3.24642800
H	-4.05968900	-2.43683300	-1.75000100
H	-4.82765300	-1.23492200	-2.77892200
H	-4.51950100	0.93604800	2.04819800
H	-7.00301100	0.70118000	2.18717600
H	-6.49580100	0.91581700	0.51455000
H	-6.93828200	-0.69316000	1.10185200
H	-5.51787400	-0.43189300	3.88304800
H	-5.40074500	-1.88029900	2.87597200
H	-3.93070500	-1.05838800	3.41719300

Relative Energy = -1194.61355798 a.u.
Number of Imaginary Frequencies = 0
P (%) = 13.07%

Conformer **1-5**:

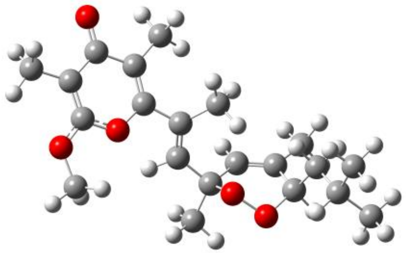

C	4.05824300	0.90217700	0.46492500
C	4.93199700	-0.14042400	0.43116900
C	4.41081600	-1.46017300	0.14426900
C	2.95808000	-1.56732400	-0.10139000
C	2.17988600	-0.46287200	-0.01506100
O	2.73859300	0.77032900	0.25675400
C	6.40279900	0.03112800	0.67947500
C	2.43965000	-2.92812800	-0.47552100
O	5.14436800	-2.46015200	0.09270800
O	4.44220100	2.15116800	0.72356100
C	3.46406100	3.21423100	0.72321200
C	0.70846400	-0.35712800	-0.19152200
C	0.24050900	0.66947800	-0.92039900
C	-0.15717900	-1.38816200	0.49899900
C	-1.20473300	1.01959700	-1.24951000
O	-1.84888700	1.21882600	0.03710200
O	-3.27116900	1.44886800	-0.21459400
C	-3.89747700	0.19450700	-0.53923700
C	-3.14688400	-0.50321700	-1.65611500
C	-1.92113200	-0.09376400	-1.98558900
C	-1.23604600	2.32612500	-2.05732700
C	-4.14448500	-0.67225400	0.70433700
C	-3.86186900	-1.61384300	-2.37739400
C	-4.98135800	-0.00137600	1.81035800
C	-6.43111500	0.25862200	1.37639000
C	-4.94670300	-0.85618400	3.08521200
H	6.67229300	1.08085500	0.78506700
H	6.70774400	-0.49817400	1.58774700
H	6.97860900	-0.40021900	-0.14392500
H	1.50148400	-2.88188700	-1.02547500
H	3.18732900	-3.42851800	-1.09288000
H	2.29175200	-3.55721400	0.40821200
H	3.01633200	3.32447300	-0.26479000
H	2.68907600	3.02682500	1.46648000
H	4.02613400	4.10746500	0.98202700
H	0.96525200	1.35098500	-1.35211900
H	-0.56472800	-2.11529900	-0.20820900
H	0.40444300	-1.93161700	1.25836000
H	-0.99928000	-0.89232100	0.97730100
H	-4.85721000	0.53802100	-0.94448600
H	-1.39196200	-0.54041200	-2.82189300
H	-0.74953100	2.18298400	-3.02549800
H	-2.26610400	2.63616000	-2.22725400
H	-0.70691600	3.11363900	-1.51424100
H	-4.64209200	-1.59682900	0.38956600
H	-3.17361200	-0.96526900	1.11277200
H	-3.27099900	-1.97521500	-3.22075300
H	-4.05832000	-2.46298800	-1.71500500
H	-4.83258100	-1.27555200	-2.75597700
H	-4.51444000	0.96352500	2.03764700
H	-6.99744800	0.72944400	2.18505600
H	-6.49391500	0.92021800	0.50843200
H	-6.93426400	-0.68034400	1.11976300
H	-5.50769800	-0.37881100	3.89380400
H	-5.39206200	-1.84132000	2.90712500
H	-3.92126100	-1.01101600	3.43322300

Relative Energy = -1194.61356345 a.u.
Number of Imaginary Frequencies = 0
P (%) = 13.1%

Table S3. Cartesian Coordinates, Relative Energies, and Boltzmann Populations of Low-energy conformers of (8*R*,11*S*)-**3.**

Conformer **3-1**:

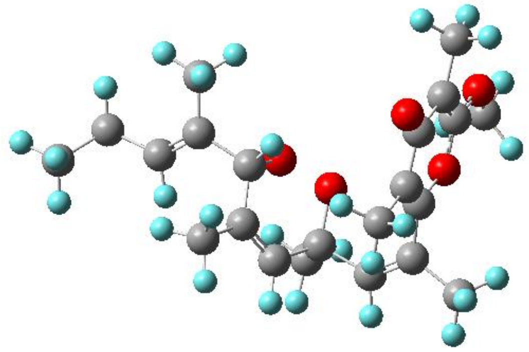

C	3.08316100	1.88770100	0.21602100
C	3.15108400	1.01885600	-0.82985500
O	2.76379700	-0.26458100	-0.75474900
C	2.23012900	-0.76501300	0.41125400
C	2.13903700	-0.00845000	1.52283500
C	2.57464400	1.39899600	1.48248200
O	2.49511200	2.12389900	2.48762900
C	3.52779600	3.31792100	0.10216300
C	1.58592200	-0.52416100	2.82000600
C	1.91649700	-2.21499300	0.27454300
O	3.62279500	1.37055300	-2.02604000
C	3.53801800	0.43420400	-3.12296000
C	3.06036900	-3.13148000	0.65076400
C	0.74925700	-2.70240000	-0.15737200
C	-0.51999600	-2.00057000	-0.60629300
C	-1.50719400	-1.99699000	0.54633000
C	-2.14709700	-0.90049200	0.95726600
C	-1.95389400	0.39231200	0.18925800
O	-1.38431500	0.11900600	-1.11119200
O	-0.15423500	-0.64332400	-0.92631900
C	-1.08181300	-2.70336400	-1.85395800
C	-2.98193100	-0.86335700	2.20855100
C	-3.20254700	1.20284300	-0.14253200
C	-3.02512100	2.69516400	-0.03234400
C	-4.30260800	0.57200800	-0.57119800
C	-5.61076500	1.16541200	-1.01053200
C	-6.78969000	0.70193200	-0.13692500
H	4.45413900	3.48718000	0.66138200
H	3.69924800	3.60303900	-0.93501600
H	2.77412500	3.98118900	0.53351800
H	1.29606400	-1.57228900	2.75456200
H	2.32189700	-0.40782700	3.62028100
H	0.70897700	0.05952800	3.11433400
H	4.15477100	-0.44361700	-2.92893400
H	3.91922100	0.97831900	-3.98290200
H	2.50374300	0.13393200	-3.29251600
H	3.35778000	-2.97508100	1.69247000
H	2.78861500	-4.17991600	0.51893300
H	3.94019500	-2.92144800	0.03401200
H	0.65433900	-3.78492000	-0.20054300
H	-1.63049800	-2.93828800	1.07363500
H	-1.25371300	1.03087900	0.74442800
H	-0.31979200	-2.74877300	-2.63596300
H	-1.39723200	-3.72131300	-1.61310500
H	-1.94495500	-2.15056100	-2.22440700
H	-2.60711900	-0.09656700	2.89630500
H	-4.02383300	-0.61246800	1.99515900
H	-2.95149000	-1.82425300	2.72501500
H	-3.90735100	3.25182200	-0.34562700
H	-2.79250300	2.98122600	0.99931600
H	-2.18068600	3.02377100	-0.64807200
H	-4.26972400	-0.51650900	-0.60415300
H	-5.56916800	2.25630800	-1.02045100
H	-5.80019600	0.85311600	-2.04548200
H	-7.73353100	1.10416900	-0.51425400
H	-6.86937500	-0.38899200	-0.12795100

H	-6.66671600	1.03744900	0.89636600

Relative Energy = -1232.70620312 a.u.
Number of Imaginary Frequencies = 0
P (%) = 88.64%

Conformer **3-2**:

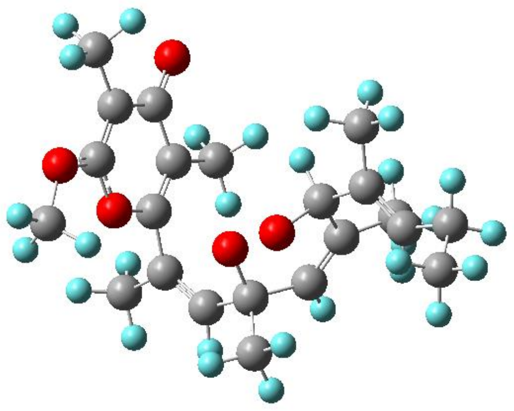

C	-3.08534000	1.85679100	-0.24776700
C	-3.00821100	1.13803200	0.90591200
O	-2.62857200	-0.14895900	0.95461700
C	-2.24608600	-0.80642500	-0.19223400
C	-2.30636300	-0.20742700	-1.39828500
C	-2.75003500	1.19471900	-1.49289200
O	-2.81714200	1.77380400	-2.58928900
C	-3.51458800	3.29590800	-0.27123600
C	-1.91999100	-0.89889900	-2.67406400
C	-1.90690400	-2.22918100	0.09163500
O	-3.31825000	1.65404300	2.09527000
C	-3.07594900	0.87704100	3.28888800
C	-3.07827000	-3.17893700	-0.03505300
C	-0.69428200	-2.66536600	0.44610500
C	0.61021300	-1.92003400	0.66215000
C	1.47212800	-2.06295000	-0.57956900
C	2.07122400	-1.02842000	-1.17261300
C	1.96388900	0.34536500	-0.53957500
O	1.51581400	0.23201300	0.82979300
O	0.27261100	-0.53058100	0.84735900
C	1.30593900	-2.46645200	1.92109100
C	2.77652000	-1.14020600	-2.49684800
C	3.24803600	1.15933400	-0.41683900
C	3.07986700	2.63472700	-0.67321100
C	4.37730800	0.55323400	-0.03053900
C	5.72606500	1.16229300	0.22647500
C	6.19039500	0.95835000	1.67951400
H	-4.50199600	3.40385600	-0.73226300
H	-3.55851800	3.71770000	0.73206500
H	-2.81937300	3.88516400	-0.87441500
H	-1.58046700	-1.91919400	-2.49957200
H	-2.76488300	-0.91935900	-3.36812500
H	-1.11868200	-0.34694400	-3.17321100
H	-3.69989800	-0.01684400	3.30058900
H	-3.34835300	1.53571800	4.10913300
H	-2.02400000	0.59927100	3.35942300
H	-3.49752100	-3.14793300	-1.04556500
H	-2.78326200	-4.20515200	0.18932500
H	-3.88144000	-2.89141200	0.65119100
H	-0.58551200	-3.73443300	0.61284900
H	1.53883500	-3.05902000	-1.00705100
H	1.22187100	0.93247900	-1.09702900
H	0.63069700	-2.40964200	2.77850200
H	1.60019200	-3.50863500	1.77607000
H	2.19989700	-1.87837000	2.12761800
H	2.32520200	-0.46099900	-3.22935900
H	3.83087500	-0.86363600	-2.41966100
H	2.70772800	-2.15565400	-2.89070600
H	3.98556900	3.20557300	-0.47281400
H	2.78789000	2.81739500	-1.71336200
H	2.27842300	3.03879100	-0.04535700
H	4.33212200	-0.52273500	0.13465400
H	6.45492600	0.68670400	-0.44211400
H	5.73433900	2.22701900	-0.01494600
H	7.19415000	1.36543600	1.82695800

H	5.51472400	1.45717700	2.37949800
H	6.21740500	-0.10385400	1.93939700

Relative Energy = -1232.70635021 a.u.
Number of Imaginary Frequencies = 0
P (%) = 5.44%

Conformer **3-3**:

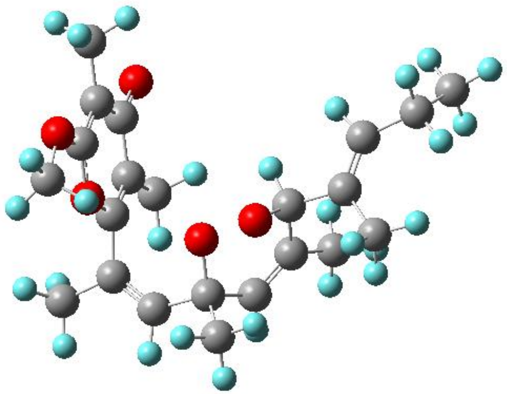

C	-2.58312200	2.28936200	-0.07094200
C	-2.91670900	1.32840300	0.83377800
O	-2.78200700	0.01263900	0.60284600
C	-2.25042000	-0.43608500	-0.58496500
C	-1.91154800	0.41946300	-1.56978100
C	-2.06641100	1.86986700	-1.35852100

O	-1.75425900	2.68312700	-2.24341000
C	-2.74506000	3.75455700	0.21731200
C	-1.35144900	-0.03089800	-2.88815300
C	-2.23917700	-1.92497900	-0.63584300
O	-3.41984200	1.61647400	2.03407800
C	-3.61686300	0.55309000	2.99214300
C	-3.49979900	-2.53116700	-1.21307100
C	-1.23411100	-2.69293600	-0.20390000
C	0.10052500	-2.33058200	0.42244800
C	1.17410500	-2.37466600	-0.64747200
C	2.04836100	-1.38579800	-0.83625000
C	2.04627000	-0.19708600	0.10993500
O	1.29253900	-0.50569200	1.30177600
O	-0.02554300	-0.98044400	0.91181800
C	0.39738400	-3.29033600	1.58705900
C	3.01736500	-1.35302000	-1.98601200
C	3.42187500	0.20284600	0.61903900
C	4.17546800	-0.85165400	1.39088400
C	3.85826400	1.44420000	0.36782500
C	5.17474900	2.06685000	0.73775700
C	5.96836800	2.53780300	-0.49328500
H	-2.92619500	3.93702000	1.27587500
H	-1.84788700	4.29735000	-0.08944500
H	-3.58034300	4.17813800	-0.35055200

H	-2.00093900	0.28778100	-3.70845500
H	-0.37804300	0.43635300	-3.06079000
H	-1.22981600	-1.11249500	-2.93310600
H	-4.37080200	-0.15011800	2.63771700
H	-3.96287900	1.05180700	3.89347700
H	-2.67958000	0.03031400	3.18416400
H	-3.66250900	-2.18611000	-2.23898600
H	-3.45138700	-3.62115700	-1.21461800
H	-4.37503100	-2.22393100	-0.63152600
H	-1.35581900	-3.76832700	-0.31045700
H	1.17148800	-3.24418200	-1.29810000
H	1.58307300	0.66384600	-0.38782900
H	-0.42747400	-3.27849700	2.30373300
H	0.52706800	-4.31111400	1.21975900
H	1.31471600	-2.98315000	2.08898500
H	2.86449900	-0.45338300	-2.59235500
H	4.05556300	-1.32594200	-1.64204900
H	2.88762300	-2.22395500	-2.63042200
H	5.21135000	-0.56940400	1.57388600
H	3.69877800	-1.03465700	2.35843800
H	4.17310900	-1.80561100	0.85522700
H	3.19250400	2.10290300	-0.18873500
H	4.97579600	2.93604100	1.37775400
H	5.78644800	1.38372900	1.33032800

H	6.89120500	3.03984800	-0.19123400
H	6.23563200	1.69304900	-1.13397300
H	5.38435000	3.24161700	-1.09348200

Relative Energy = -1232.70876047 a.u.

Number of Imaginary Frequencies = 0

P (%) = 5.92%

Table S4. Cartesian Coordinates, Relative Energies, and Boltzmann Populations of Low-energy conformers of (8*R*,11*S*)-**5.**

Conformer **5-1**:

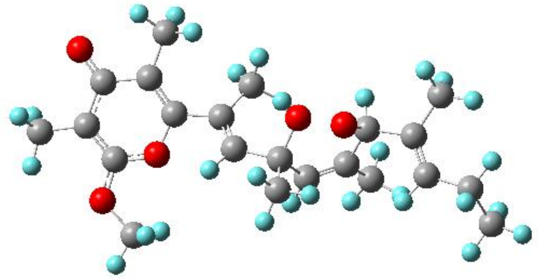

C	-5.40238200	0.23877100	0.12152400
C	-4.39941100	1.15822600	0.11726000
O	-3.09728900	0.83424600	0.08840100
C	-2.69084200	-0.48564500	0.05039500
C	-3.60355400	-1.48541000	0.02009000
C	-5.04487700	-1.16319800	0.07649400
O	-5.90326500	-2.05982700	0.07244500
C	-6.85190200	0.62760700	0.16832100
C	-3.24652500	-2.94087700	-0.10771900
O	-4.63003400	2.46970900	0.15628300
C	-1.20853600	-0.58610200	0.04117400
C	-0.57254400	-1.62364800	0.94040200
C	-0.52954500	0.28145000	-0.72771600
C	0.97664400	0.40404500	-0.90566600
C	1.28232700	1.27831700	-2.13018200
C	1.63231800	0.94280700	0.35156800
O	1.43842000	-0.95130400	-1.13552200
C	2.70912200	0.38587700	0.91054100
C	3.25829200	0.82736300	2.24029300
C	3.35304500	-0.81091900	0.23724300
C	4.87227500	-0.80452000	0.10023700
C	5.52024500	-2.15151200	0.29267800
C	5.49981400	0.32503200	-0.24938000
O	2.89959300	-0.92638400	-1.13007900
C	-3.51869600	3.39218000	0.14899600
C	6.96478400	0.54274700	-0.50077000
C	7.57792600	1.57861100	0.45772700
H	-7.39062400	0.19506100	-0.67953900
H	-7.32747000	0.23972900	1.07432900
H	-6.97718900	1.70905800	0.14743200
H	-3.20005500	-3.43198400	0.86958300
H	-4.03347900	-3.44142300	-0.67311800
H	-2.29457000	-3.09358800	-0.61333100
H	-0.32889200	-2.54002900	0.39957700
H	0.35736700	-1.23695800	1.35587400
H	-1.24029900	-1.88384300	1.76281800
H	-1.09953000	0.99445400	-1.31331300
H	0.75857500	0.89076300	-3.00797000
H	0.95531800	2.30634400	-1.95451600
H	2.35430300	1.28163600	-2.32382300
H	1.15221200	1.80090500	0.81266800
H	3.28672300	-0.01406000	2.94221000
H	4.28232900	1.19868900	2.15419800
H	2.63698500	1.61114700	2.67682300
H	3.05176000	-1.71900300	0.77787200
H	6.59553100	-2.13369600	0.12041800
H	5.34598800	-2.52245500	1.30865900
H	5.07983900	-2.88561800	-0.39093900
H	4.88804100	1.21941100	-0.36217100
H	-3.97524500	4.37605900	0.21498400
H	-2.95048400	3.30284200	-0.77722000
H	-2.86768700	3.21990800	1.00621100
H	7.52302600	-0.39306800	-0.43448300
H	7.08769500	0.90463300	-1.52962900
H	8.62848100	1.75801800	0.21456600
H	7.05065200	2.53496200	0.39483800
H	7.52342000	1.23336900	1.49370700

Relative Energy = -1232.70532258 a.u.

Number of Imaginary Frequencies = 0

P (%) = 37.37%

Conformer **5-2**:

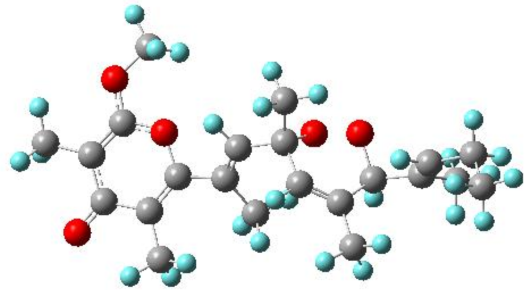

C	5.43227100	0.02354400	-0.03762500
C	4.51342100	1.02336700	0.04905700
O	3.18978300	0.81050400	0.10734500
C	2.66881700	-0.46847700	0.07581600
C	3.48882800	-1.54043800	-0.03776400
C	4.95290200	-1.34176400	-0.07595400
O	5.72724600	-2.30895800	-0.15142400
C	6.90948300	0.28816200	-0.08802000
C	3.00522000	-2.95845600	-0.16770600
O	4.85710700	2.30917500	0.10024900
C	1.18677600	-0.44195700	0.17166600
C	0.52208300	-1.46112400	1.07234300
C	0.53317900	0.51083700	-0.51521300

C	-0.96687500	0.77108600	-0.56417500
C	-1.24713600	2.01672700	-1.41568500
C	-1.72938700	-0.43813600	-1.06758000
O	-1.33626600	1.02361300	0.81822000
C	-2.80626700	-0.93244200	-0.45366300
C	-3.46026300	-2.22331700	-0.86596700
C	-3.33424500	-0.23364300	0.78427600
C	-4.84002200	-0.00682800	0.87626700
C	-5.40940600	-0.16880300	2.26209500
C	-5.51661700	0.38736800	-0.20940200
O	-2.79546300	1.10333700	0.88019700
C	3.82913700	3.32279900	0.15318000
C	-6.97439700	0.72865400	-0.33123700
C	-7.70862100	-0.17711700	-1.33527100
H	7.36344700	-0.27275200	-0.90891600
H	7.12297400	1.34765900	-0.22221000
H	7.39901700	-0.04758100	0.83200500
H	1.99534800	-3.02154800	-0.56846900
H	3.68450700	-3.49608100	-0.83083000
H	3.02841100	-3.48070400	0.79421400
H	1.23128600	-1.86964900	1.79228700
H	-0.29671100	-0.99295300	1.61511400
H	0.10427900	-2.29426400	0.50130400
H	1.12092500	1.19311400	-1.11917900

H	-0.65802100	2.86158400	-1.04934600
H	-0.97911600	1.83111800	-2.45903100
H	-2.30567000	2.26921300	-1.36706000
H	-1.32787000	-0.92251100	-1.95282500
H	-3.47406000	-2.93037500	-0.02837300
H	-4.49968300	-2.07476500	-1.16799100
H	-2.91846200	-2.68798300	-1.69152200
H	-3.00910800	-0.80068200	1.66803800
H	-6.47019000	0.07224400	2.31681100
H	-5.27699700	-1.19687700	2.61668300
H	-4.87815400	0.47909600	2.96786600
H	-4.95757200	0.47008200	-1.14087000
H	3.21912200	3.29953900	-0.75034100
H	4.37050500	4.26290400	0.21554300
H	3.19985200	3.18993200	1.03316100
H	-7.47599000	0.68365200	0.63745600
H	-7.05724300	1.76786300	-0.67461900
H	-8.75209900	0.13012700	-1.44359300
H	-7.24105000	-0.13157300	-2.32315800
H	-7.69422700	-1.21945100	-1.00546700

Relative Energy = -1232.70494114 a.u.
Number of Imaginary Frequencies = 0
P (%) = 25.29%

Conformer **5-3**:

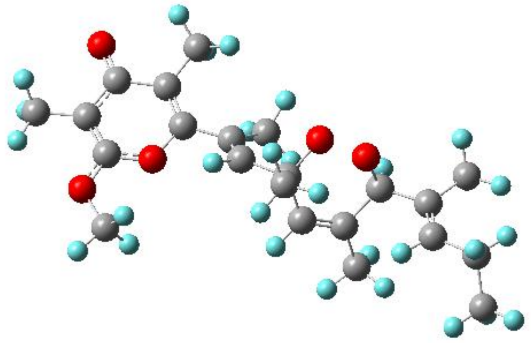

C	5.40244600	0.23870300	-0.12138000
C	4.39950600	1.15820000	-0.11721100
O	3.09737500	0.83428700	-0.08845500
C	2.69087500	-0.48559200	-0.05040300
C	3.60354500	-1.48538100	-0.02004200
C	5.04489600	-1.16323700	-0.07640500
O	5.90322300	-2.05992800	-0.07237800
C	6.85197500	0.62753000	-0.16812100
C	3.24643100	-2.94082400	0.10771300
O	4.63020200	2.46966600	-0.15637200
C	1.20855800	-0.58598800	-0.04135000
C	0.57262800	-1.62333200	-0.94085300
C	0.52952600	0.28143700	0.72764700
C	-0.97663800	0.40399700	0.90555100
C	-1.28241800	1.27812200	2.13012500
C	-1.63234600	0.94278200	-0.35166400
O	-1.43843600	-0.95145300	1.13530200
C	-2.70921200	0.38590900	-0.91057100
C	-3.25842400	0.82746100	-2.24028900
C	-3.35313300	-0.81091200	-0.23731500
C	-4.87233800	-0.80454000	-0.10018300
C	-5.52028600	-2.15157500	-0.29239100
C	-5.49988500	0.32503900	0.24932600
O	-2.89956000	-0.92652700	1.12995700
C	3.51891800	3.39221700	-0.14874800
C	-6.96483600	0.54275000	0.50083200
C	-7.57799600	1.57877900	-0.45746300
H	7.32755900	0.23979300	-1.07417900
H	6.97726300	1.70897400	-0.14706900
H	7.39068600	0.19487100	0.67968600
H	3.20020700	-3.43196300	-0.86958200
H	4.03320500	-3.44139800	0.67334000
H	2.29434200	-3.09348500	0.61308300
H	0.32902700	-2.53988100	-0.40029400
H	-0.35730000	-1.23659500	-1.35623500
H	1.24042500	-1.88327300	-1.76332000
H	1.09949000	0.99434200	1.31338200
H	-0.75870800	0.89049200	3.00790300
H	-0.95539300	2.30616500	1.95457700
H	-2.35440000	1.28142800	2.32369900
H	-1.15220200	1.80081700	-0.81283800
H	-3.28712400	-0.01395800	-2.94219400
H	-4.28236600	1.19901200	-2.15407500
H	-2.63699900	1.61110800	-2.67689400
H	-3.05188600	-1.71892800	-0.77808300
H	-6.59549300	-2.13385100	-0.11964100
H	-5.34645400	-2.52245900	-1.30846600
H	-5.07951300	-2.88567200	0.39099100
H	-4.88813200	1.21945200	0.36196100
H	3.97554000	4.37607000	-0.21457700
H	2.95080600	3.30272000	0.77750600
H	2.86781300	3.22017400	-1.00593500
H	-7.52310100	-0.39304200	0.43442000
H	-7.08767500	0.90446900	1.52975700
H	-8.62853800	1.75816500	-0.21423700
H	-7.05071200	2.53511300	-0.39444300
H	-7.52354100	1.23371100	-1.49350300

Relative Energy = -1232.70532262 a.u.

Number of Imaginary Frequencies = 0

P (%) = 37.34%
